# Supplementary material for: Metabolic rewiring of macrophages by epidermal-derived lactate promotes sterile inflammation in the murine skin
Source: EMBO J. 2024 Feb 28;43(7):1113–34. doi: 10.1038/s44318-024-00039-y (PMC10987662; doi:10.1038/s44318-024-00039-y)
Supplement: Supplementary file 1 — Appendix [file 44318_2024_39_MOESM1_ESM.pdf]

Table of contents

S.No.	Figure	Page Number
1	Appendix Figure S1	2-3
2	Appendix Figure S2	4-5
3	Appendix Figure S3	6
4	Appendix Figure S4	7-9
5	Appendix Figure S5	10
6	Appendix Figure S6	11-12
7	Appendix Figure S7	13
8	Appendix Figure S8	14-15

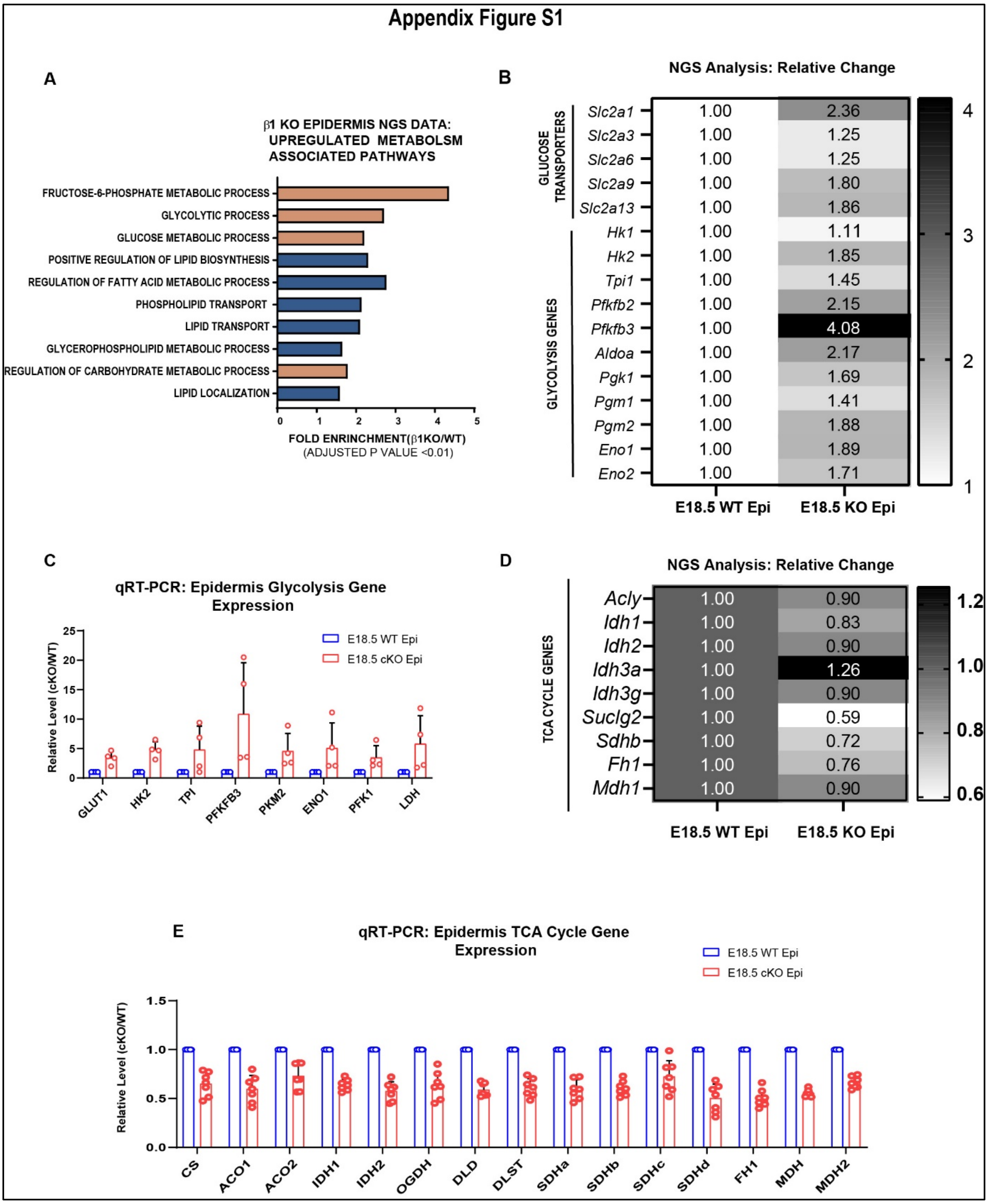

Appendix Fig. S1, related to Fig. 1: NGS data summary and validation of glycolysis and TCA cycle gene expression in epidermal compartment in itgβ1 cKO skin.

A Metabolic pathway upregulated in GSEA analysis of epidermal compartment in E18.5 cKO skin compared to controls (A).

B Relative transcript expression of major glucose transporters and glycolytic enzyme genes in the epidermal compartment of cKO and control skin in NGS data (B) (N=2).

C qPCR showing increased transcription of genes associated with glucose uptake and glycolysis in cKO epidermis compared to control (C) (N=4).

D Relative transcript expression of TCA cycle enzyme genes in the epidermal compartment of cKO and control skin in NGS data (E) (N=2).

E qPCR showing decreased transcription of genes associated with the TCA cycle in cKO epidermis compared to control (F) (N=3).

Scale bars: 50 µm. All graphs are presented as mean ± SEM. WT experimental values are normalized to 1 for relative quantification. N represents the number of biological replicates.

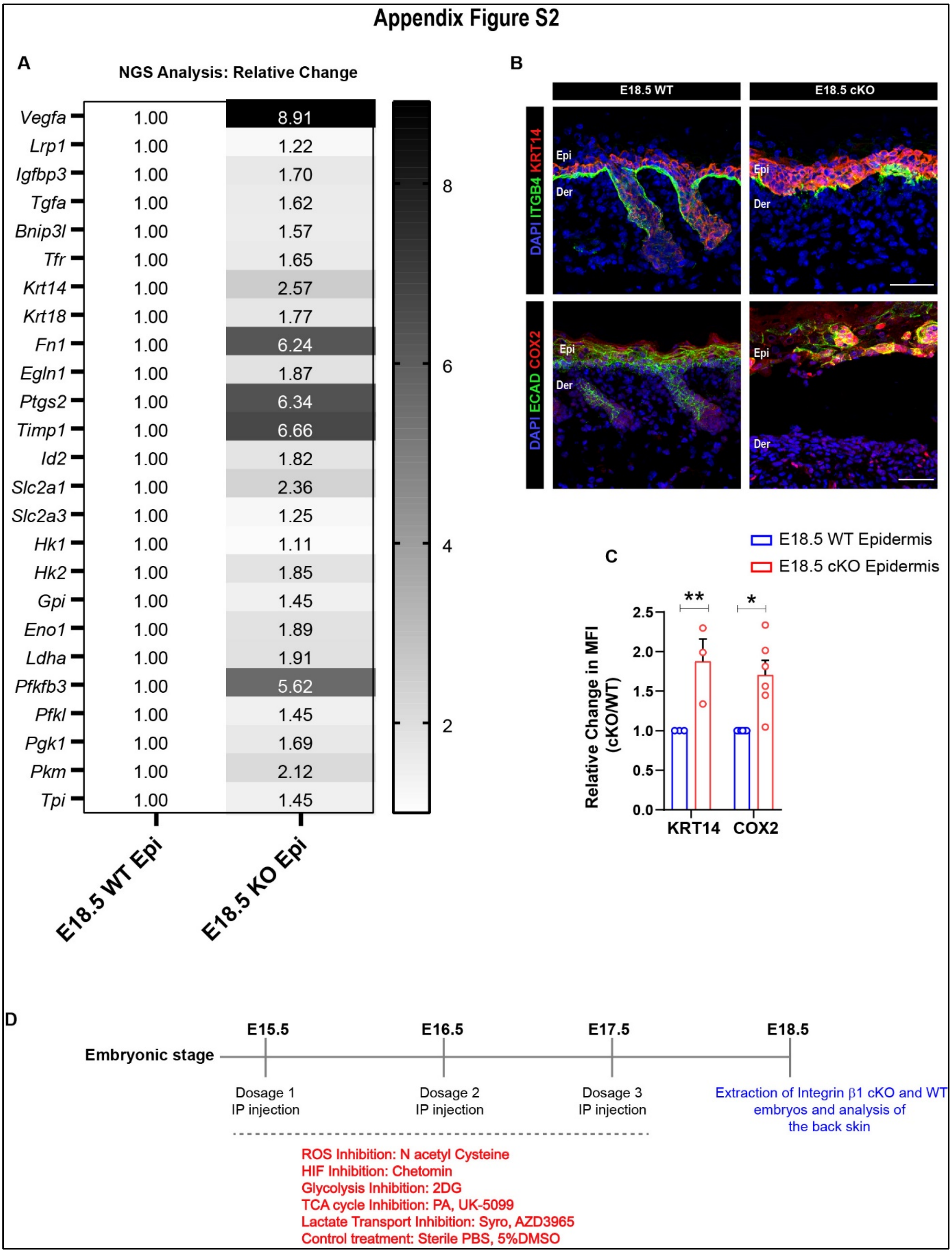

Appendix Fig. S2, related to Fig. 2: Increased HIF1α target expression in epidermal compartment of the itgβ1 cKO skin.

A Relative transcript expression of downstream targets of HIF1α in the epidermal compartment of cKO and control skin in NGS data (E) (N=2).

B,C cKO skin showing increased expression of HIF1α targets KRT14 (N=3) and COX2 (N=6), quantified in (C), compared to controls.

D Schematic depicting dose schedule of various drugs used in the study in pregnant dams carrying cKO and control embryos.

Scale bars: 50 μm. *$p \leq 0.05$, **$p \leq 0.01$ (student's t test). All graphs are presented as mean ± SEM. WT experimental values are normalized to 1 for relative quantification. N represents the number of biological replicates.

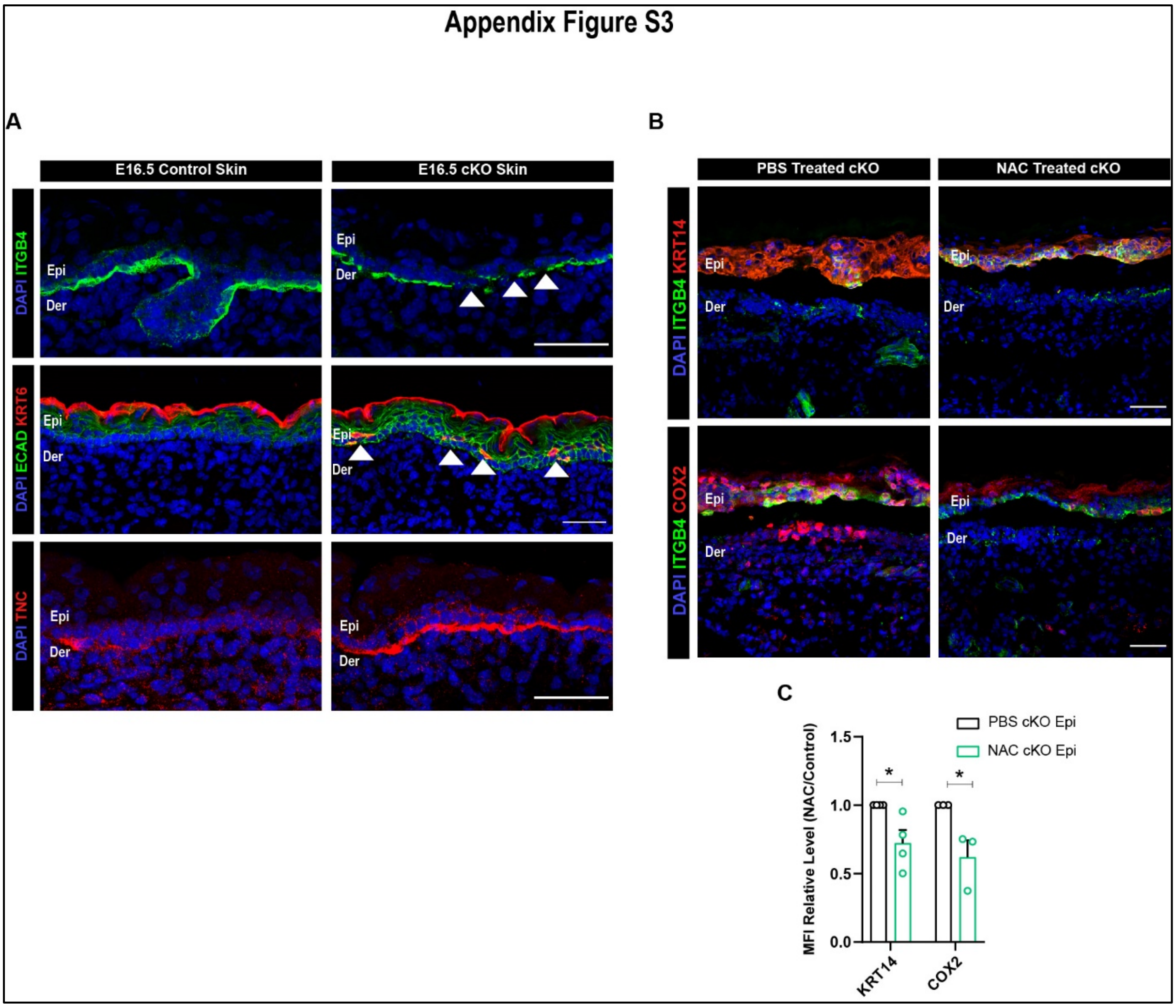

Appendix Fig. S3, related to Fig. 3: Increased ROS response in epidermal compartment of the itgβ1 cKO skin.

A E16.5 cKO skin showing aberrant expression of integrin β4 (A, top), increased expression of stress keratin, keratin 6 (A, middle), and mechanical stress induced ECM component Tenascin C (A, bottom) compared to controls (N=3). White arrowheads show an increased/aberrant staining pattern.

B,C E18.5 cKO skin showing decreased expression of HIF1α targets KRT14 (N=4) and COX2 (N=3) (B), quantified in (C) after treatment with ROS inhibitor, NAC, compared to DMSO treated controls.

Scale bars: 50 μm. *p ≤ 0.05 (student's t test). All graphs are presented as mean ± SEM. WT experimental values are normalized to 1 for relative quantification. N represents the number of biological replicates.

Appendix Figure S4

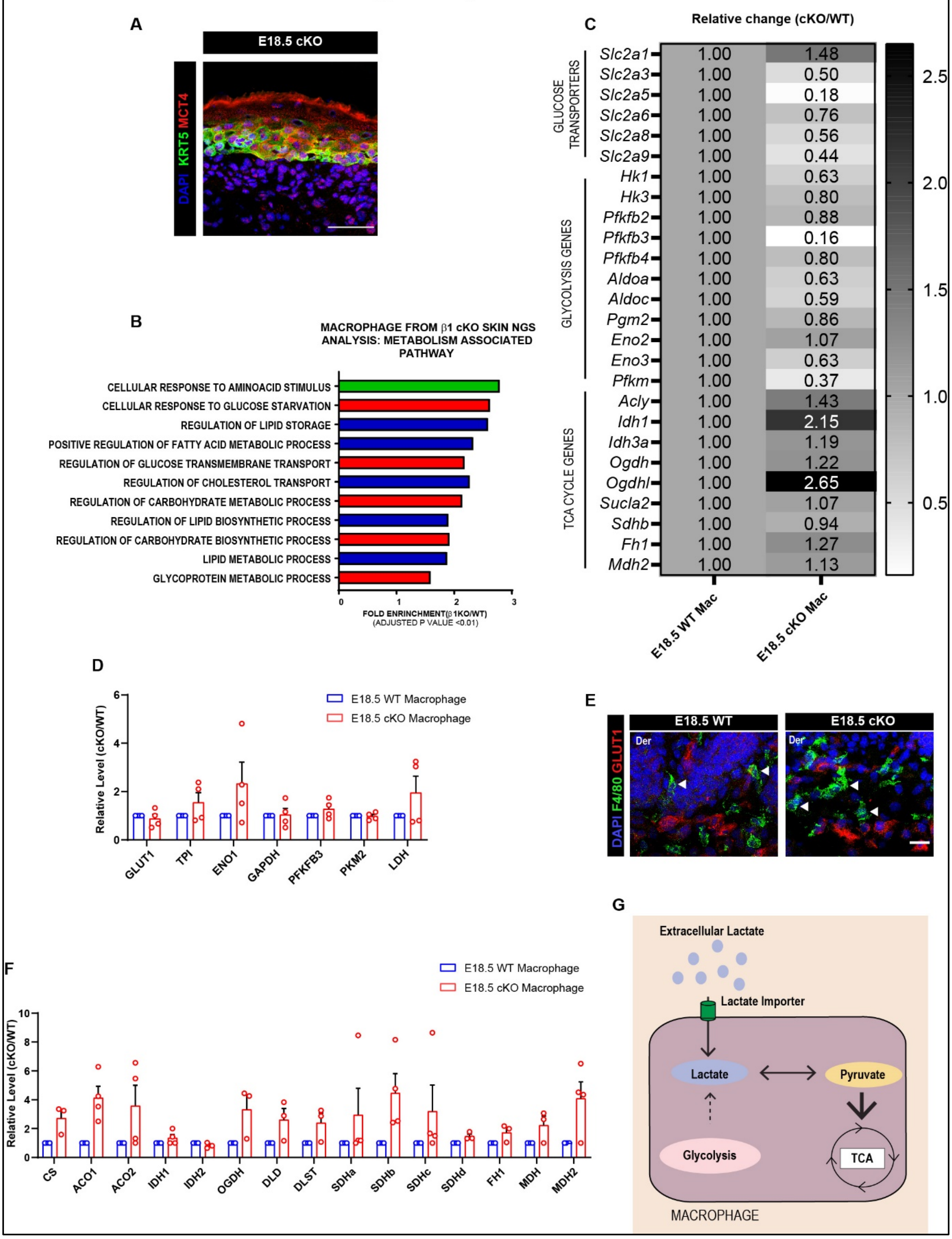

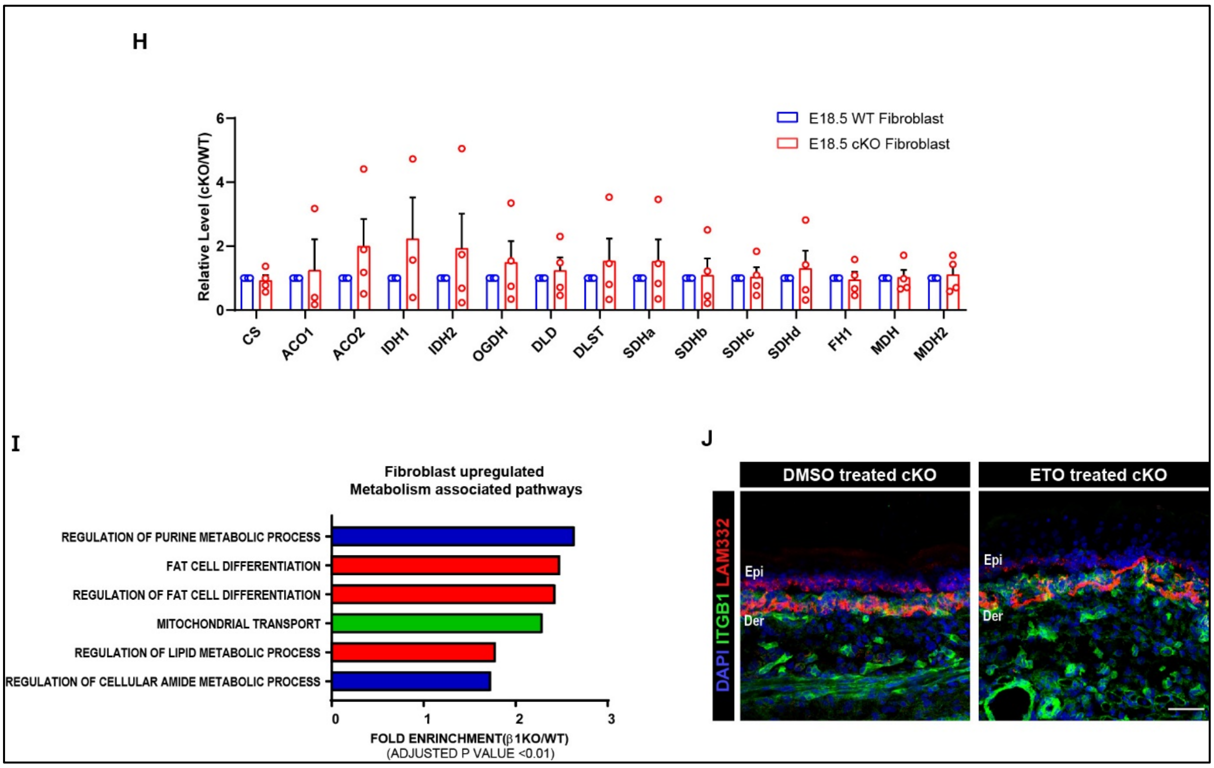

Appendix Fig. S4, related to Fig. 4: Macrophages upregulate TCA cycle genes and downregulate glycolysis in the itgβ1 cKO skin.

A E18.5 cKO skin showing colocalization of lactate transporter MCT4 with epidermal basal keratinocyte marker KRT5 (N=3).

B Metabolic pathway upregulated in GSEA analysis of macrophage compartment in E18.5 cKO skin compared to controls (A).

C Relative transcript expression of major glucose transporters, glycolytic enzymes, and TCA cycle enzyme genes from the NGS data of FACS sorted macrophages from cKO and control skin in the NGS data (B) (N=2).

D qPCR showing non-significant change in the transcription of glycolysis enzymes and glucose transporters in FACS sorted macrophages obtained from cKO skin compared to controls (C) (N=4).

E E18.5 WT and cKO skin showing that macrophages in the dermis do not colocalize with GLUT1 (D) (N=3). White arrowheads indicate the localization of macrophages (green).

F qPCR showing increased transcription of TCA cycle genes in FACS sorted macrophages isolated from E18.5 cKO skin dermis compared to controls(E) (N=4).

G Schematic showing macrophages as potential sinks for lactate since lactate, in one step reaction, can be converted to pyruvate to drive the TCA cycle in the macrophage compartment (F). Scale bars: 50 µm.

H qPCR showing non-significant change in the expression of TCA cycle enzymes in FACS sorted fibroblasts isolated from cKO skin dermis compared to controls (N=4).

I Metabolic pathways upregulated in GSEA analysis of FACS sorted fibroblasts isolated from E18.5 cKO skin compared to controls (B).

J LAM332 staining suggest no obvious difference in the basement membrane spread in the cKO skin treated with etomoxir compared to DMSO controls (N=3). Scale bars: 50 µm.

All graphs are presented as mean ± SEM. WT experimental values are normalized to 1 for relative quantification. N represents the number of biological replicates.

Appendix Figure S5

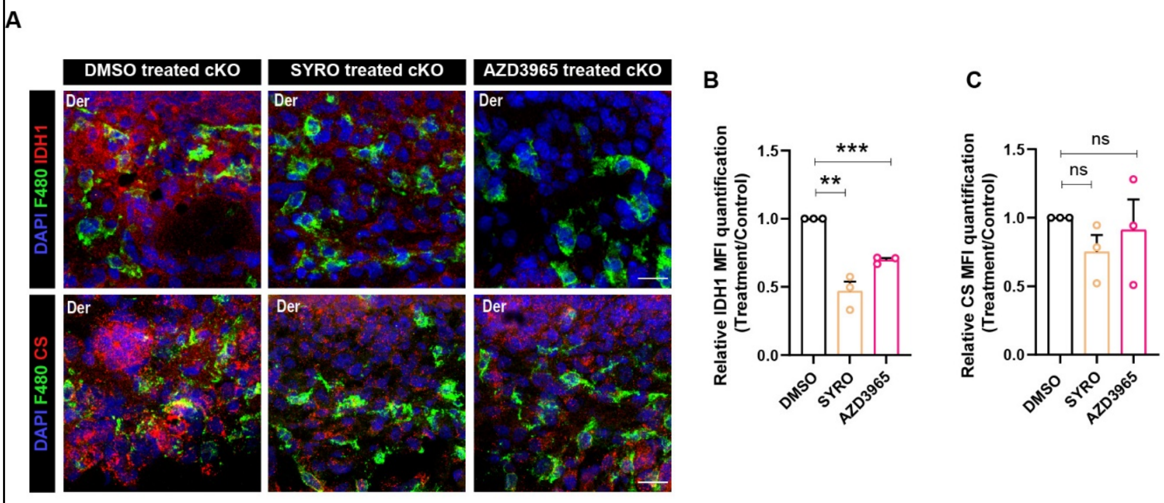

Appendix Fig. S5, related to Fig. 5: Inhibition of lactate transport between epidermis and macrophages attenuates expression of TCA cycle enzymes in the dermal macrophages of cKO skin.

A,B,C Macrophages in E18.5 cKO skin showing decreased expression of TCA cycle enzymes IDH1 (A, top), quantified in (B), and CS (A, bottom) , quantified in (C), after SYRO and AZD3965 treated samples compared to controls (N=3). Scale bars: 20 µm.

*p ≤ 0.05, ***p ≤ 0.001, ns=not significant (student's t test). All graphs are presented as mean ± SEM. WT experimental values are normalized to 1 for relative quantification. N represents the number of biological replicates.

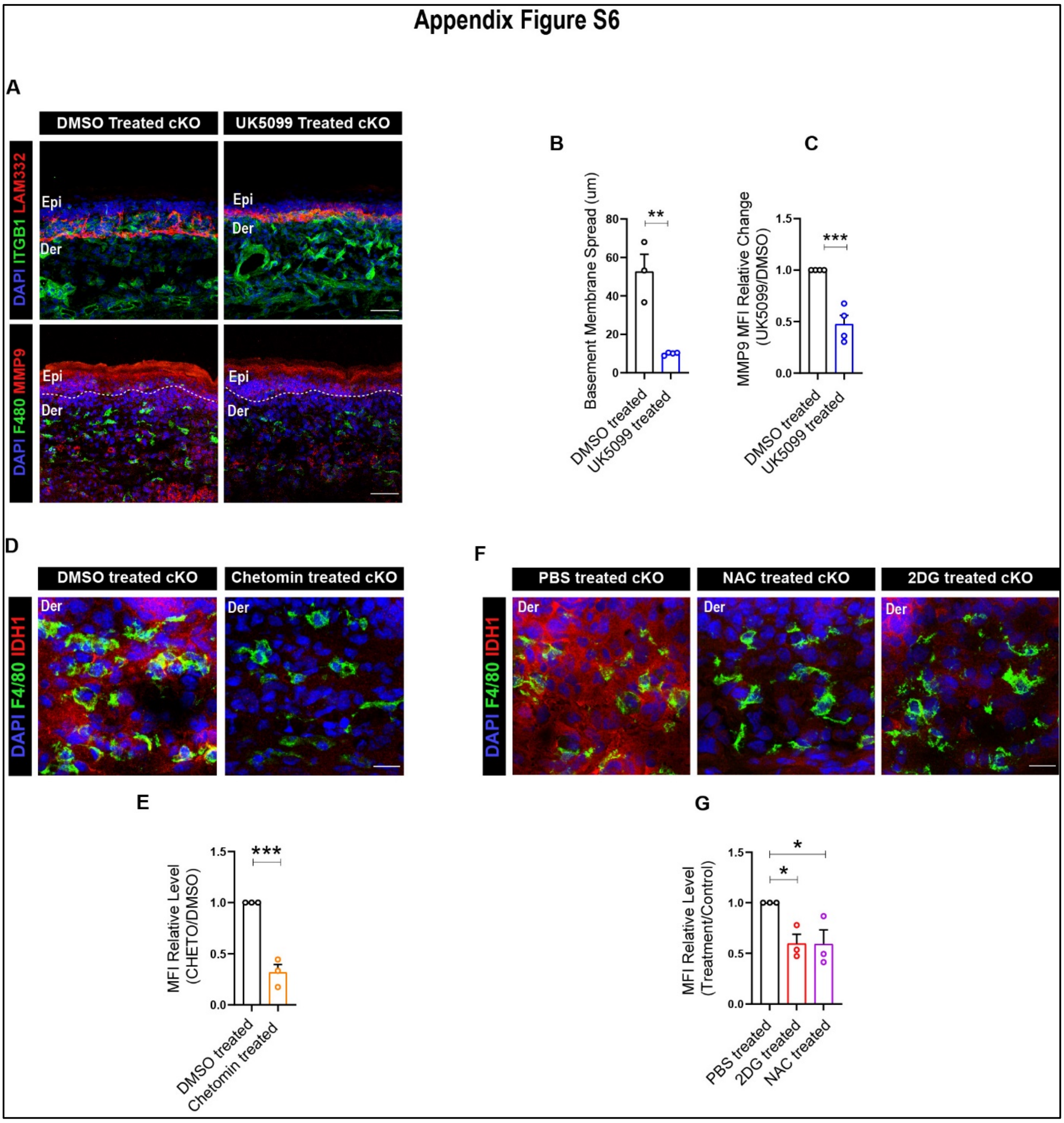

Appendix Fig. S6, related to Fig. 6: Inhibition of TCA cycle using UK-5099 and ROS-HIF1α-glycolysis axis attenuates pro-remodeling fate of dermal macrophages in cKO skin.

A,B,C cKO skin reduced basement membrane disruption (A, top), quantified in B (N=4), and MMP9 expression (A, bottom), quantified in (C) (N=4), after treatment with TCA cycle inhibitor, UK-5099, compared to controls. Scale bars: 50 μm.

D,E Macrophages in E18.5 cKO skin showing reduced expression of TCA cycle enzyme IDH1 in Chetomin (D) treated skin, quantified in (E), compared to controls (N=3). Scale bar: 20μm.

F,G Macrophages in E18.5 cKO skin showing reduced expression of TCA cycle enzyme IDH1 in NAC and 2DG treated skin (F), quantified in (G) compared to controls (N=3). Scale bar: 20μm.

$^{*}p \leq 0.05$, $^{**}p \leq 0.01$, $^{***}p \leq 0.001$ (student's t test). All graphs are presented as mean ± SEM. WT experimental values are normalized to 1 for relative quantification. N represents the number of biological replicates.

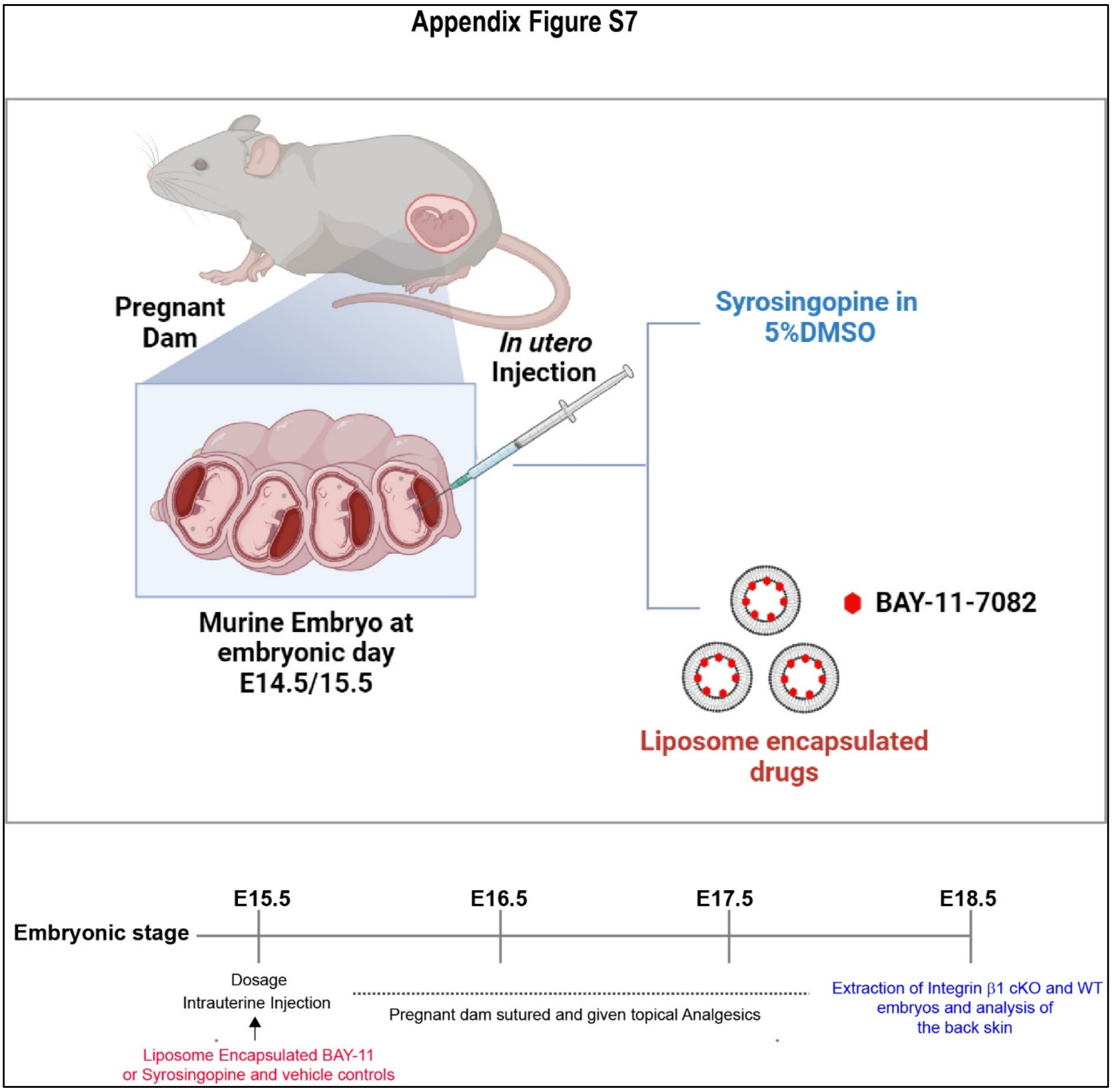

Appendix Fig. S7, related to Fig. 7: Schematic for *in utero* injection and dose schedule.

Schematic showing in utero administration paradigm for Syrosingopine and liposome-encapsulated BAY-11-7082. The schematic was generated using BIORENDER.

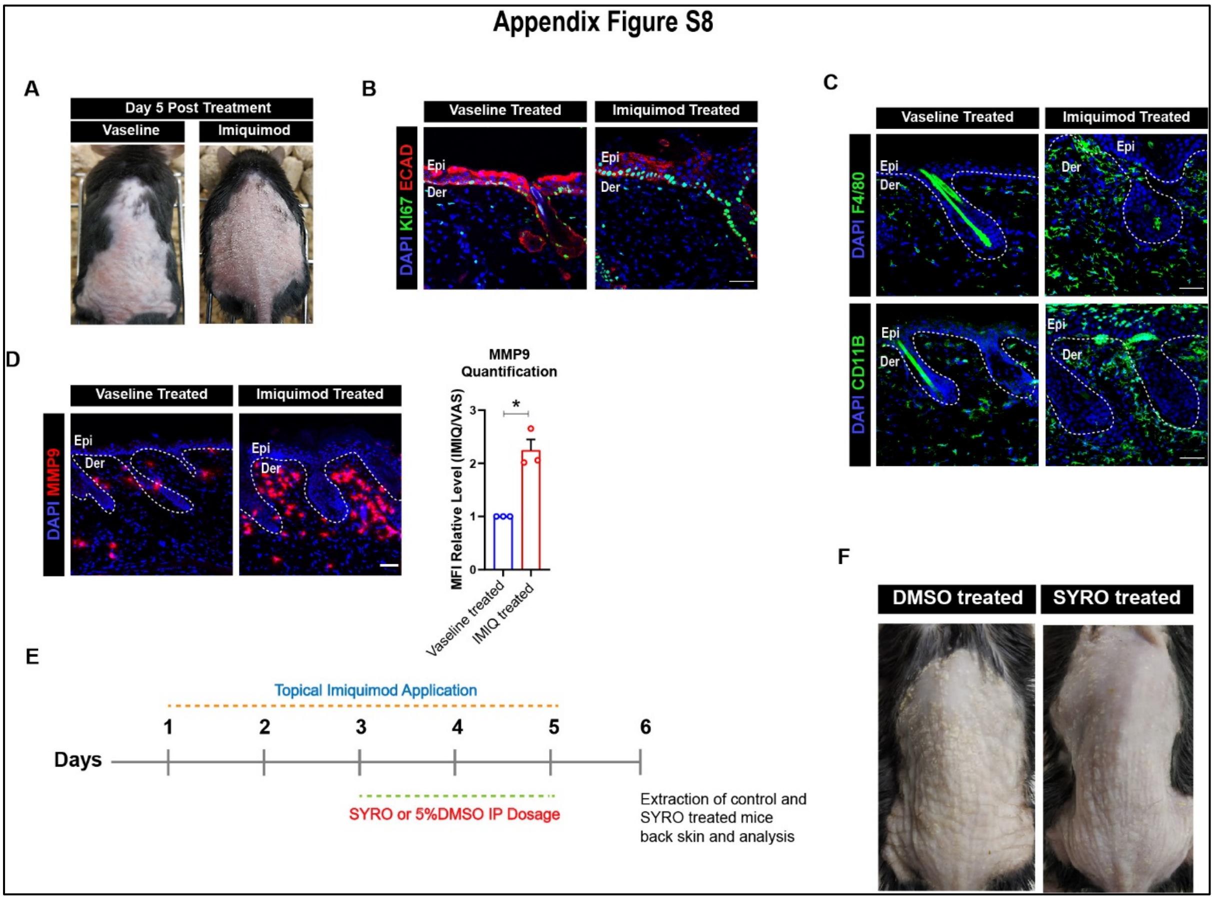

Appendix Fig. S8, related to Fig. 8: Imiquimod treatment induces macrophage burden and increased MMP9 expression in adult mice.

A Adult mice (2 months) showing increase in epidermal plaques upon topical treatment with imiquimod compared to Vaseline treated controls (A) (N=3).

B Imiquimod treated skin showing increased Ki67+ epidermal cells compared to Vaseline treated controls (N=3).

C Imiquimod treated skin showing increase in the number of macrophages (F4/80) (top) and monocytes (CD11B) (bottom) in imiquimod treated skin, compared to controls (D) (N=3).

D Imiquimod treated skin showing increase the expression of MMP9 in imiquimod treated skin compared to controls (N=3).

E Schematic showing dose schedule for intraperitoneal administration of SYRO or 5%DMSO in Imiquimod treated skin in adult (2 months) mice (G).

F Adult (2 months) mice skin showing reduced epidermal plaques in SYRO treated skin, compared to controls (N=4).

Scale bars: 50 µm. *$p \leq 0.05$ (student's t test). All graphs are presented as mean ± SEM. WT experimental values are normalized to 1 for relative quantification. N represents the number of biological replicates.
