# Supplementary material for: Metabolic rewiring of macrophages by epidermal-derived lactate promotes sterile inflammation in the murine skin
Source: EMBO J. 2024 Feb 28;43(7):1113–34. doi: 10.1038/s44318-024-00039-y (PMC10987662; doi:10.1038/s44318-024-00039-y)
Supplement: Supplementary file 2 — Source Data Fig. 1 [file 44318_2024_39_MOESM2_ESM.zip › Figure 1/1D/Annotated Western Data.pptx]

## Slide 1
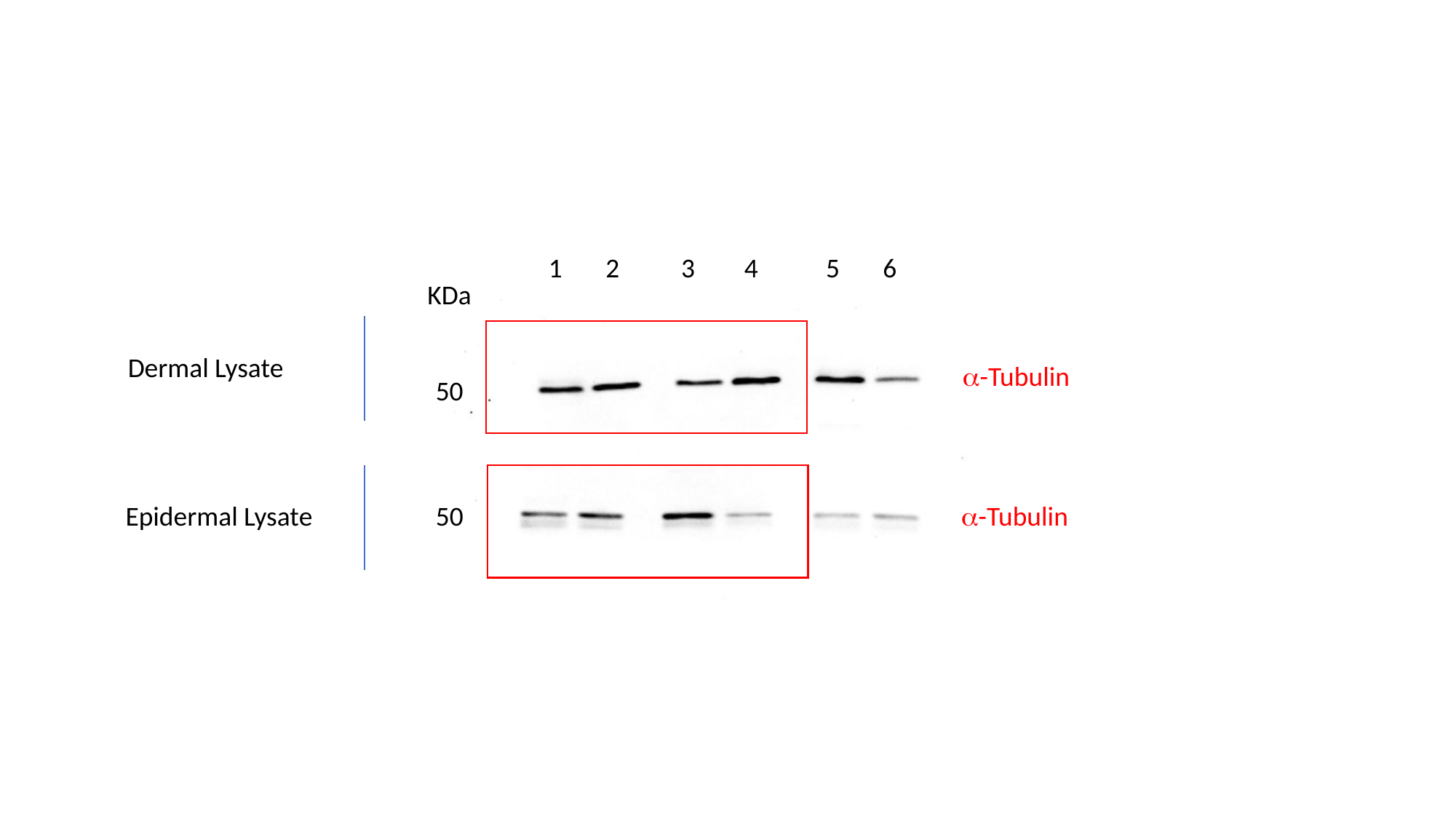

1 2 3 4 5 6
KDa
Dermal Lysate
a-Tubulin
50
Epidermal Lysate
50
a-Tubulin

## Slide 2
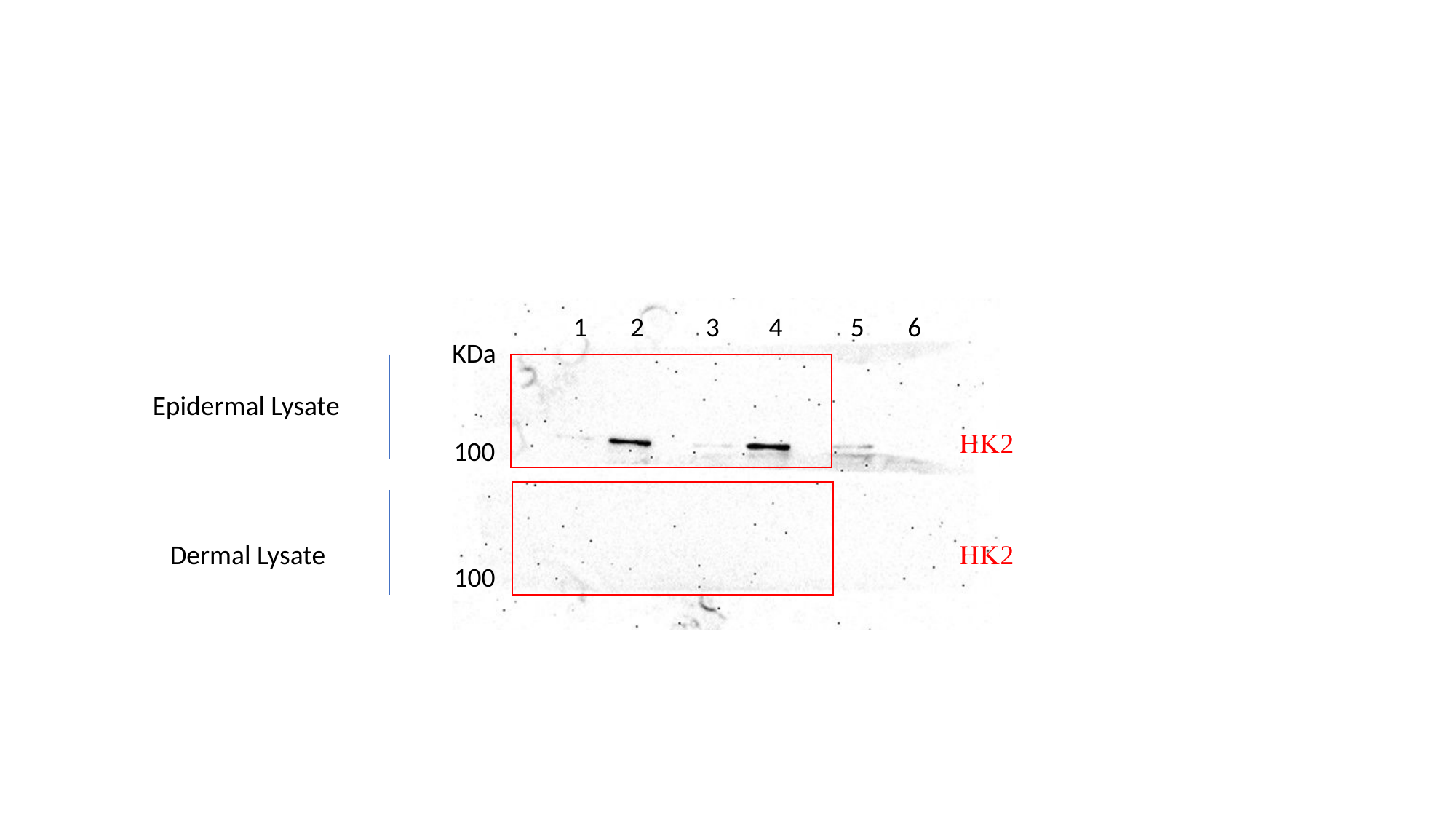

1 2 3 4 5 6
KDa
Epidermal Lysate
HK2
100
Dermal Lysate
HK2
100
