# Supplementary figures and images for: Metabolic rewiring of macrophages by epidermal-derived lactate promotes sterile inflammation in the murine skin (part 1 of 2)
Source: EMBO J. 2024 Feb 28;43(7):1113–34. doi: 10.1038/s44318-024-00039-y (PMC10987662; doi:10.1038/s44318-024-00039-y)

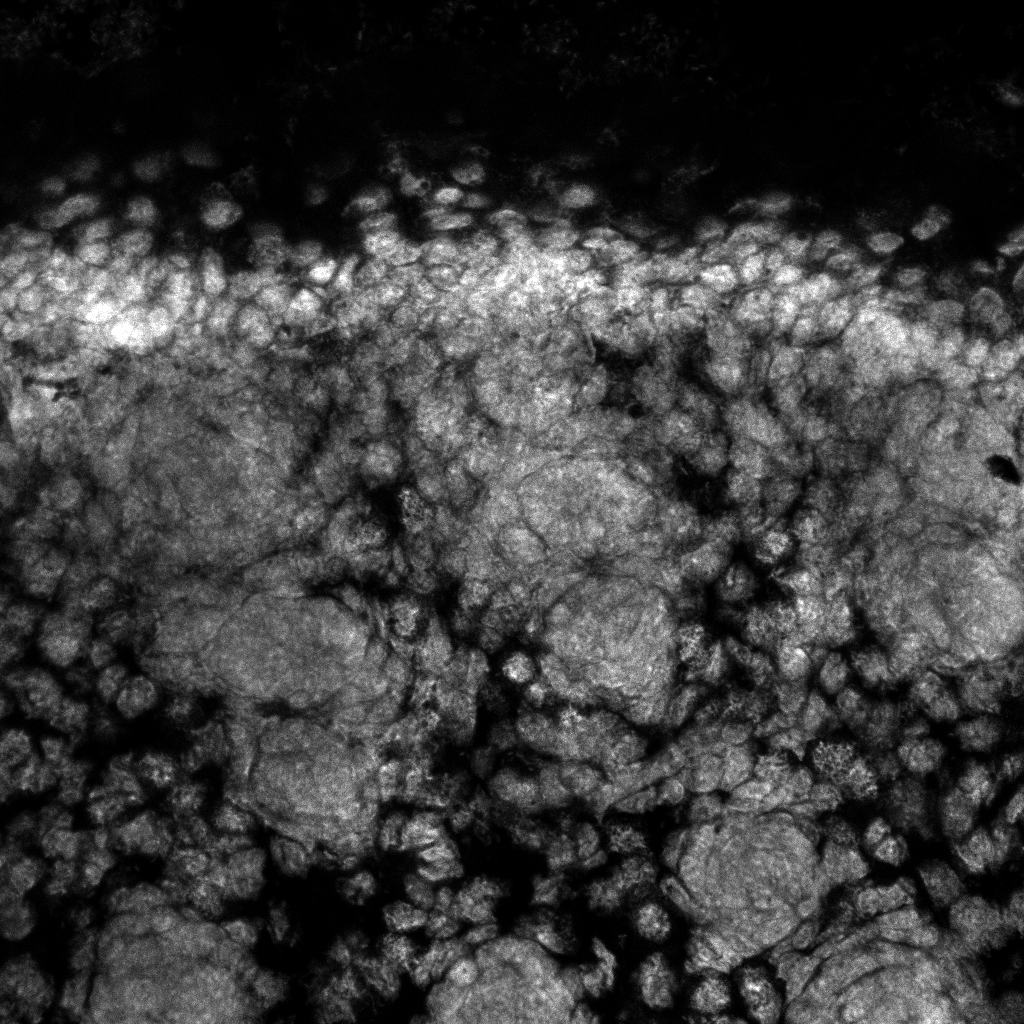

Supplement: Supplementary file 2 — Source Data Fig. 1 [file 44318_2024_39_MOESM2_ESM.zip › Figure 1/1H/E18.5 WT IDH1red F480green 40X.tif]

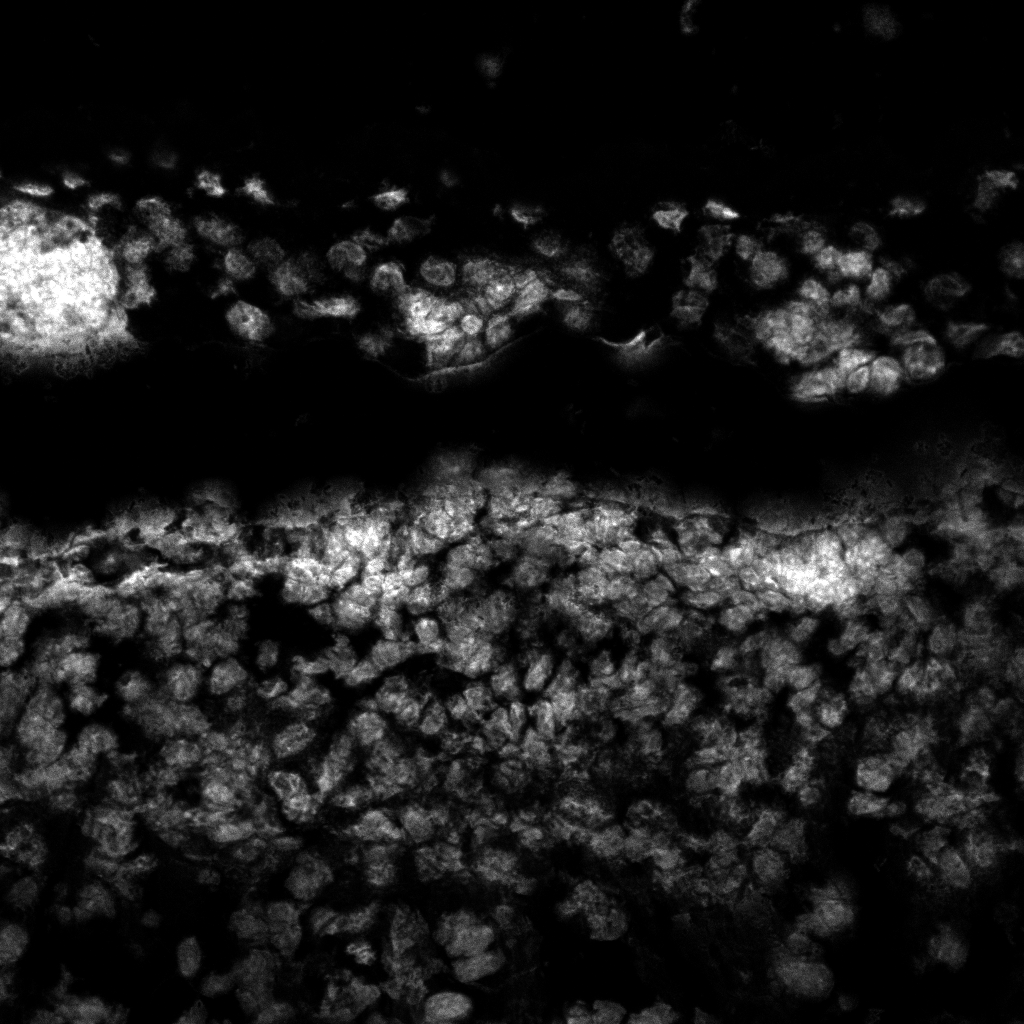

Supplement: Supplementary file 2 — Source Data Fig. 1 [file 44318_2024_39_MOESM2_ESM.zip › Figure 1/1H/E18.5 KO IDH1red F480green 40X.tif]

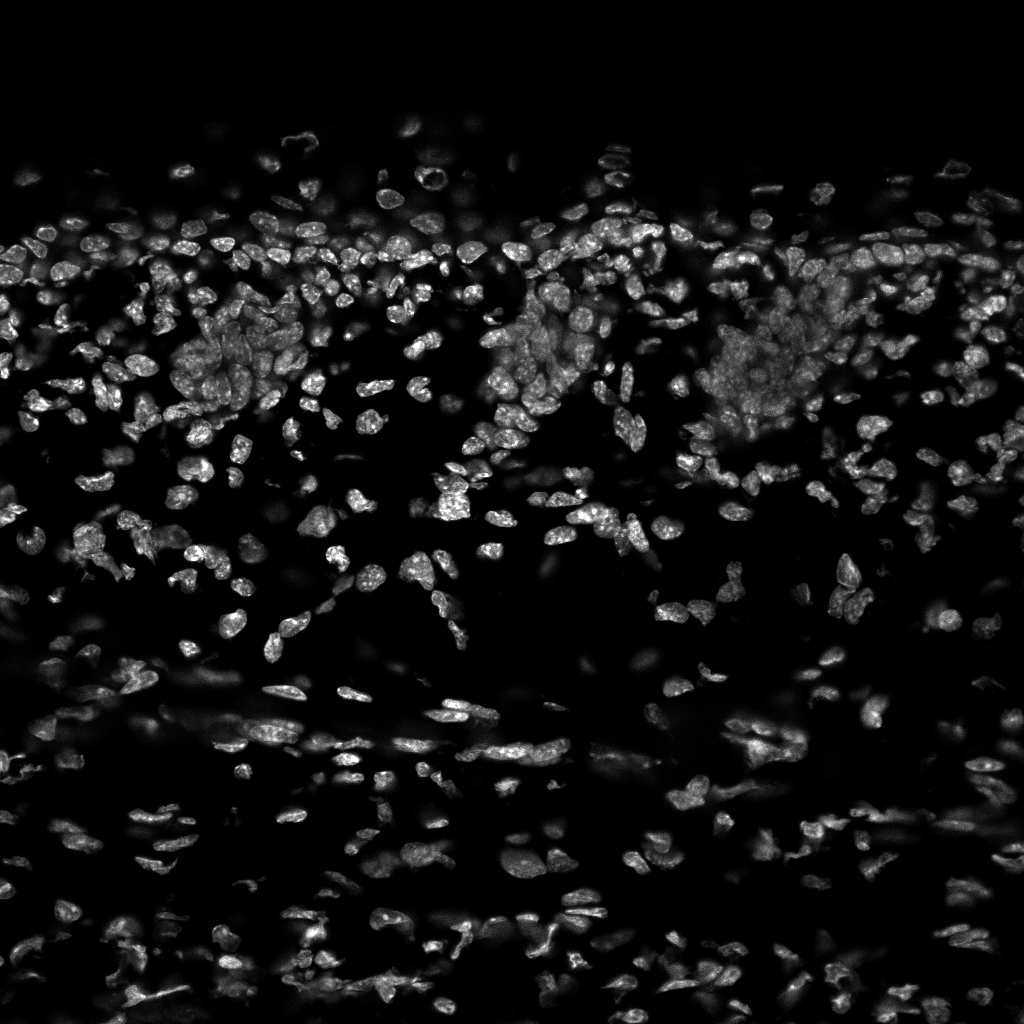

Supplement: Supplementary file 2 — Source Data Fig. 1 [file 44318_2024_39_MOESM2_ESM.zip › Figure 1/1A/E18.5 WT BETA1green GLUT1red 40x.tif]

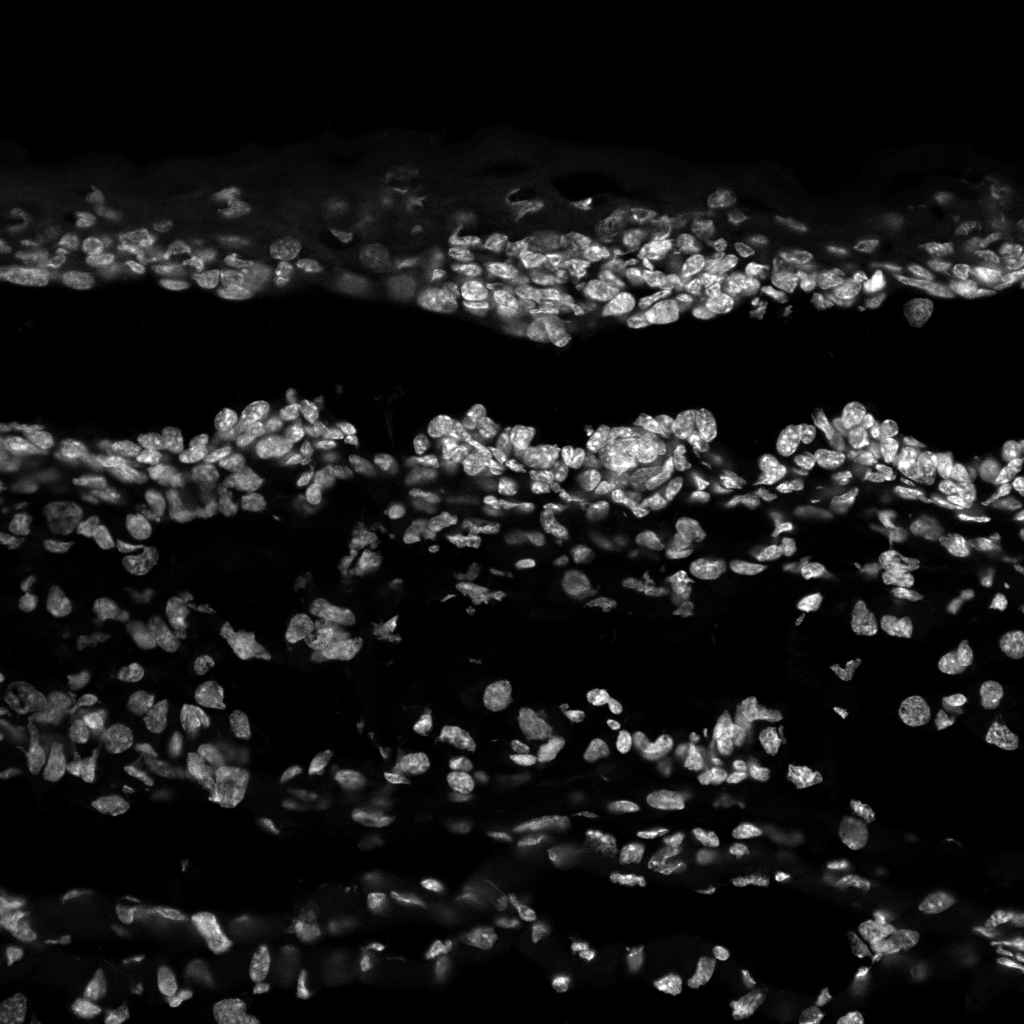

Supplement: Supplementary file 2 — Source Data Fig. 1 [file 44318_2024_39_MOESM2_ESM.zip › Figure 1/1A/E18.5 KO BETA1green GLUT1red 40x.tif]

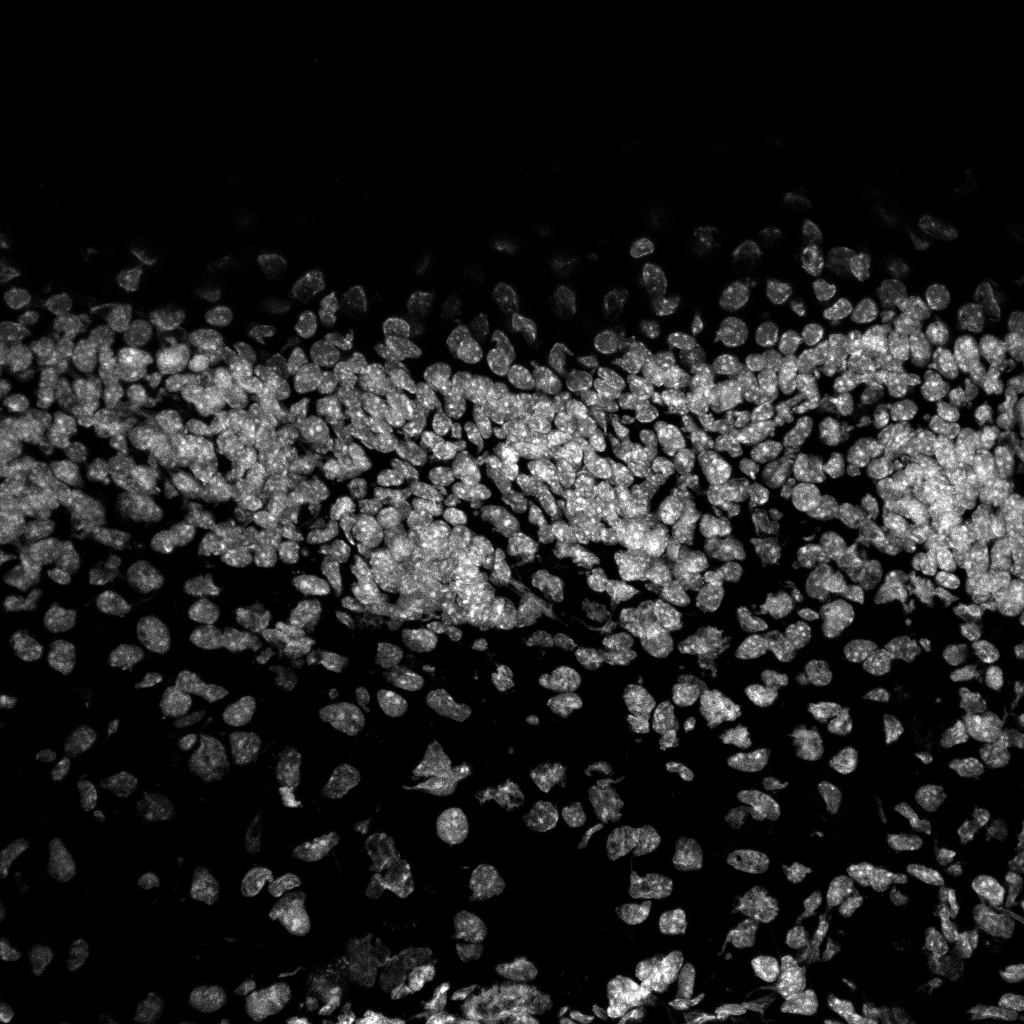

Supplement: Supplementary file 2 — Source Data Fig. 1 [file 44318_2024_39_MOESM2_ESM.zip › Figure 1/1I/E18.5 WT F480 green CSred 40x.tif]

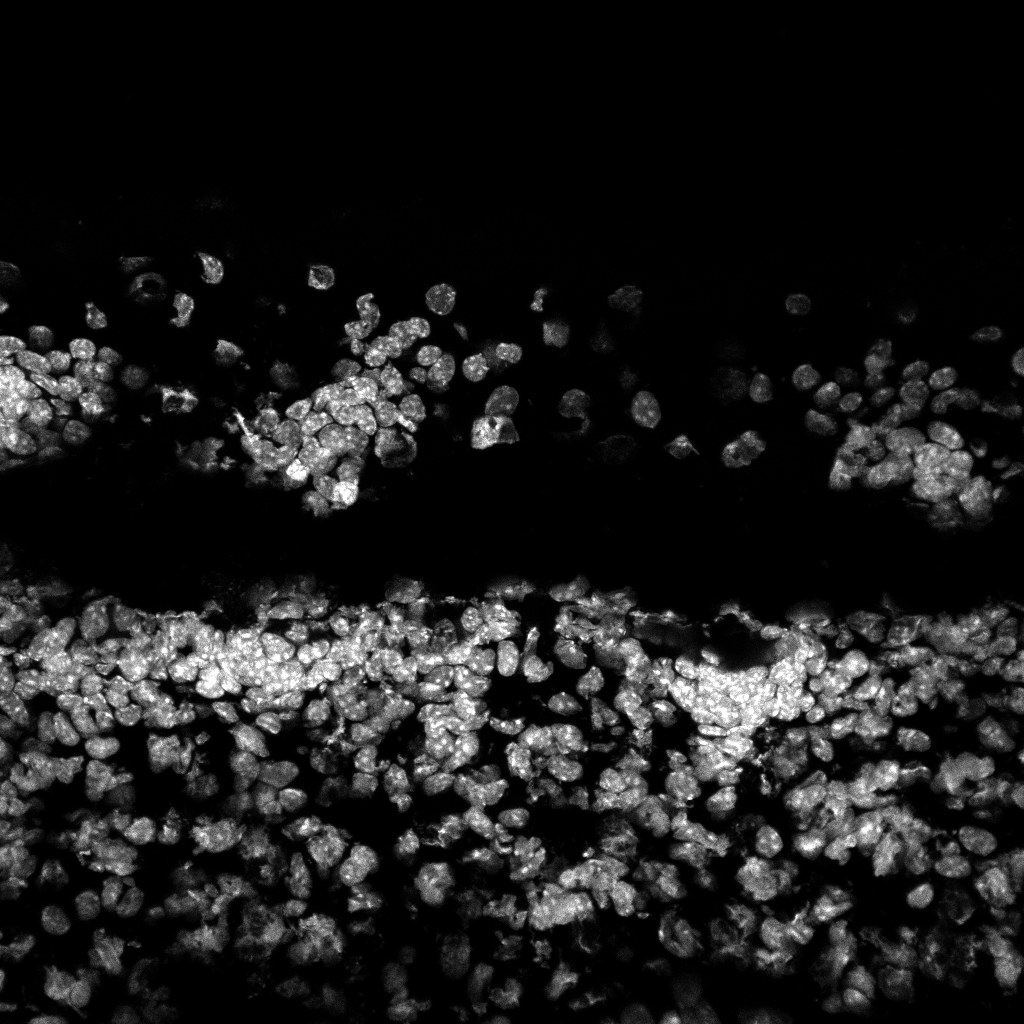

Supplement: Supplementary file 2 — Source Data Fig. 1 [file 44318_2024_39_MOESM2_ESM.zip › Figure 1/1I/E18.5 KO F480green CSred 40x.tif]

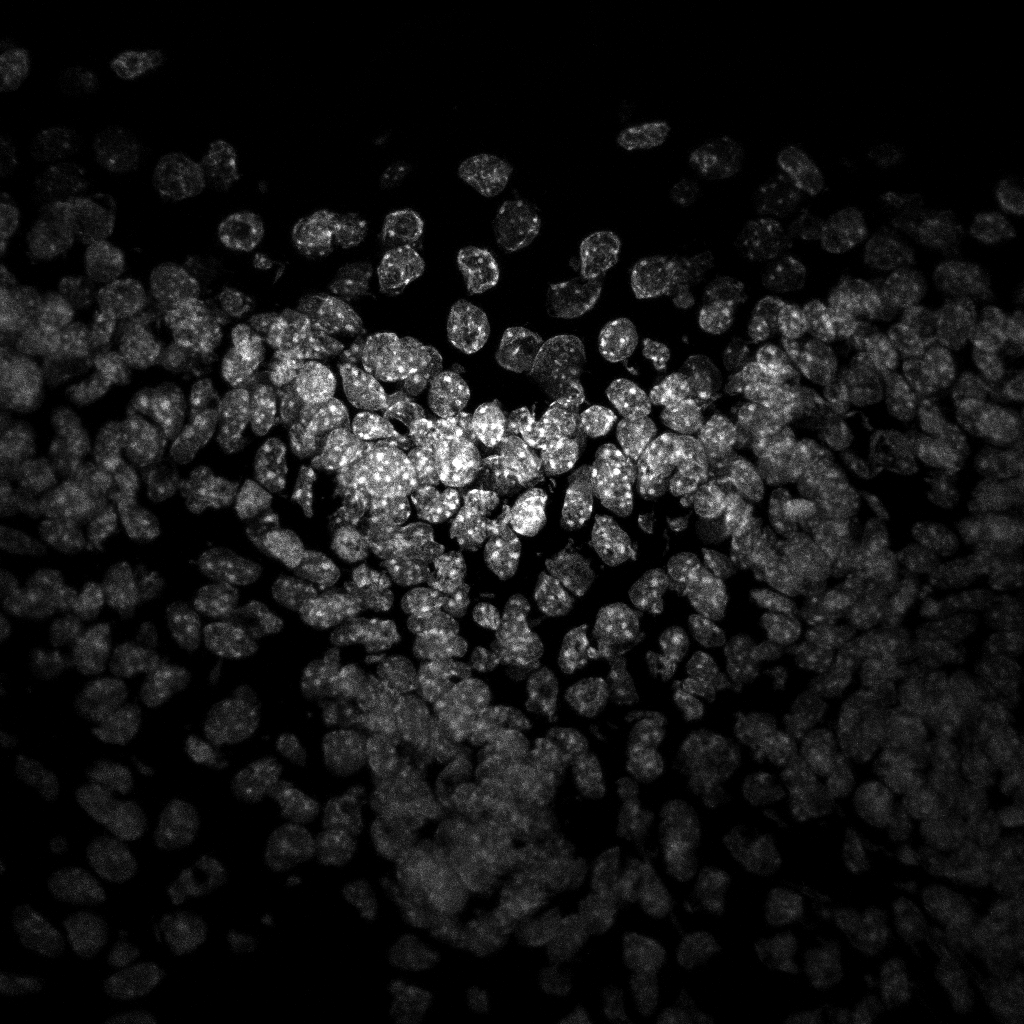

Supplement: Supplementary file 2 — Source Data Fig. 1 [file 44318_2024_39_MOESM2_ESM.zip › Figure 1/1B/E18.5 WT F480green LDHAred 60X.tif]

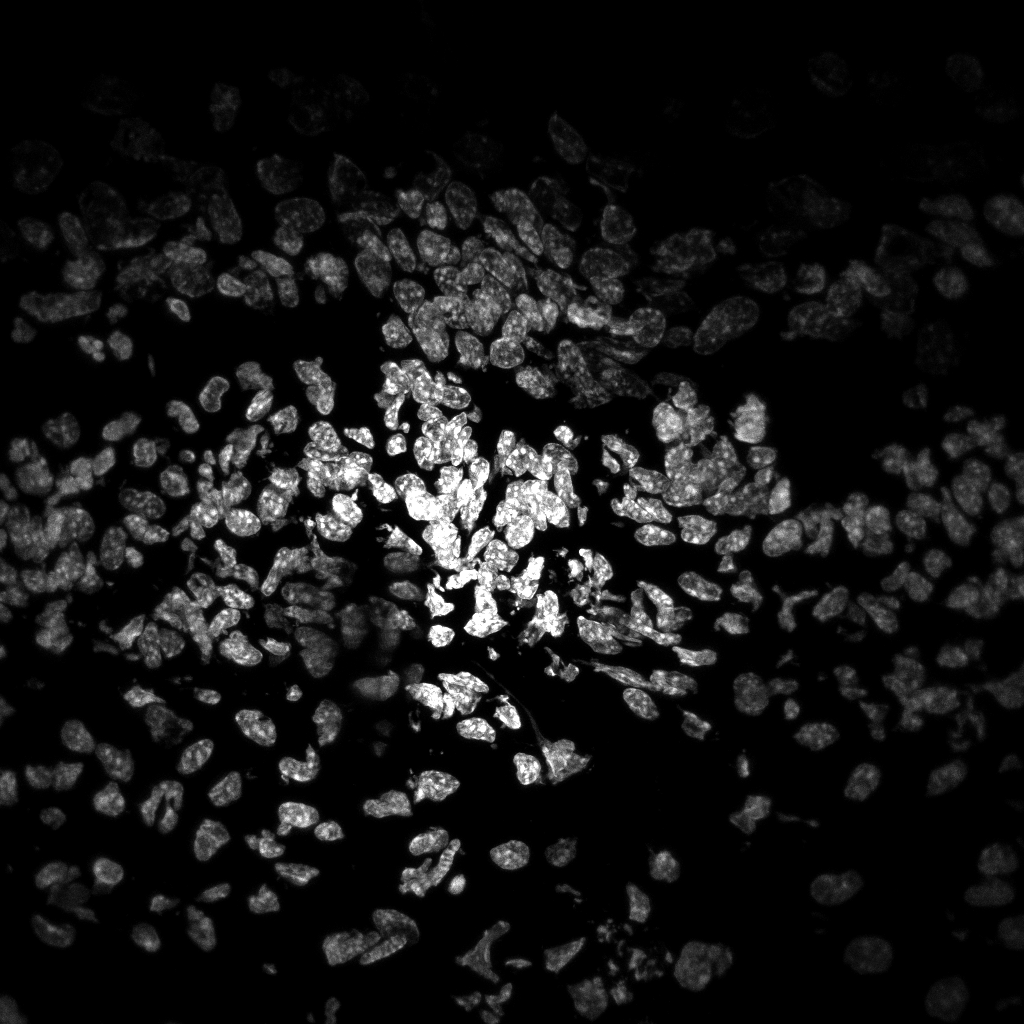

Supplement: Supplementary file 2 — Source Data Fig. 1 [file 44318_2024_39_MOESM2_ESM.zip › Figure 1/1B/E18.5 KO F480green LDHAred 60X.tif]

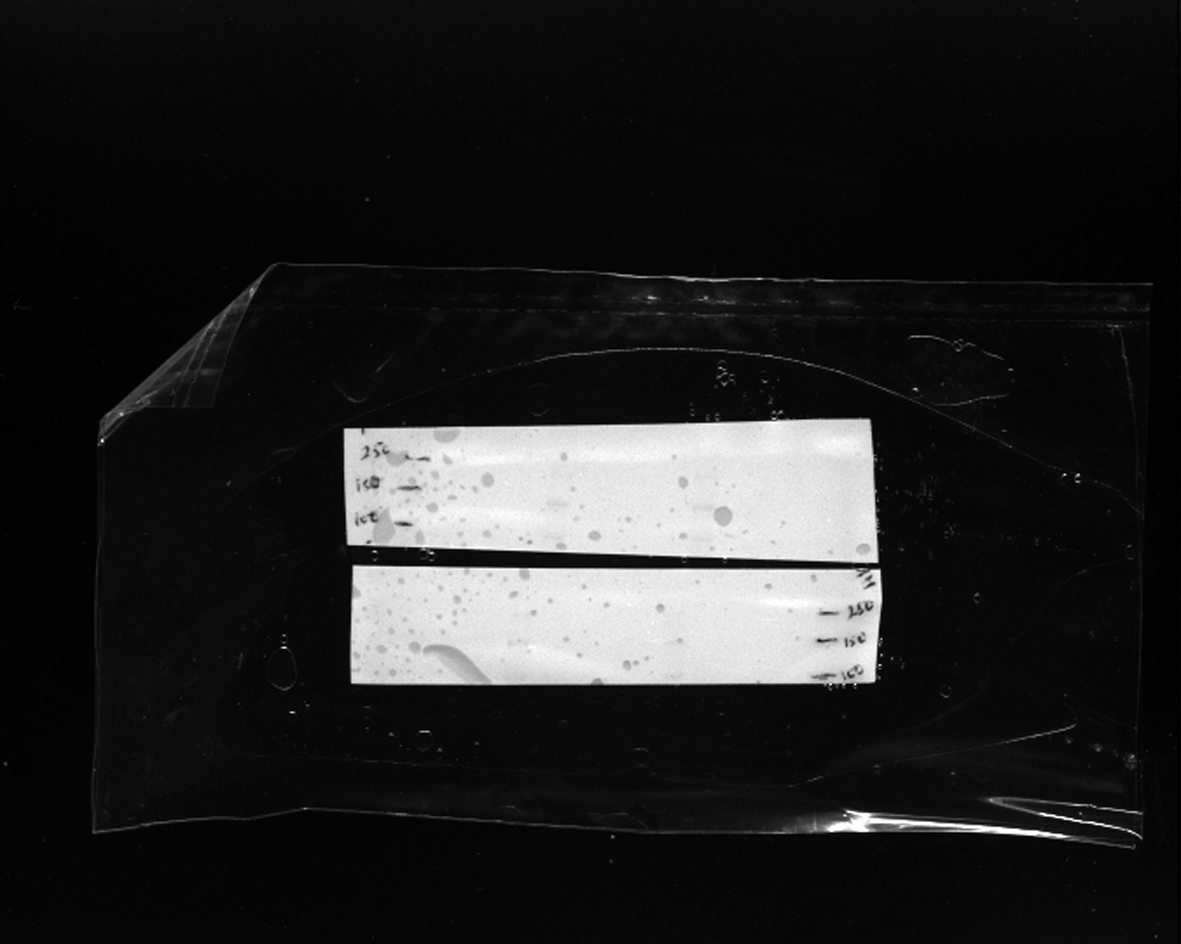

Supplement: Supplementary file 2 — Source Data Fig. 1 [file 44318_2024_39_MOESM2_ESM.zip › Figure 1/1D/HK_2_UPPER_EPI_BELOW_DER/HK_2_UPPER_EPI_BELOW_DER_(Membrane).tif]

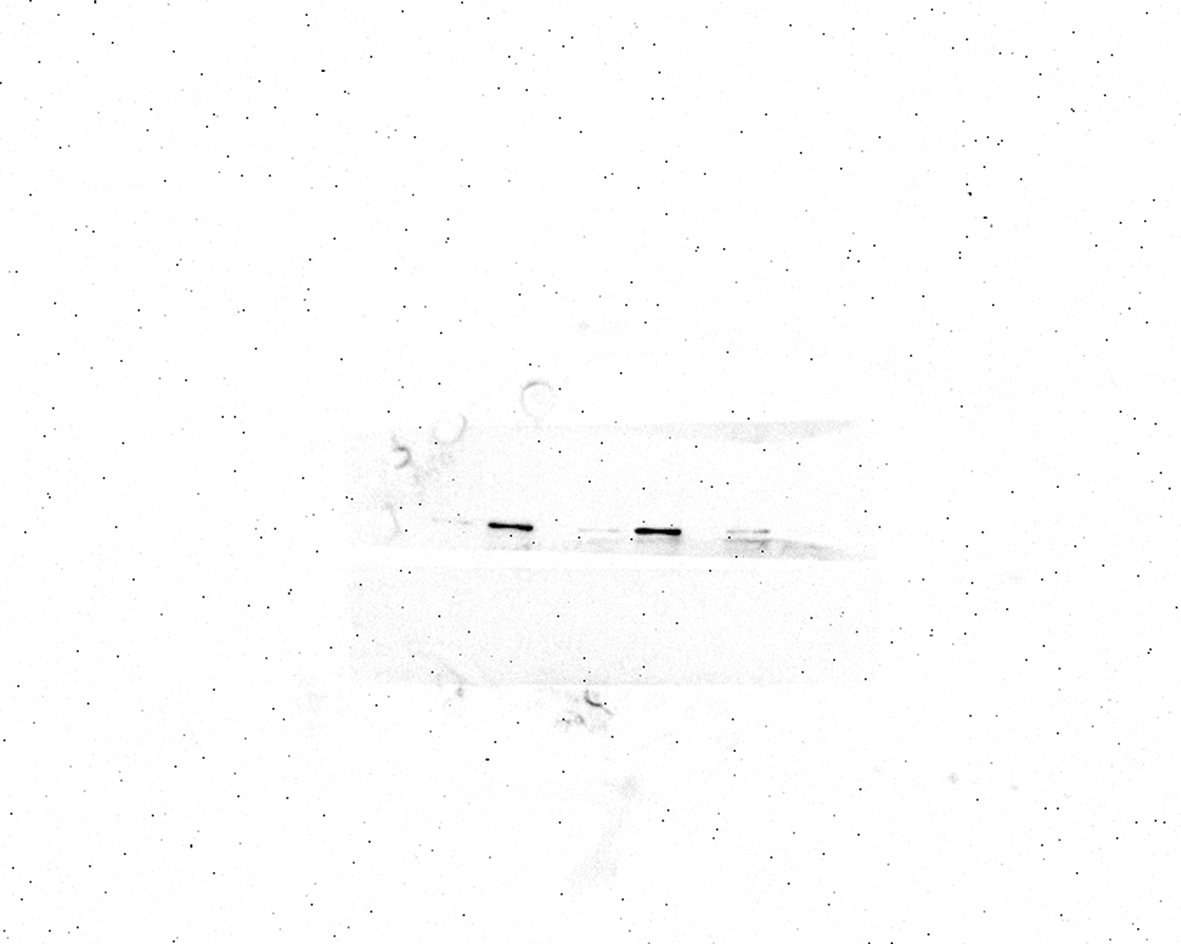

Supplement: Supplementary file 2 — Source Data Fig. 1 [file 44318_2024_39_MOESM2_ESM.zip › Figure 1/1D/HK_2_UPPER_EPI_BELOW_DER/HK_2_UPPER_EPI_BELOW_DER_(Chemi).tif]

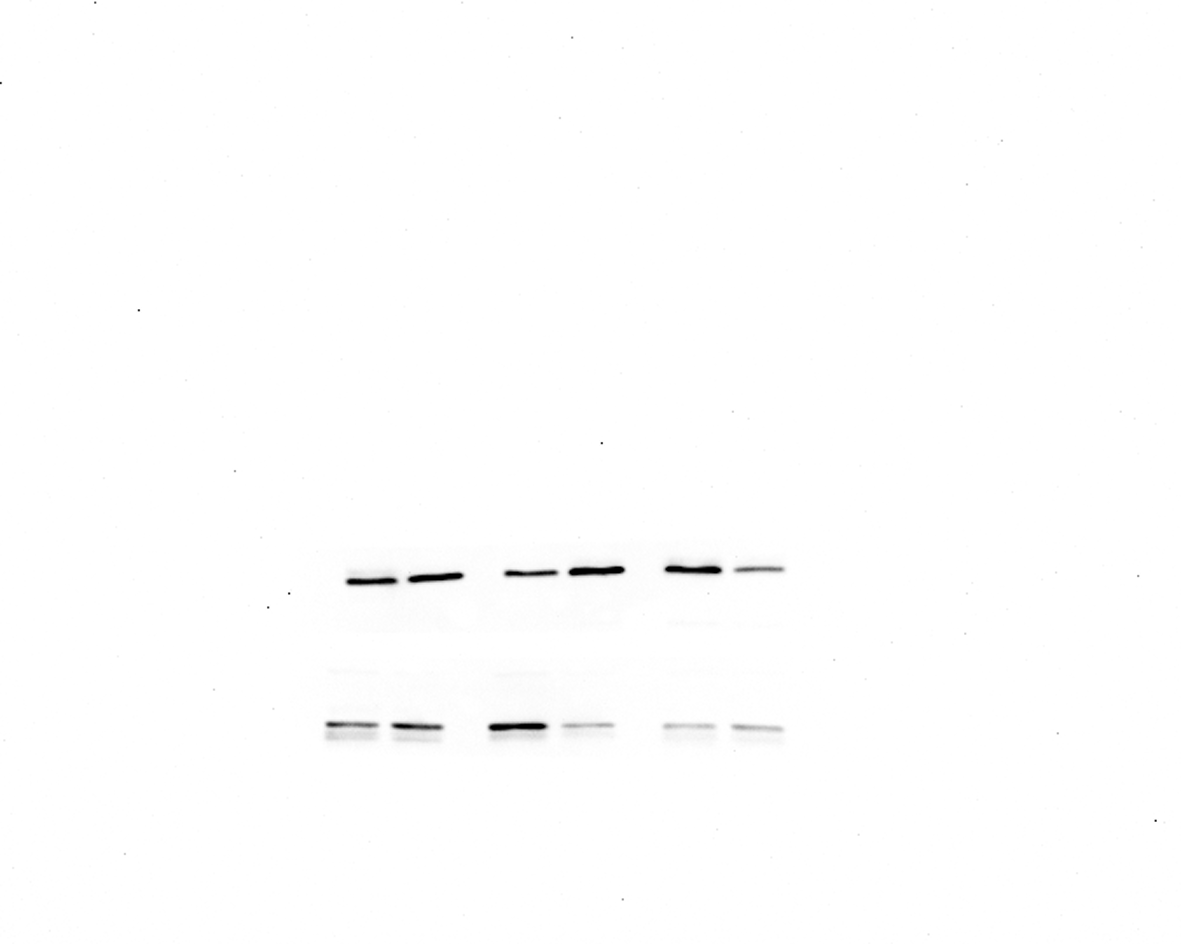

Supplement: Supplementary file 2 — Source Data Fig. 1 [file 44318_2024_39_MOESM2_ESM.zip › Figure 1/1D/a tubulin_top der_bottom epi 12sec exp/a tubulin_top der_bottom epi 12sec exp_Chemi).tif]

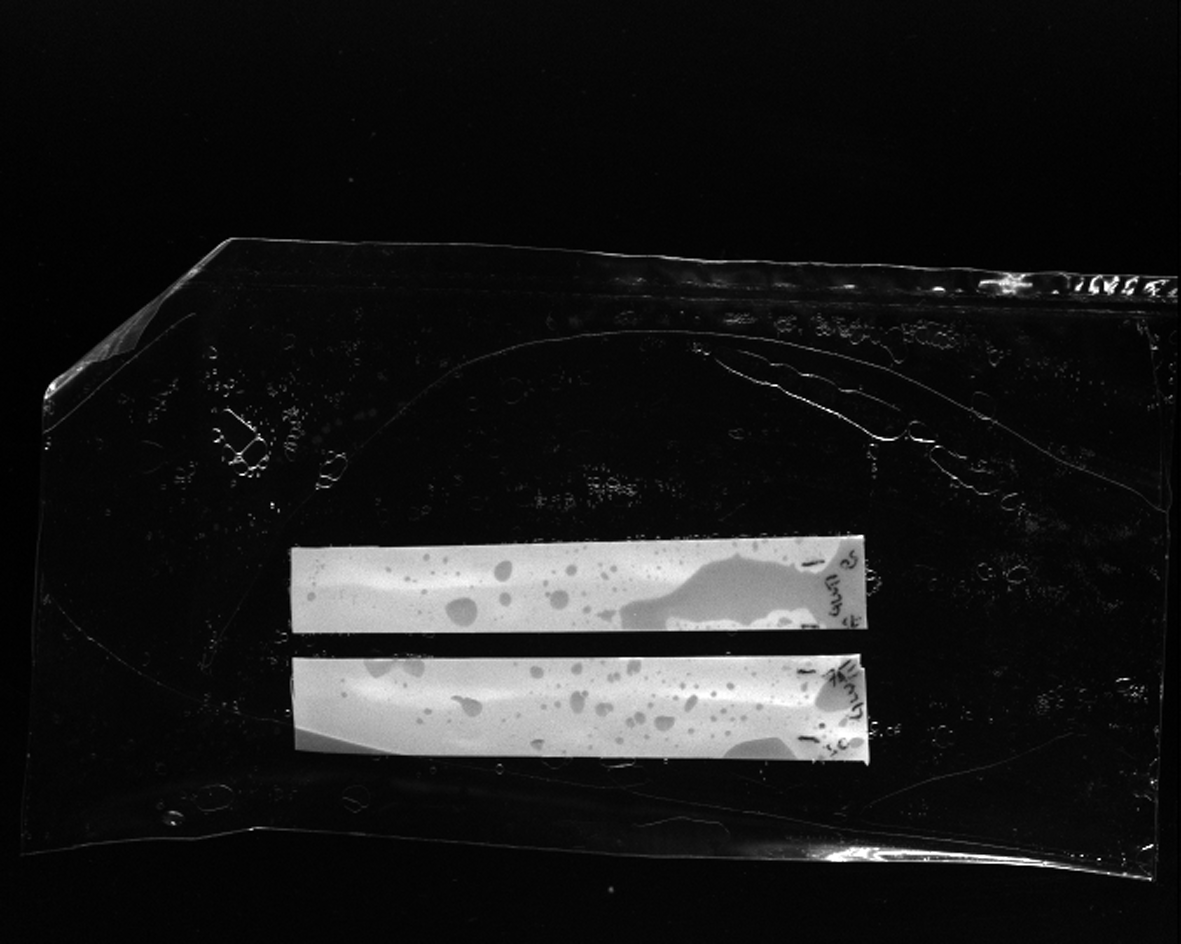

Supplement: Supplementary file 2 — Source Data Fig. 1 [file 44318_2024_39_MOESM2_ESM.zip › Figure 1/1D/a tubulin_top der_bottom epi 12sec exp/a tubulin_top der_bottom epi 12sec exp_(Membrane).tif]

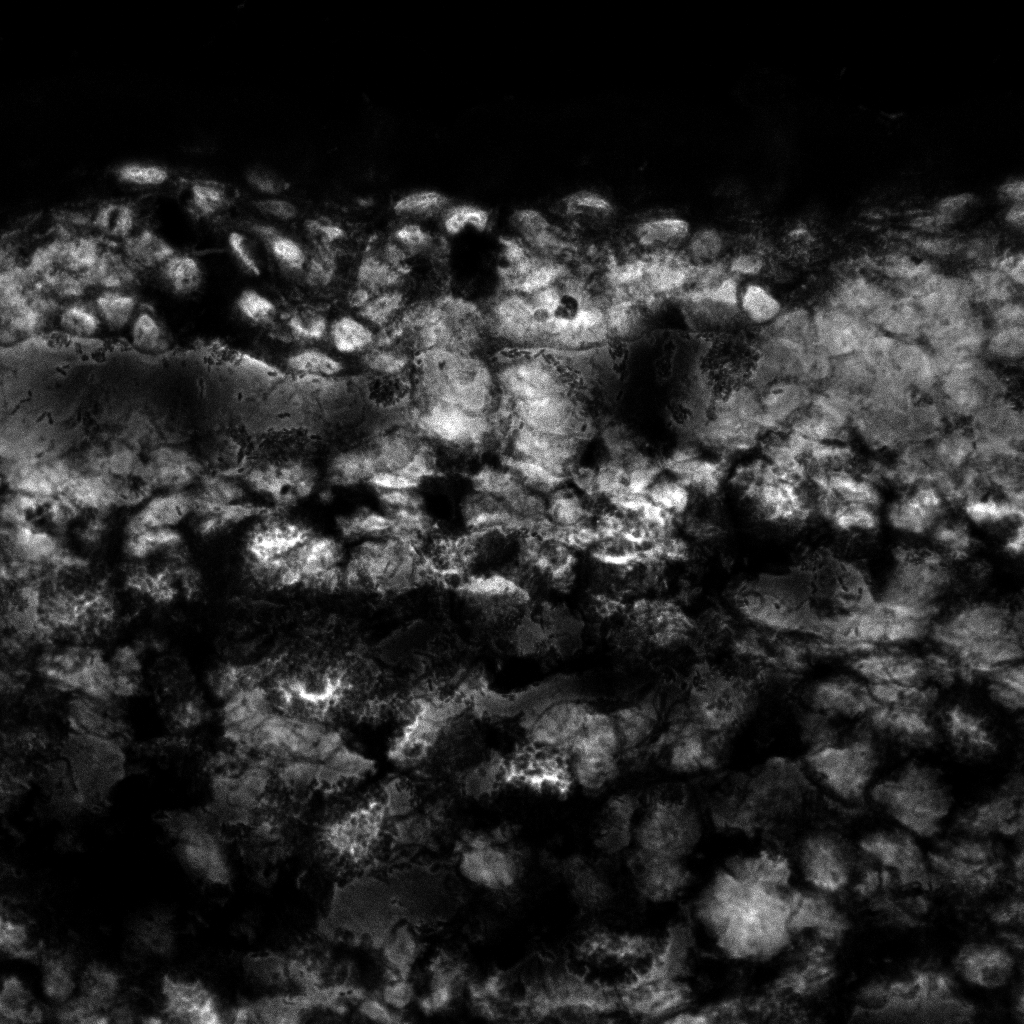

Supplement: Supplementary file 3 — Source Data Fig. 2 [file 44318_2024_39_MOESM3_ESM.zip › Figure 2/2B/E18.5 KO HIF1Ared ECADgreen 40x 1.4x.tif]

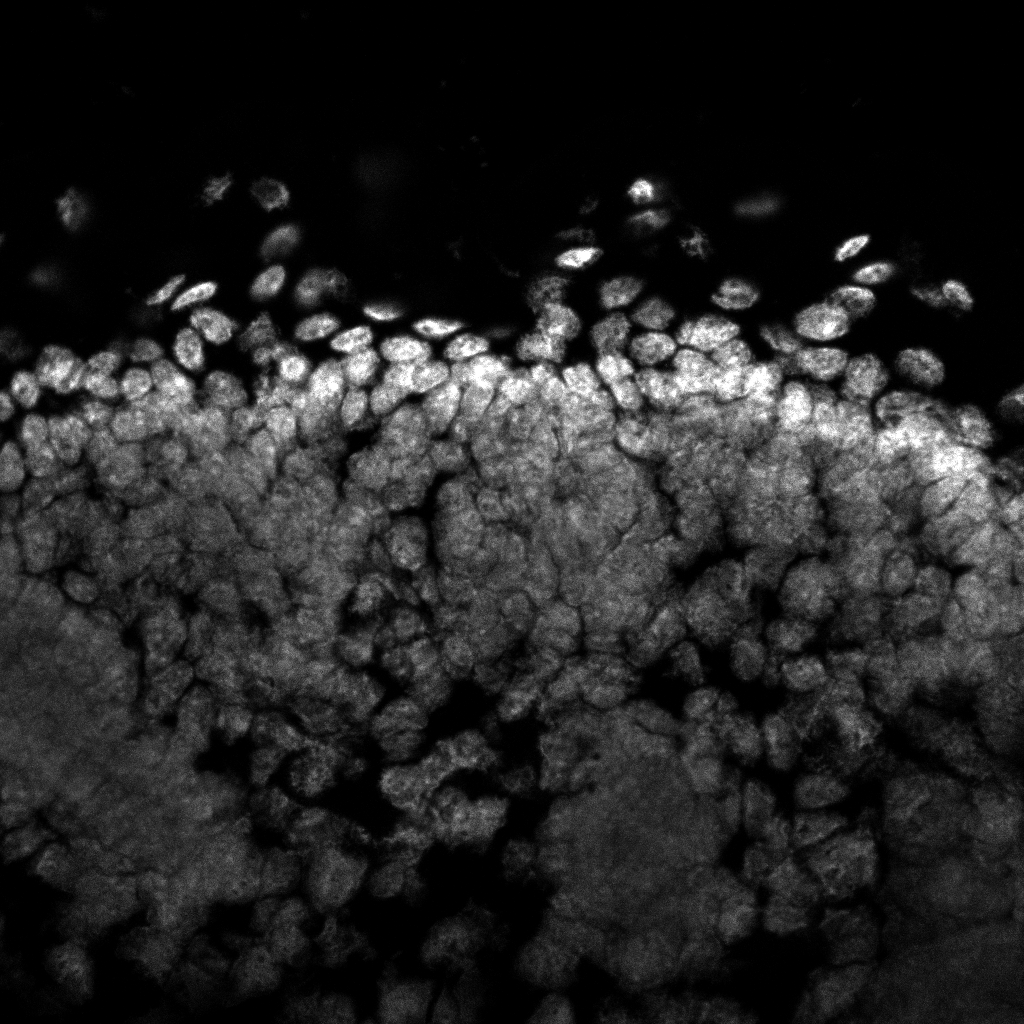

Supplement: Supplementary file 3 — Source Data Fig. 2 [file 44318_2024_39_MOESM3_ESM.zip › Figure 2/2B/E18.5 WT HIF1Ared ECADgreen 40x 1.4x.tif]

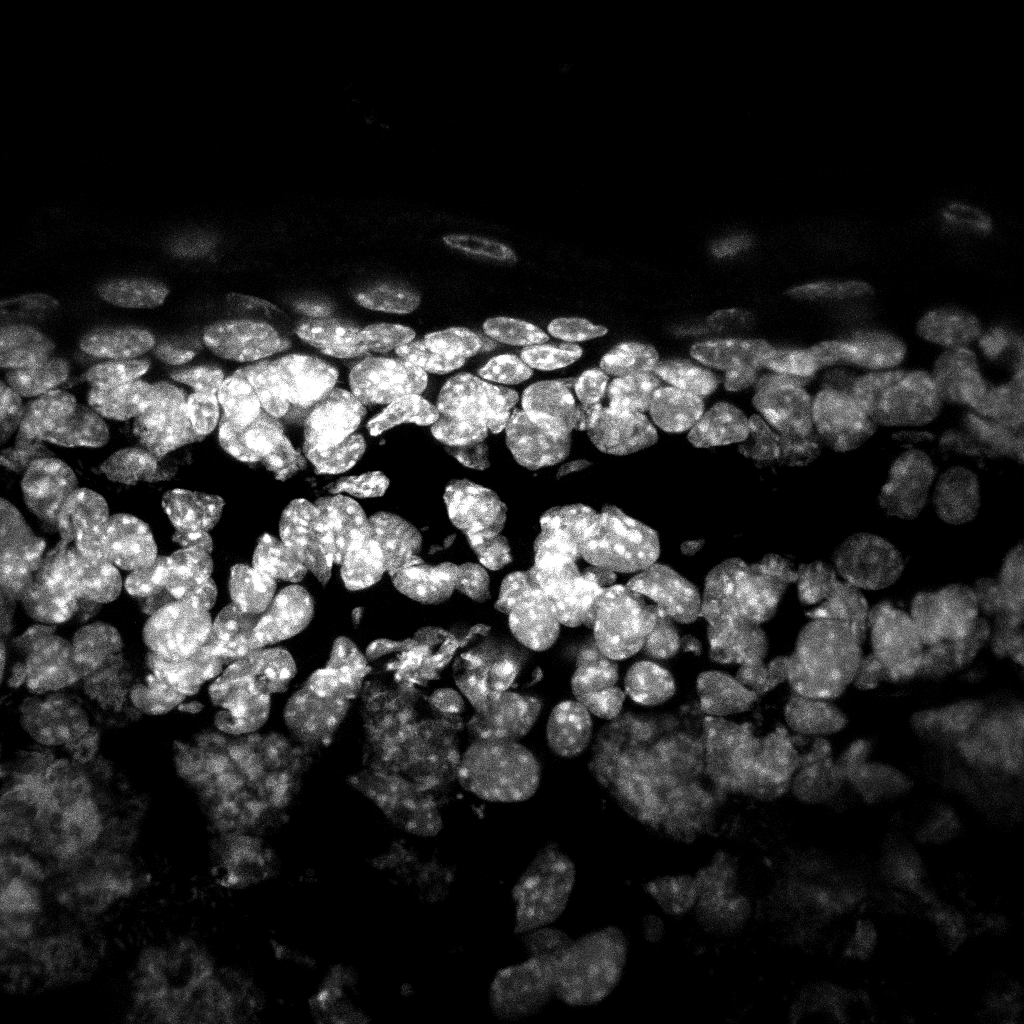

Supplement: Supplementary file 3 — Source Data Fig. 2 [file 44318_2024_39_MOESM3_ESM.zip › Figure 2/2H/LDHa F480 staining/E18.5 DMSO KO F480green LDHAred 40X 2.2X.tif]

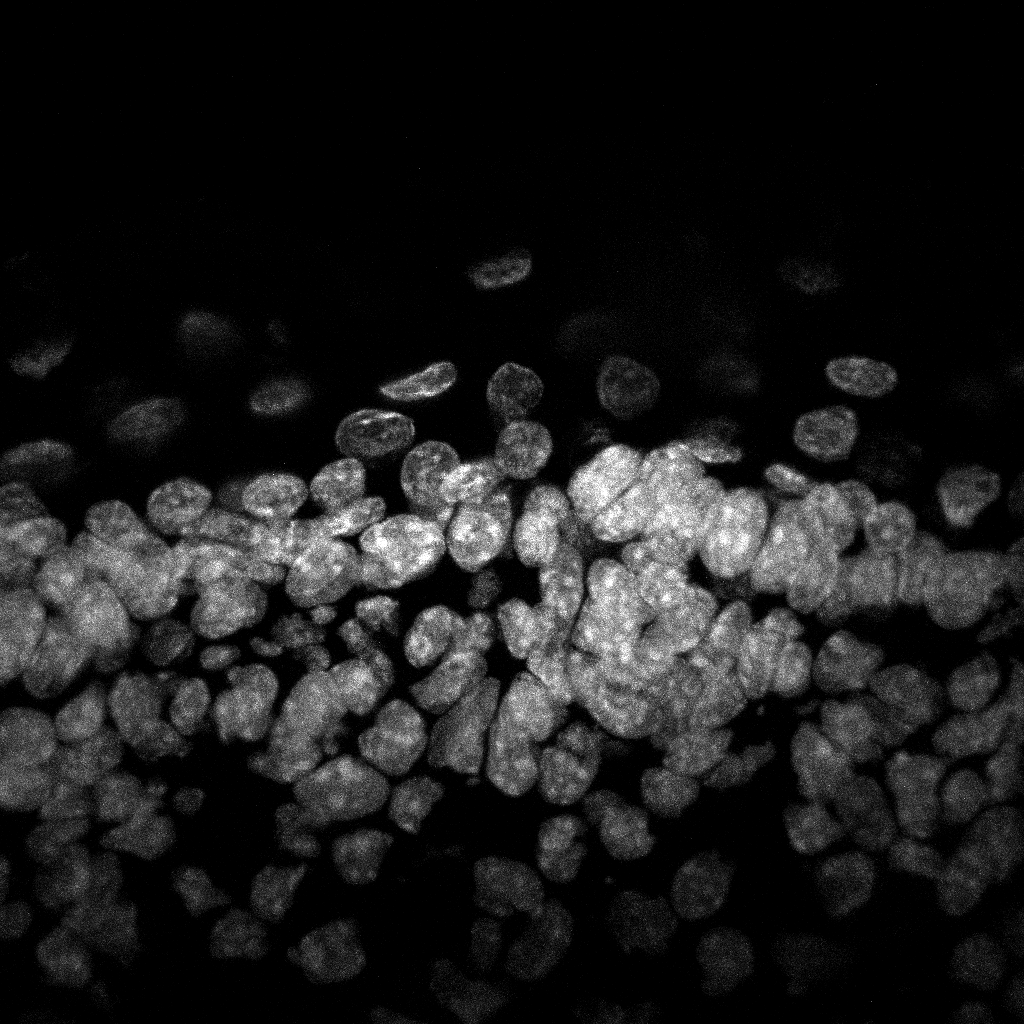

Supplement: Supplementary file 3 — Source Data Fig. 2 [file 44318_2024_39_MOESM3_ESM.zip › Figure 2/2H/LDHa F480 staining/E18.5 CHETOMIN F480green LDHAred 40X 2.2X.tif]

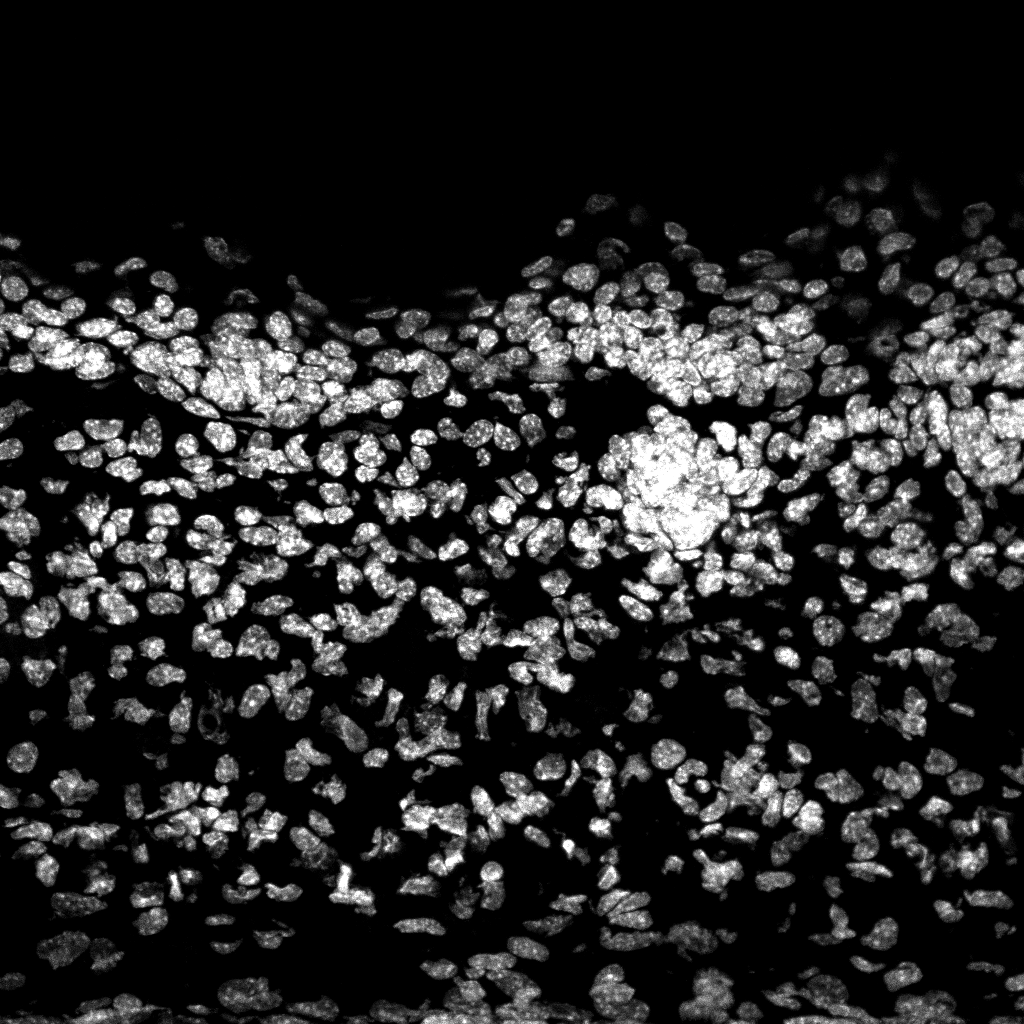

Supplement: Supplementary file 3 — Source Data Fig. 2 [file 44318_2024_39_MOESM3_ESM.zip › Figure 2/2H/GLUT1 F480 staining/E18.5 DMSO KO F480green GLUT1red 40X.tif]

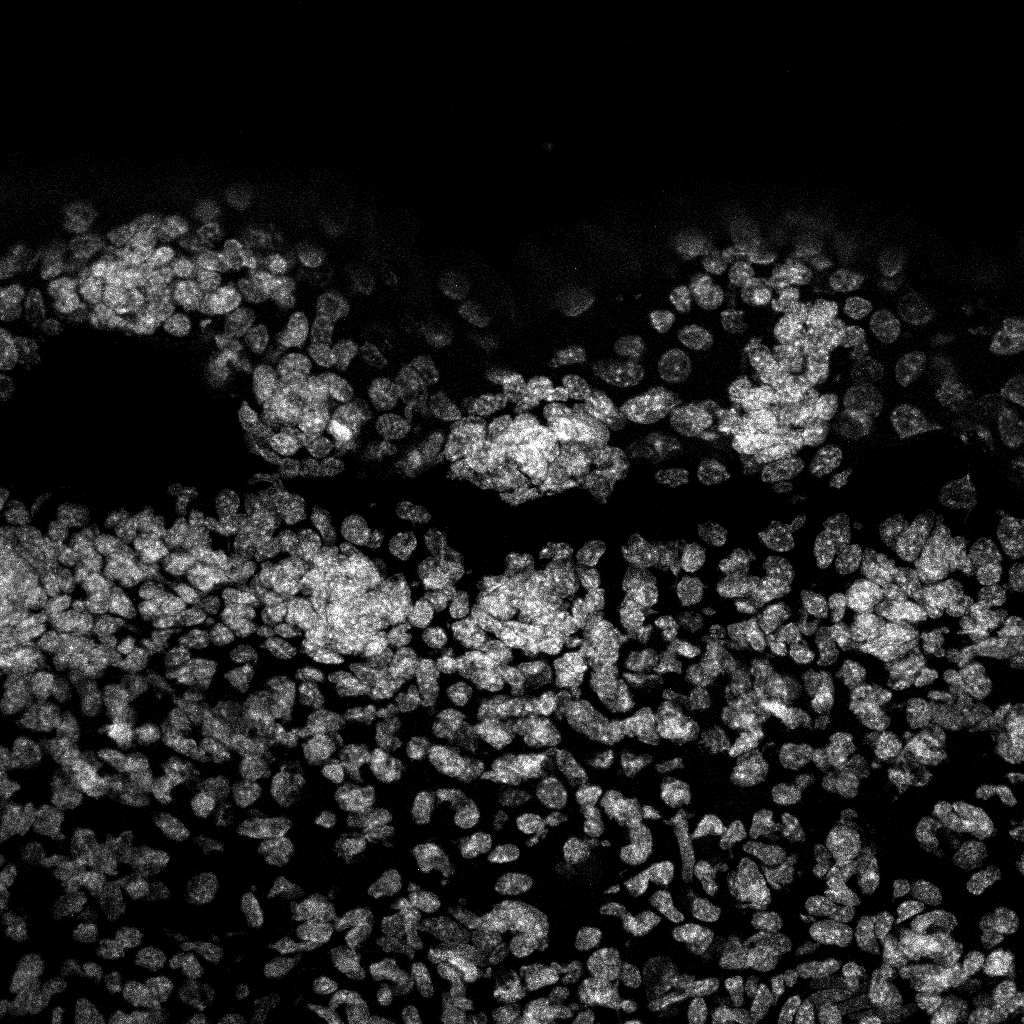

Supplement: Supplementary file 3 — Source Data Fig. 2 [file 44318_2024_39_MOESM3_ESM.zip › Figure 2/2H/GLUT1 F480 staining/E18.5 CHETOMIN KO F480green GLUT1red 40X1.tif]

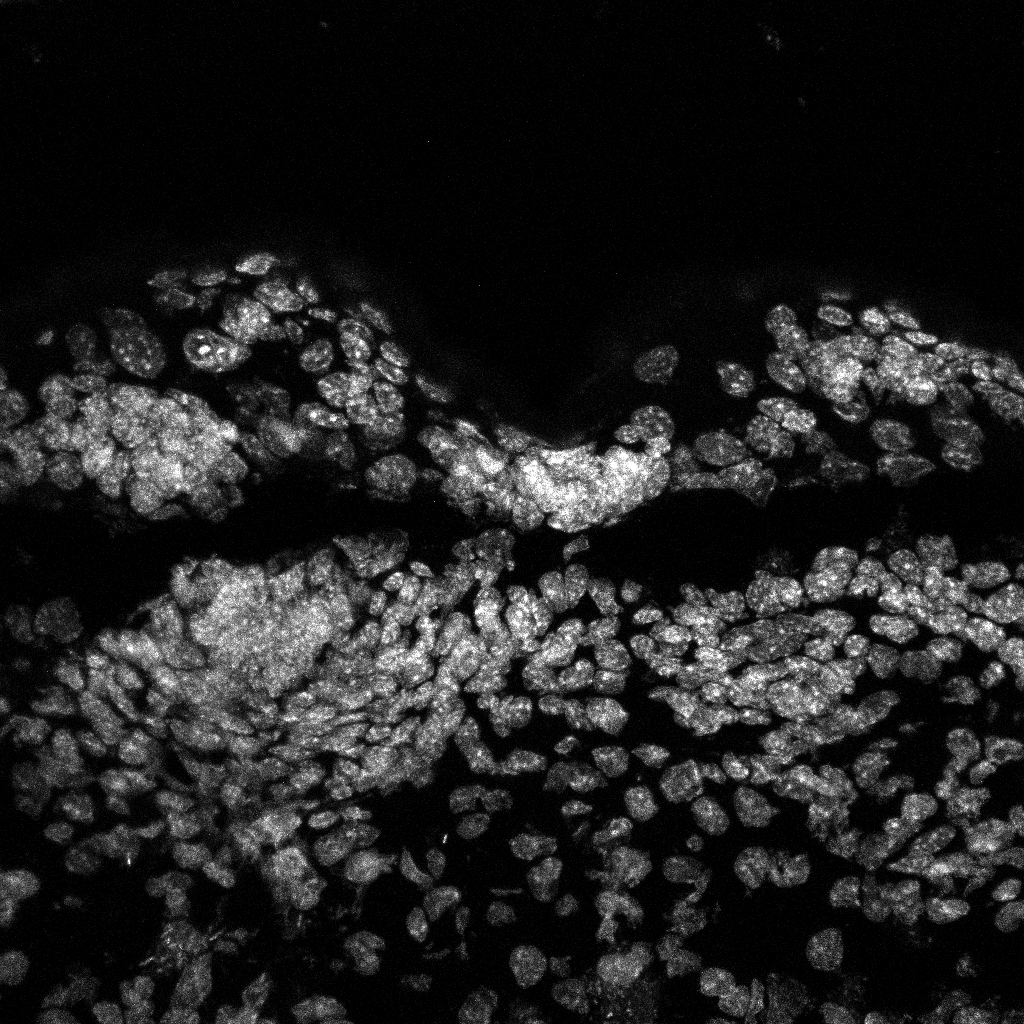

Supplement: Supplementary file 3 — Source Data Fig. 2 [file 44318_2024_39_MOESM3_ESM.zip › Figure 2/2F/KRT14 ECAD staining/E18.5 KO CHETOMIN ECADgreen KRT14red 40x 1.2x.tif]

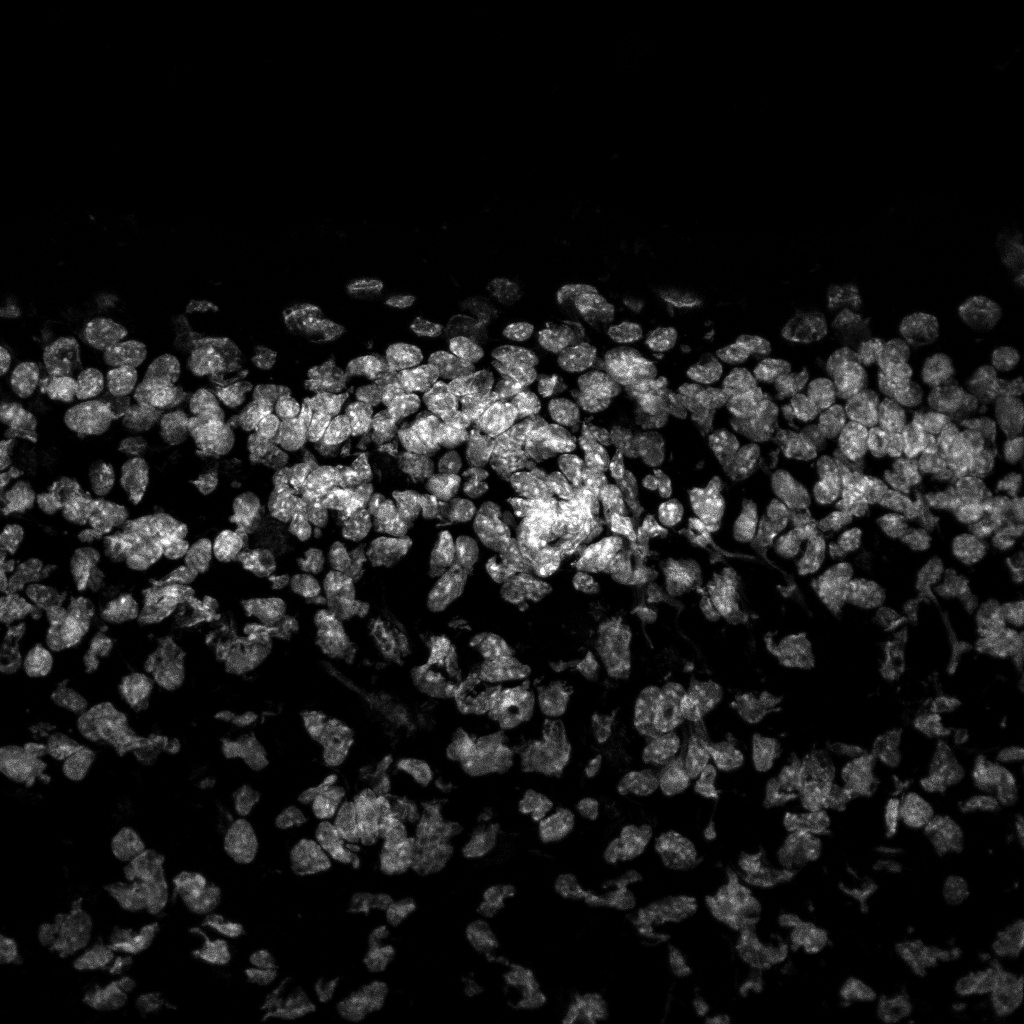

Supplement: Supplementary file 3 — Source Data Fig. 2 [file 44318_2024_39_MOESM3_ESM.zip › Figure 2/2F/KRT14 ECAD staining/E18.5 KO DMSO ECADgreen KRT14red 40x 1.2x.tif]

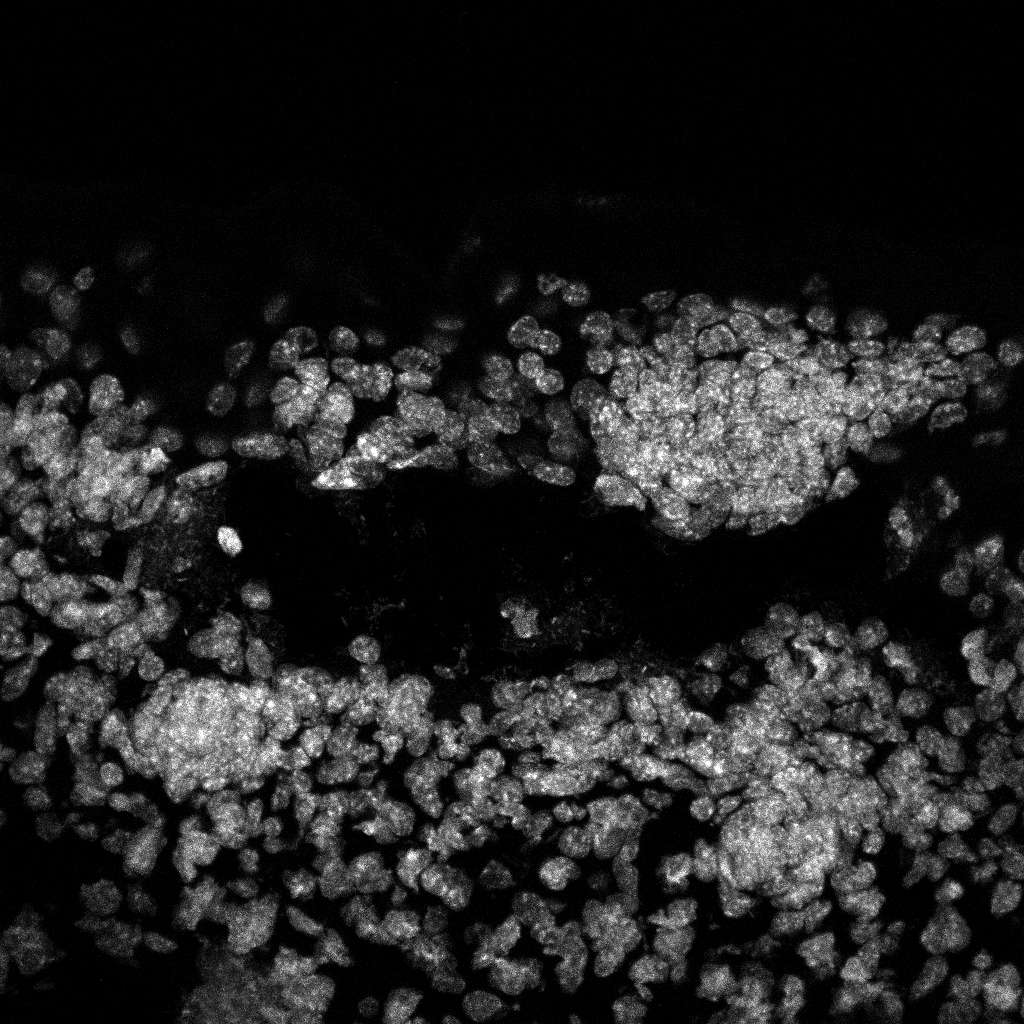

Supplement: Supplementary file 3 — Source Data Fig. 2 [file 44318_2024_39_MOESM3_ESM.zip › Figure 2/2F/COX2 ECAD staining/E18.5 KO DMSO ECADgreen COX2red 40x 1.2x.tif]

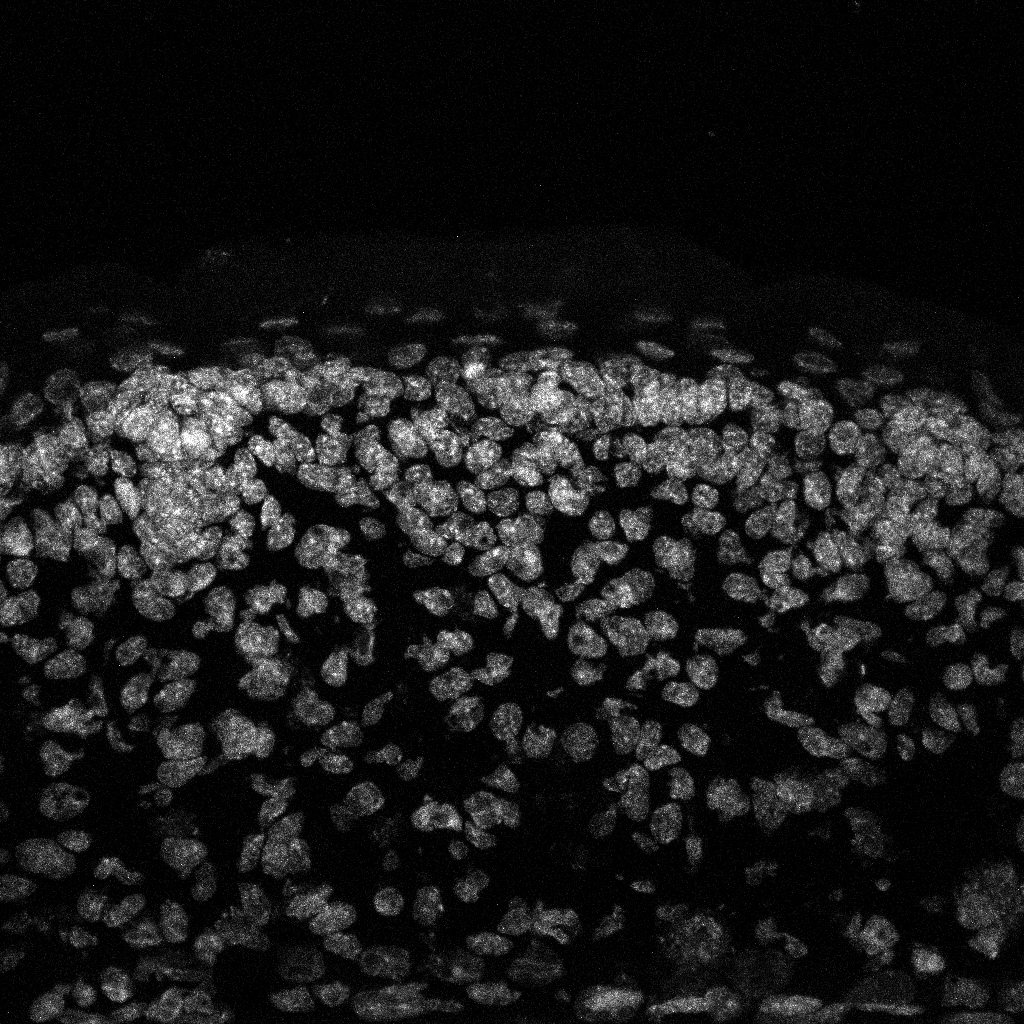

Supplement: Supplementary file 3 — Source Data Fig. 2 [file 44318_2024_39_MOESM3_ESM.zip › Figure 2/2F/COX2 ECAD staining/E18.5 KO CHETOMIN ECADgreen COX2red 40x 1.2x.tif]

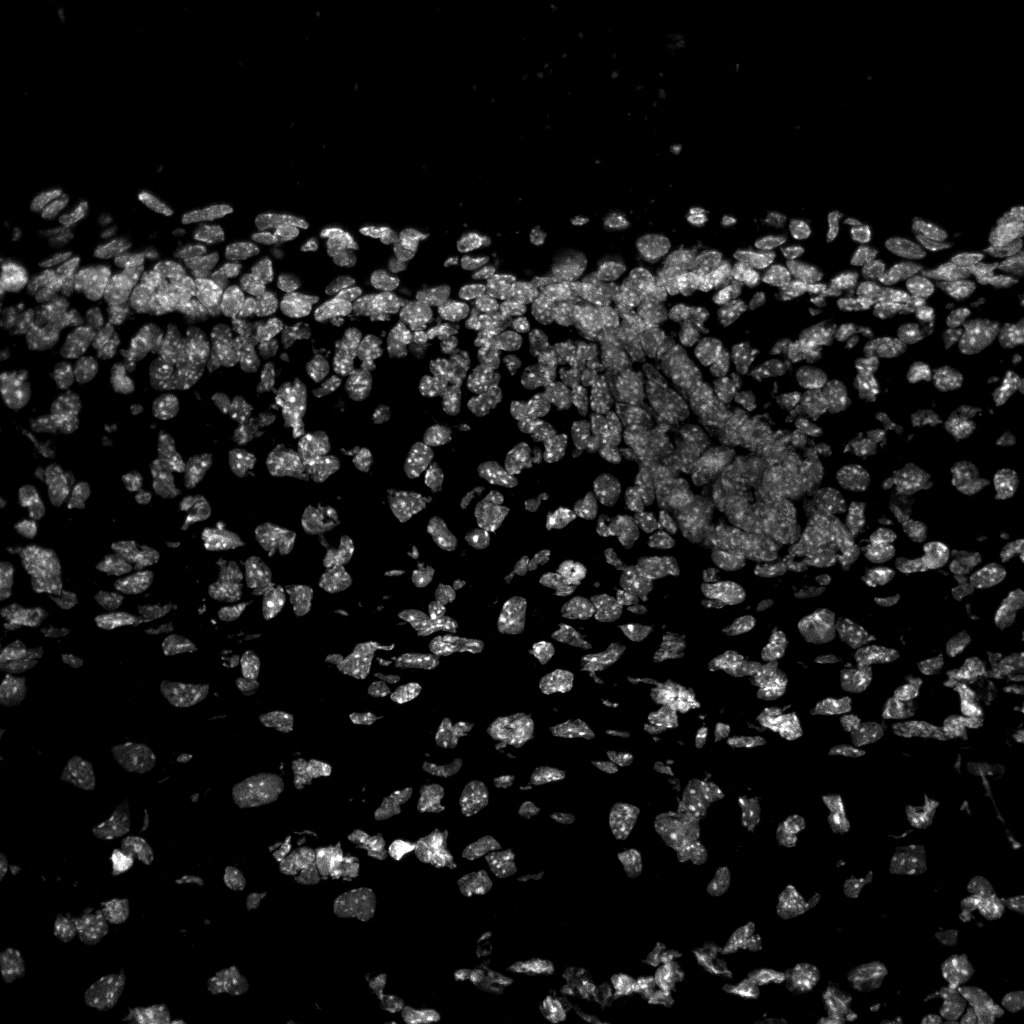

Supplement: Supplementary file 4 — Source Data Fig. 3 [file 44318_2024_39_MOESM4_ESM.zip › Figure 3/3B/E16.5 OHDG F480 Staining/E16.5 WT F480green OHDGred 40x.tif]

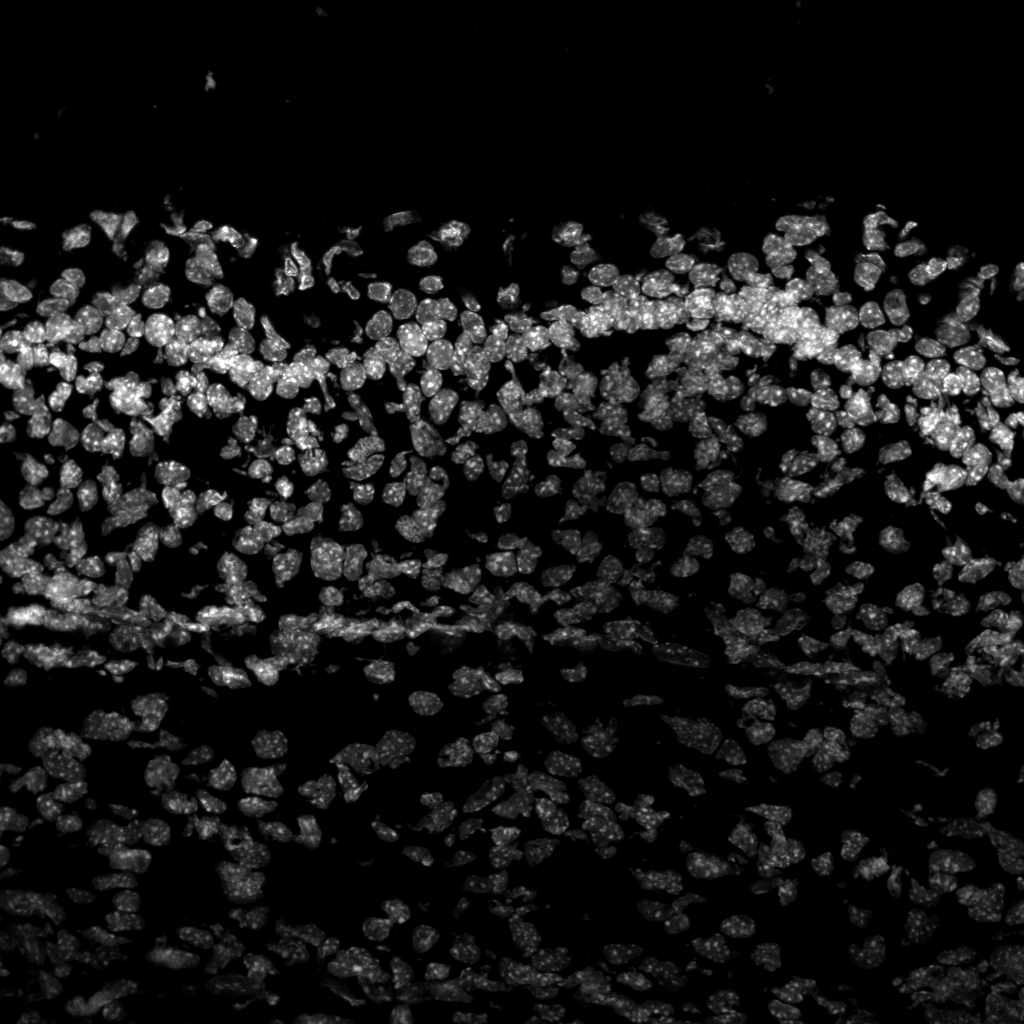

Supplement: Supplementary file 4 — Source Data Fig. 3 [file 44318_2024_39_MOESM4_ESM.zip › Figure 3/3B/E16.5 OHDG F480 Staining/E16.5 KO F480green OHDGred 40x.tif]

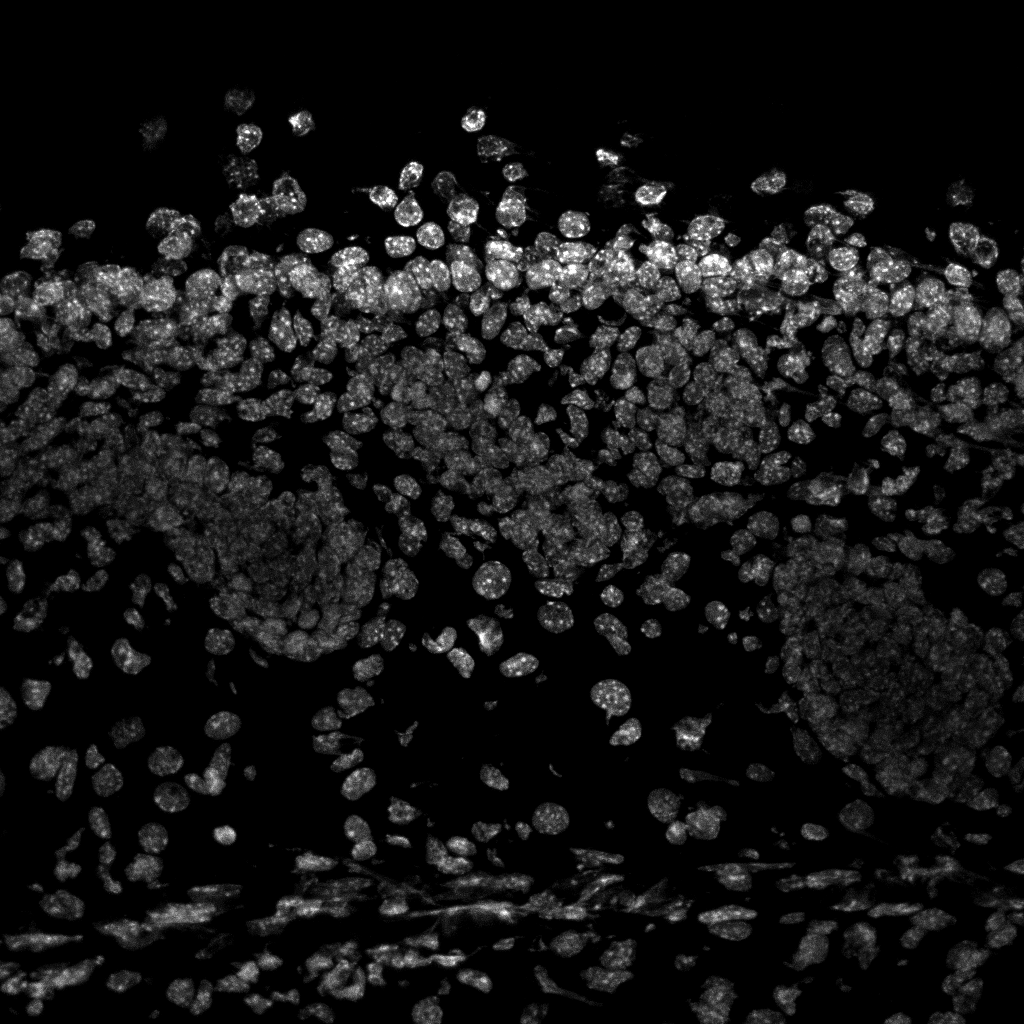

Supplement: Supplementary file 4 — Source Data Fig. 3 [file 44318_2024_39_MOESM4_ESM.zip › Figure 3/3B/E18.5 OHDG F480 Staining/E18.5 WT F480green ROSred 40X.tif]

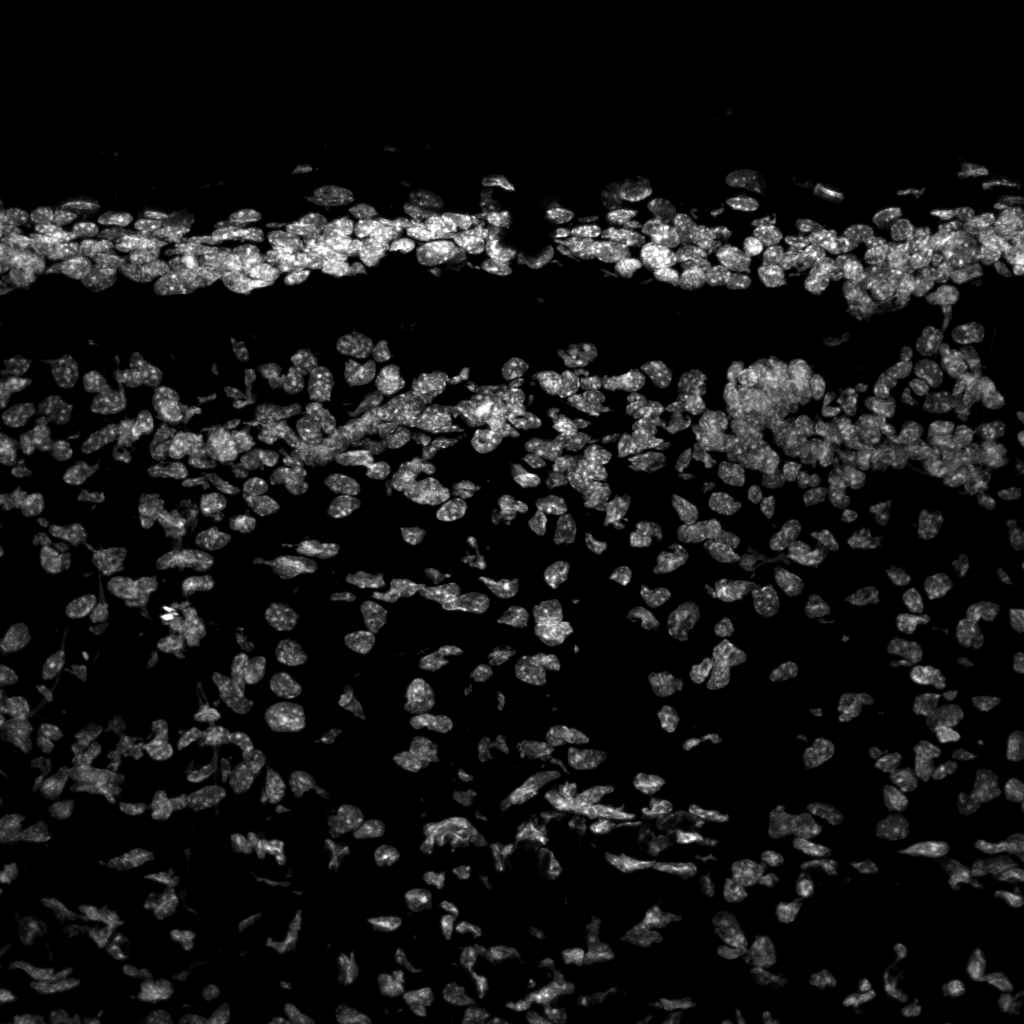

Supplement: Supplementary file 4 — Source Data Fig. 3 [file 44318_2024_39_MOESM4_ESM.zip › Figure 3/3B/E18.5 OHDG F480 Staining/E18.5 KO F480green ROSred 40X.tif]

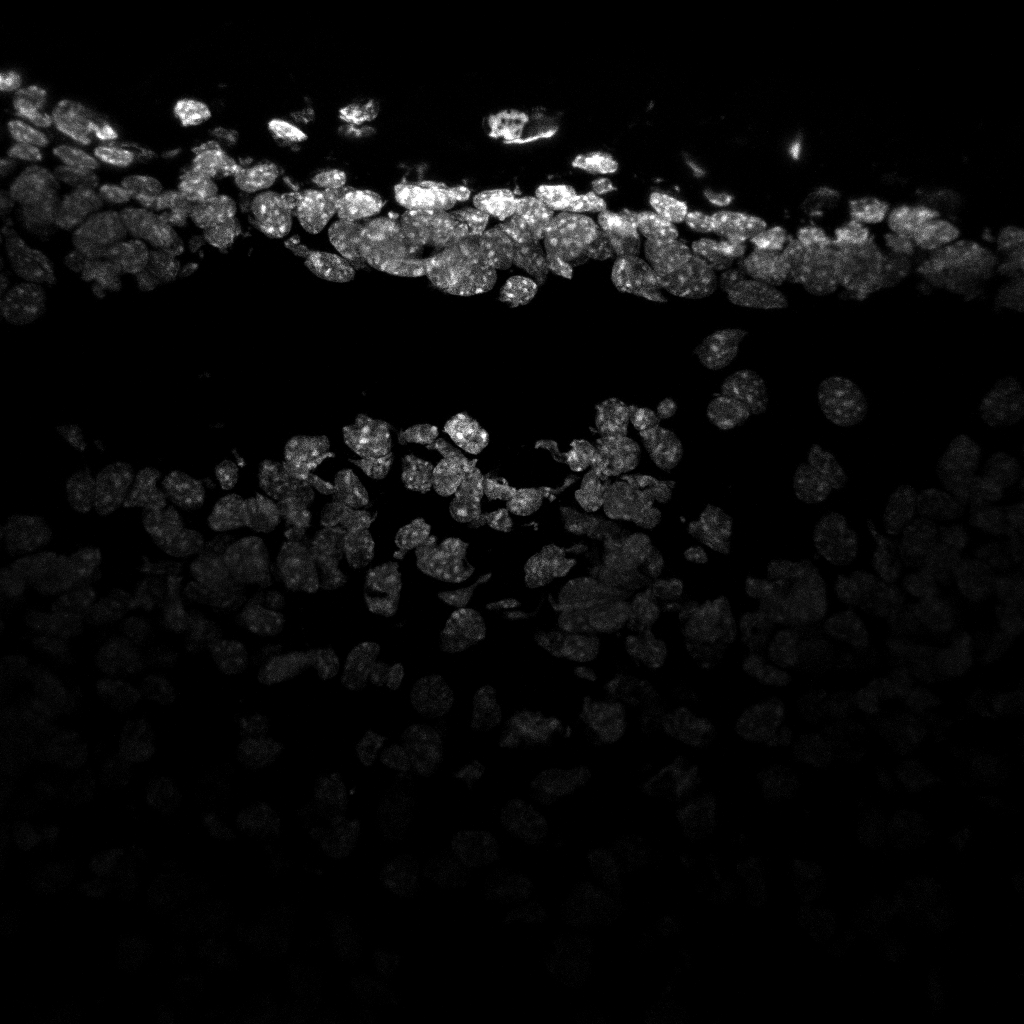

Supplement: Supplementary file 4 — Source Data Fig. 3 [file 44318_2024_39_MOESM4_ESM.zip › Figure 3/3H/F480 LDHA staining/E18.5 KO NAC F480green LDHAred 40X.tif]

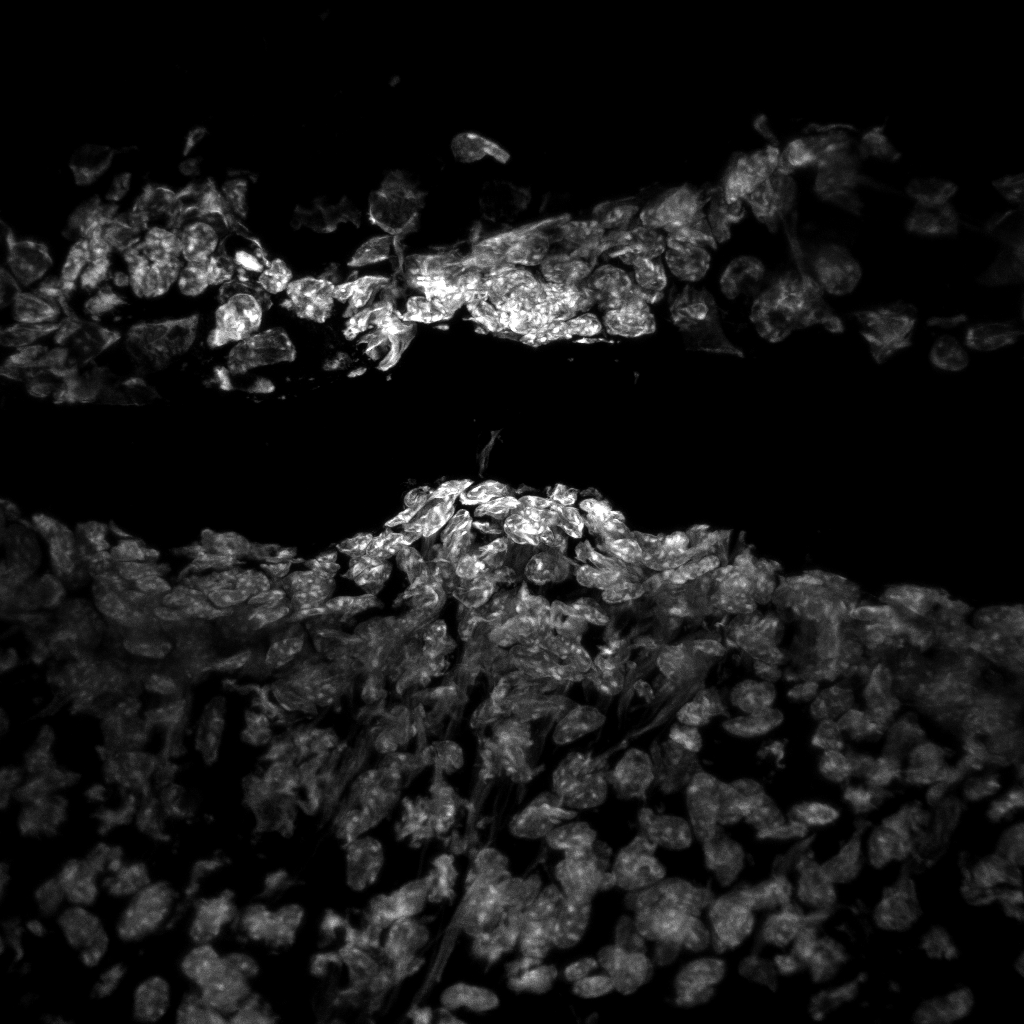

Supplement: Supplementary file 4 — Source Data Fig. 3 [file 44318_2024_39_MOESM4_ESM.zip › Figure 3/3H/F480 LDHA staining/E18.5 KO PBS F480green LDHAred 40X.tif]

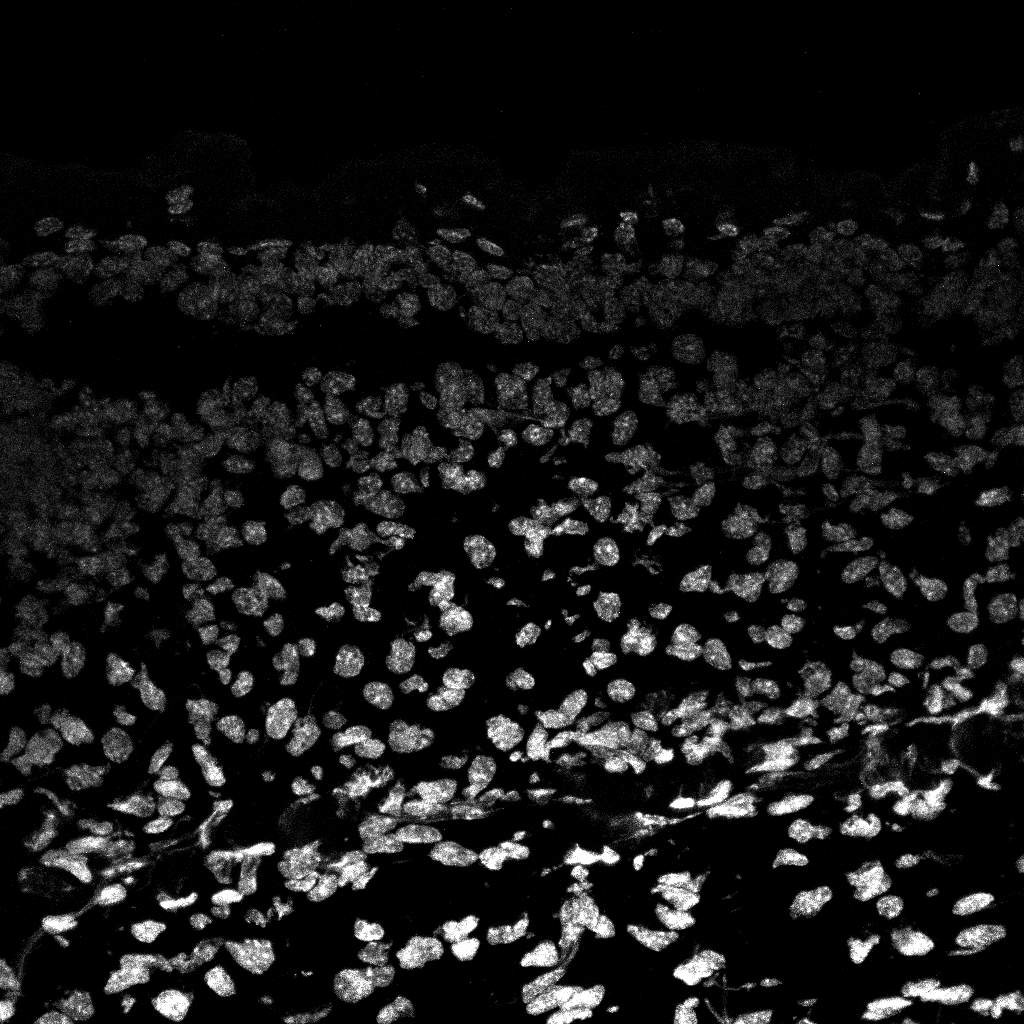

Supplement: Supplementary file 4 — Source Data Fig. 3 [file 44318_2024_39_MOESM4_ESM.zip › Figure 3/3H/F480 GLUT1 staining/E18.5 NAC F480green GLUT1red 40X.tif]

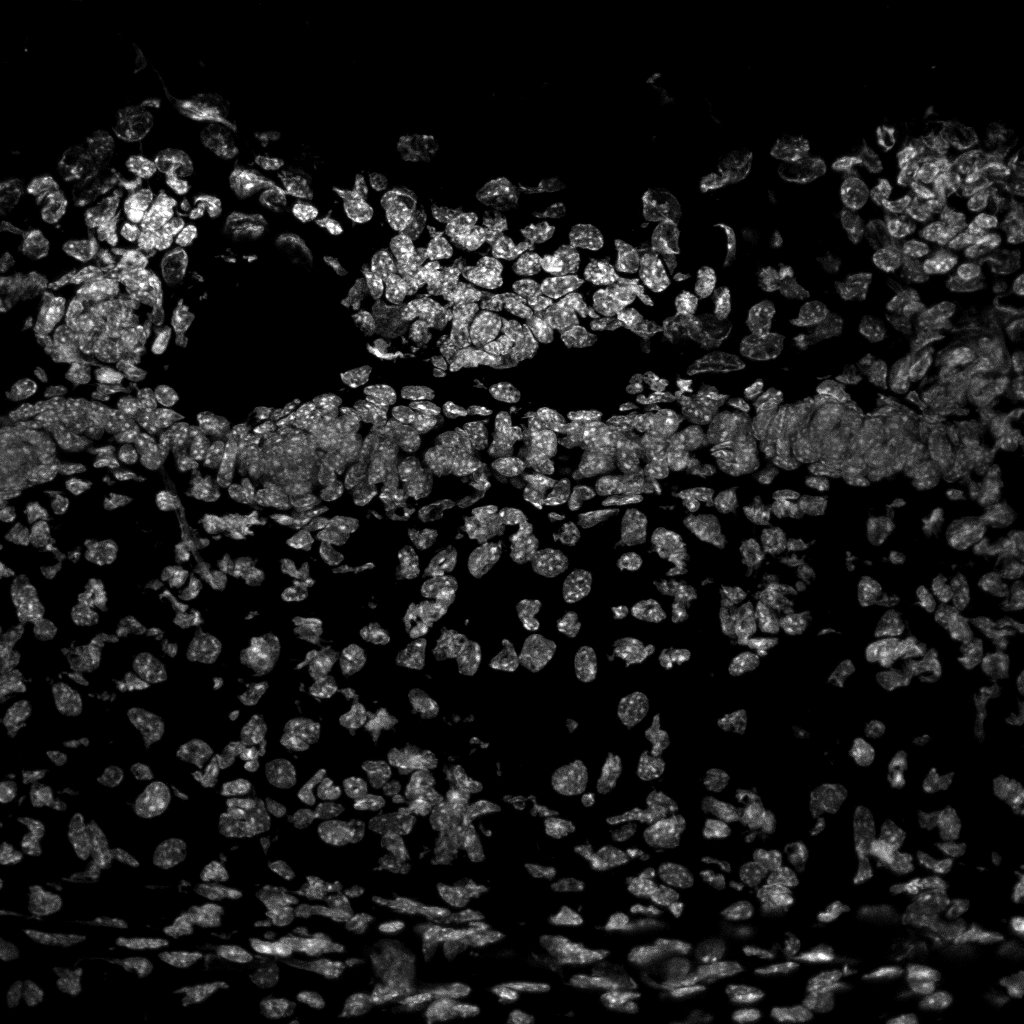

Supplement: Supplementary file 4 — Source Data Fig. 3 [file 44318_2024_39_MOESM4_ESM.zip › Figure 3/3H/F480 GLUT1 staining/E18.5 KO PBS F480green GLUT1red 40X.tif]

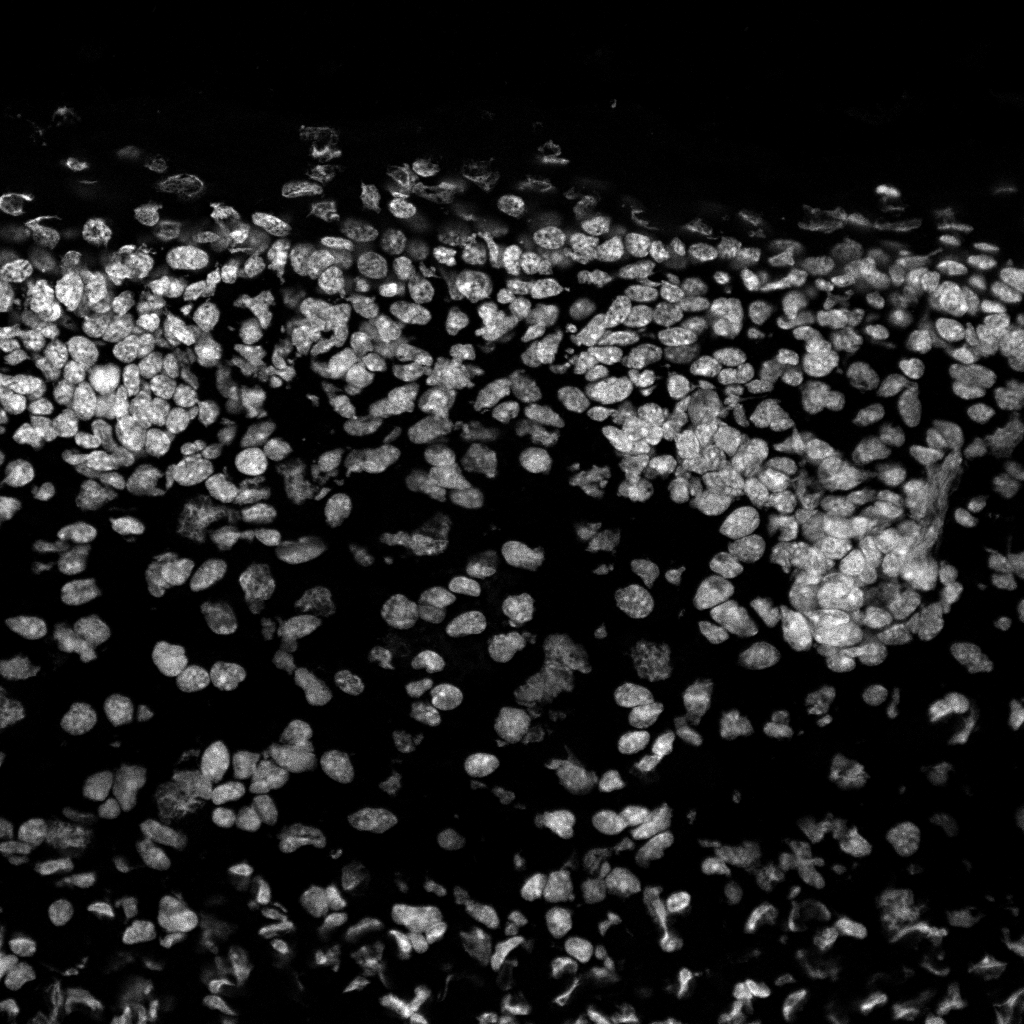

Supplement: Supplementary file 4 — Source Data Fig. 3 [file 44318_2024_39_MOESM4_ESM.zip › Figure 3/3F/GSH ECAD staining/E18.5 WT ECADgreen GSHred 40X.tif]

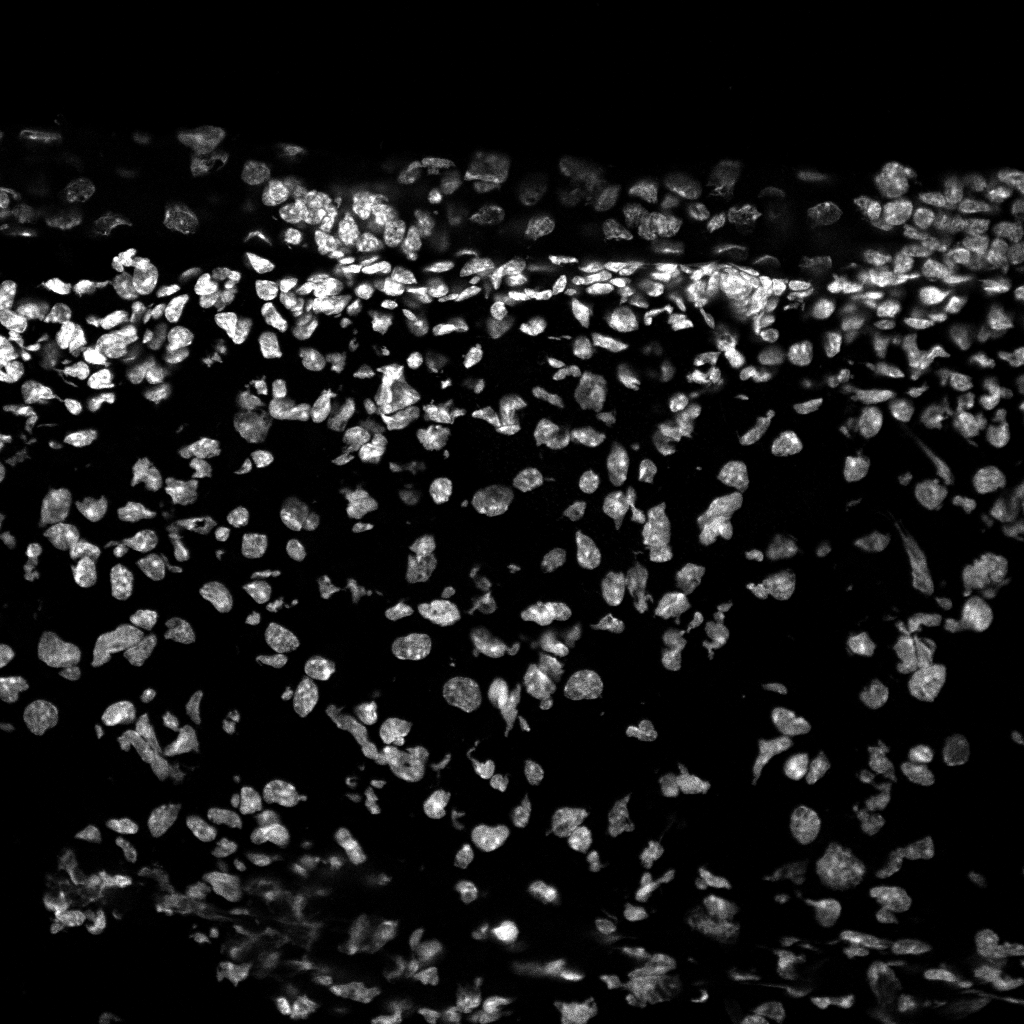

Supplement: Supplementary file 4 — Source Data Fig. 3 [file 44318_2024_39_MOESM4_ESM.zip › Figure 3/3F/GSH ECAD staining/E18.5 KO ECADgreen GSHred 40X.tif]

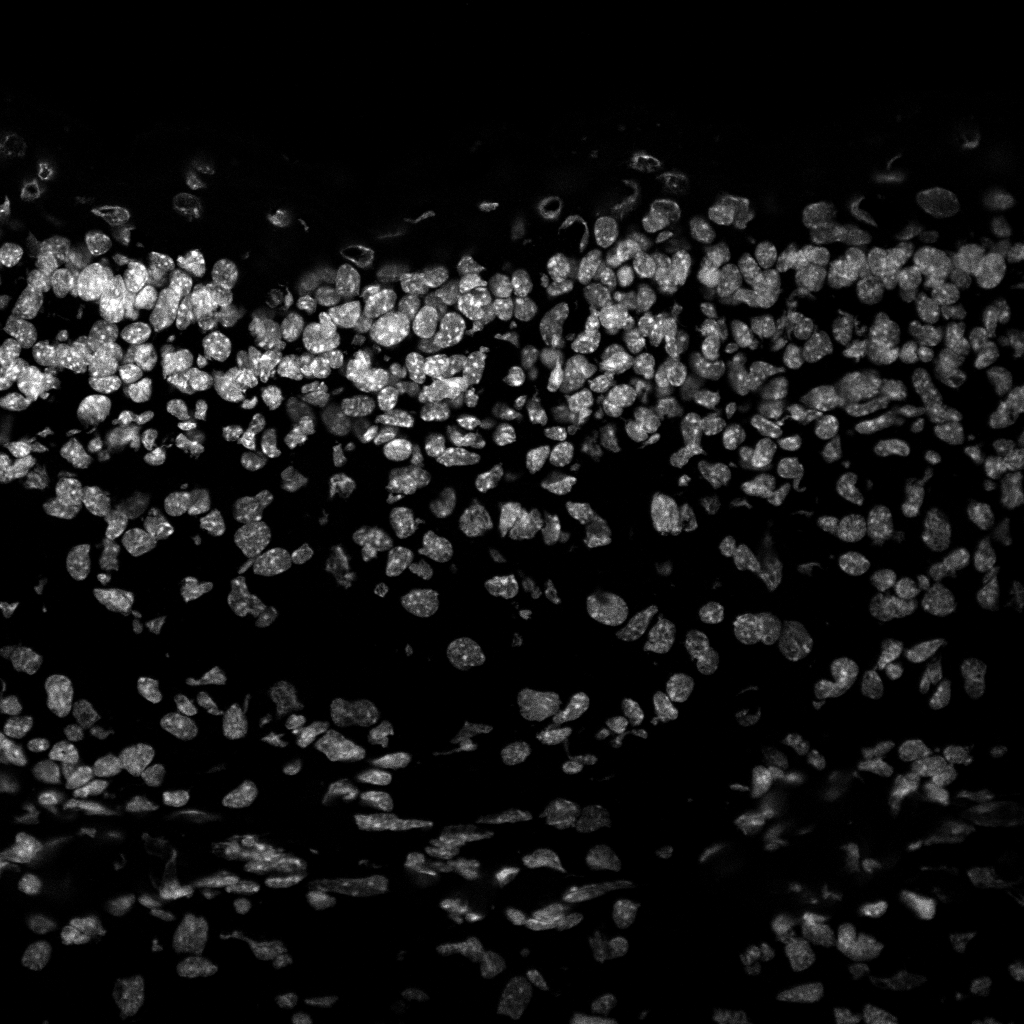

Supplement: Supplementary file 4 — Source Data Fig. 3 [file 44318_2024_39_MOESM4_ESM.zip › Figure 3/3F/ECAD CATALASE staining/E18.5 KO ECADgreen CATALASEred 40X.tif]

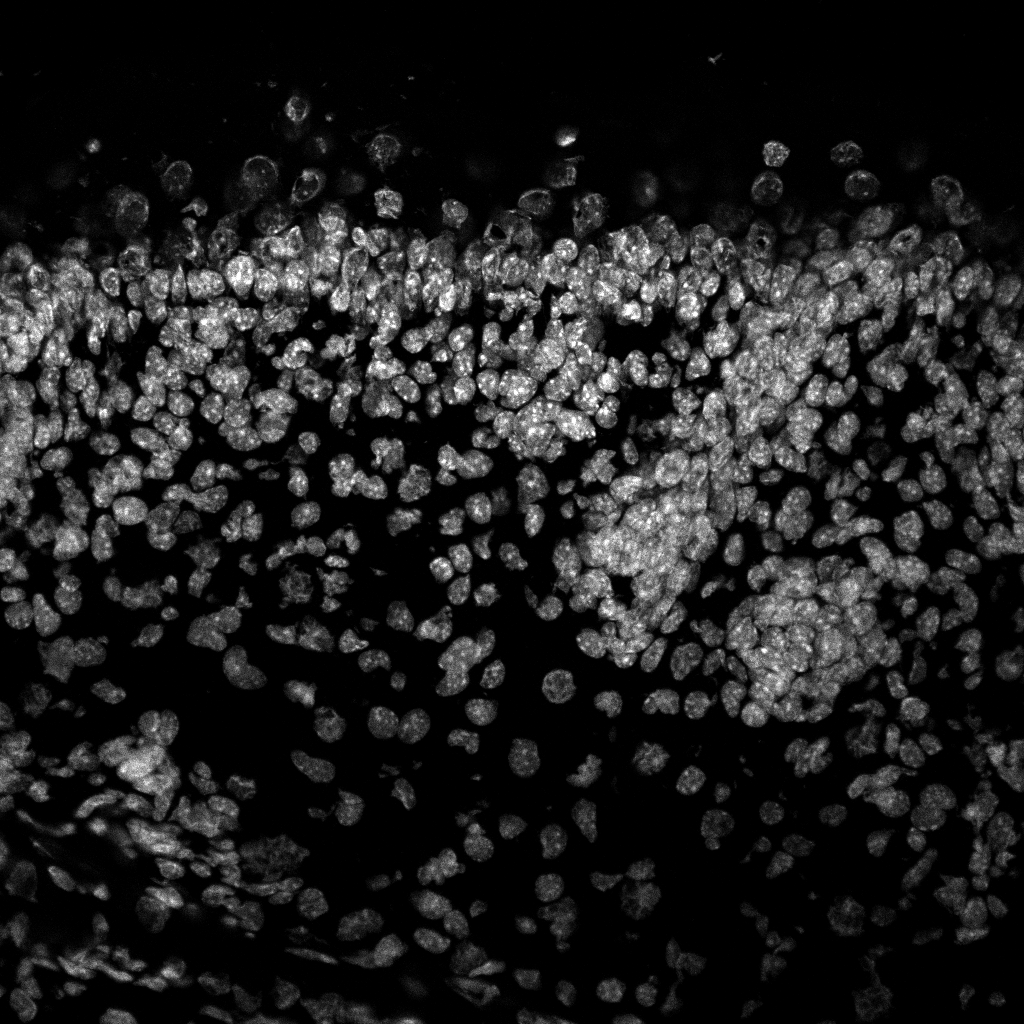

Supplement: Supplementary file 4 — Source Data Fig. 3 [file 44318_2024_39_MOESM4_ESM.zip › Figure 3/3F/ECAD CATALASE staining/E18.5 WT ECADgreen CATALASEred 40X.tif]

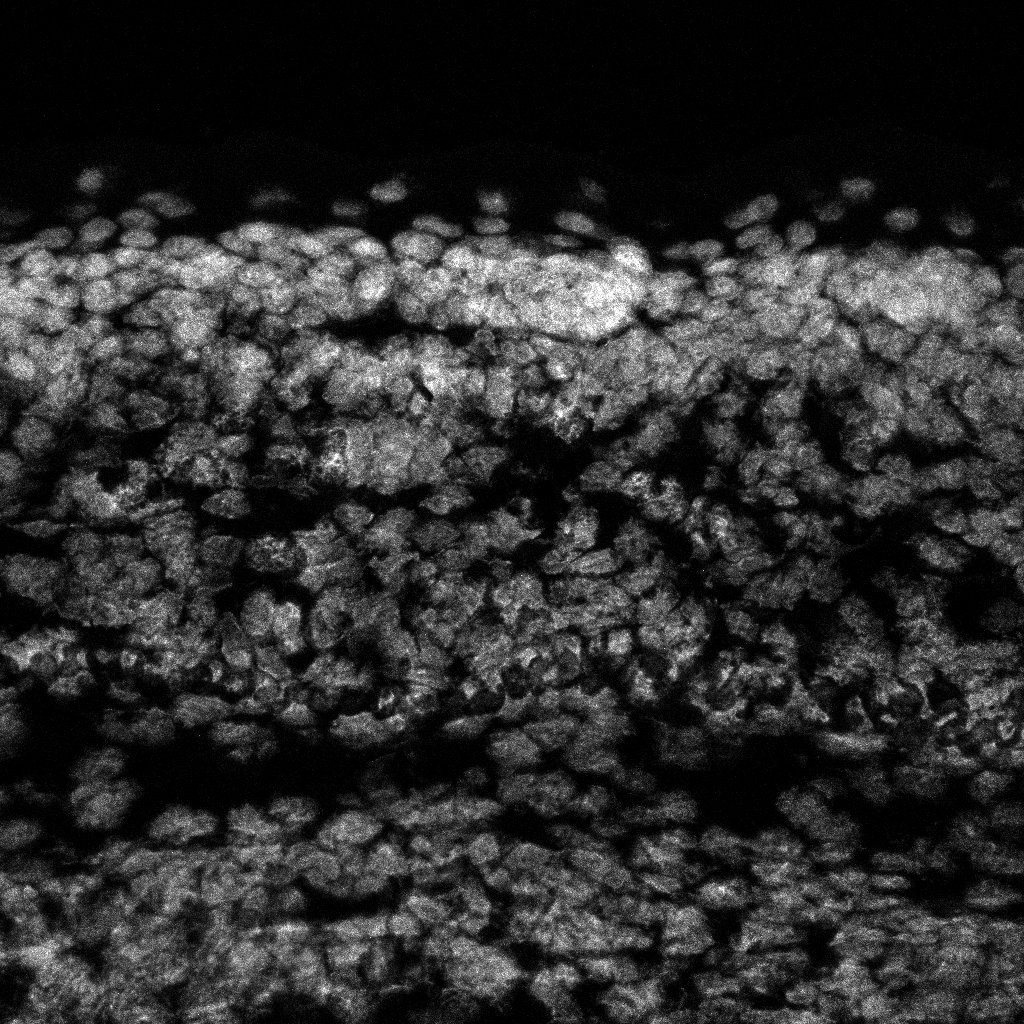

Supplement: Supplementary file 4 — Source Data Fig. 3 [file 44318_2024_39_MOESM4_ESM.zip › Figure 3/3G/HIF1A ECAD staining/E18.5 NAC KO ECADgreen HIF1ared 40X.tif]

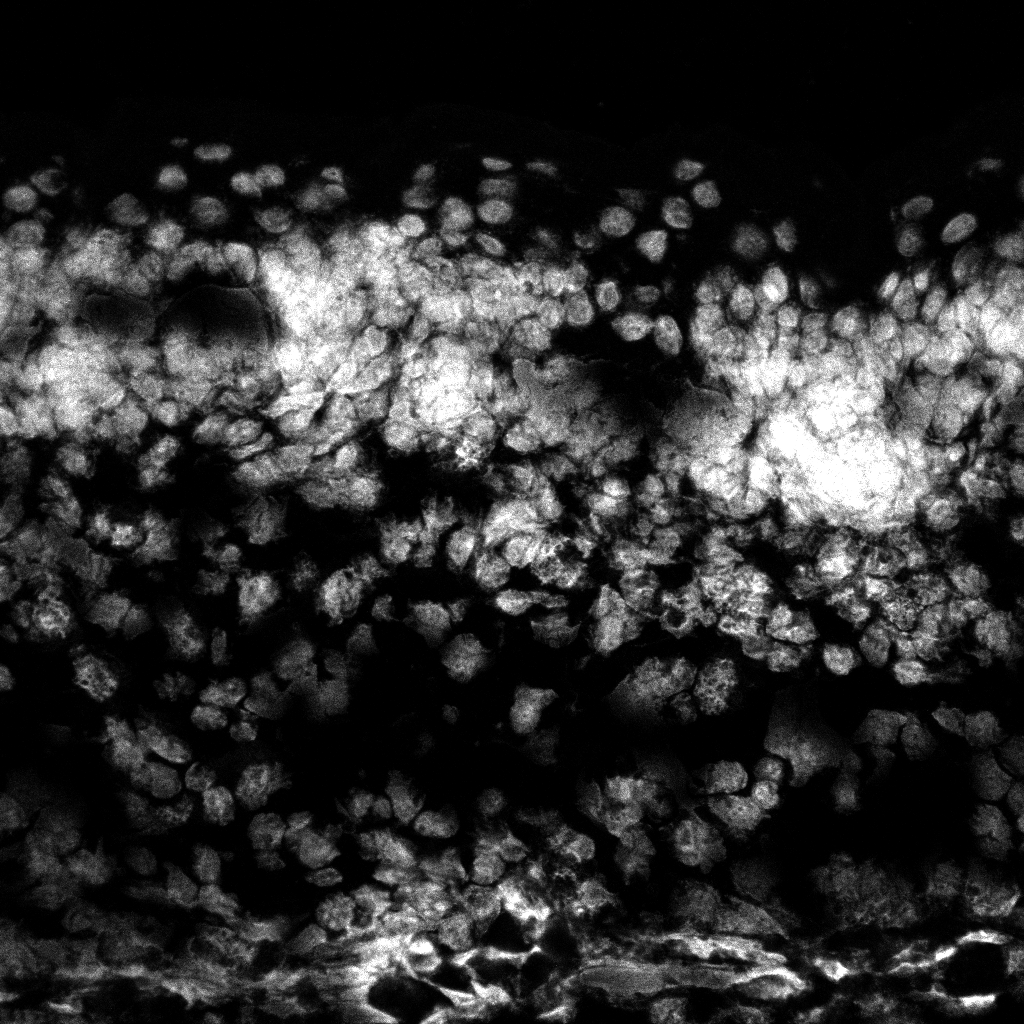

Supplement: Supplementary file 4 — Source Data Fig. 3 [file 44318_2024_39_MOESM4_ESM.zip › Figure 3/3G/HIF1A ECAD staining/E18.5 PBS ECADgreen HIF1ared 40X.tif]

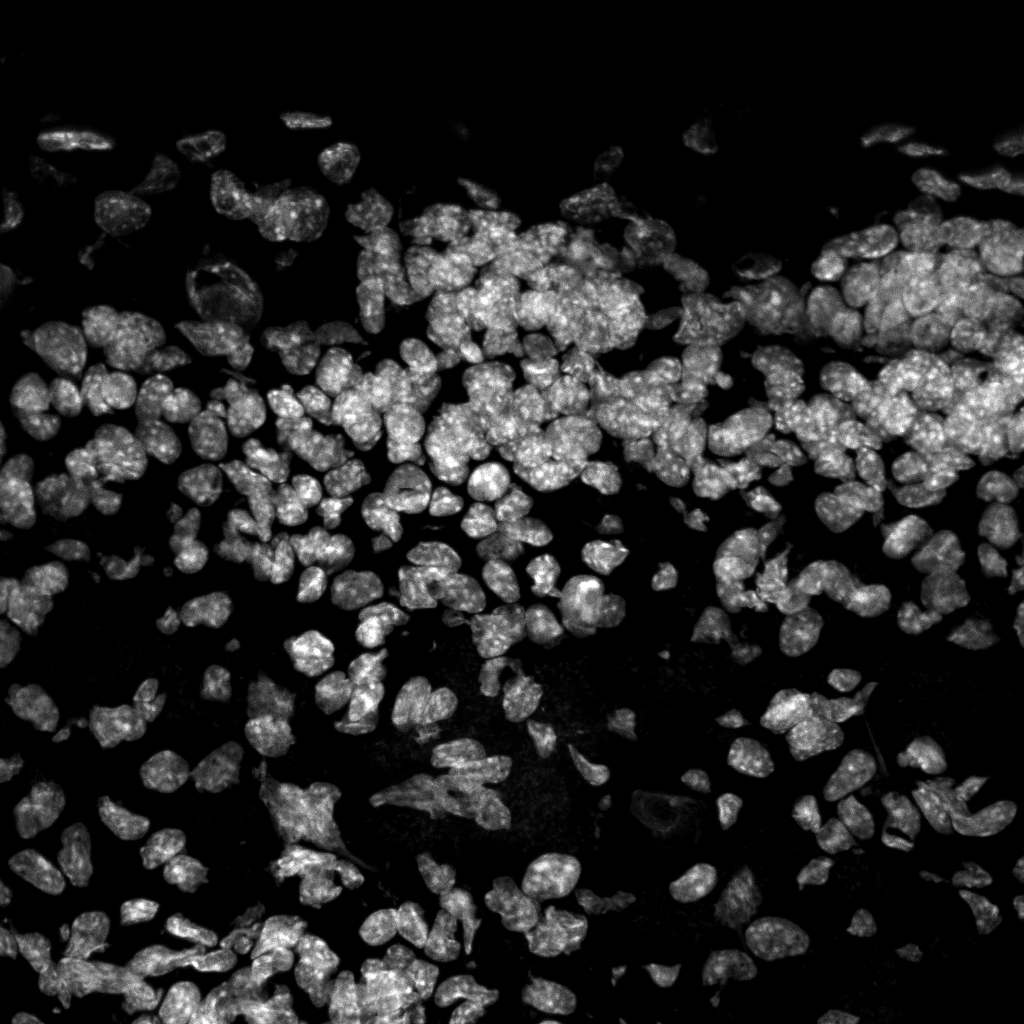

Supplement: Supplementary file 4 — Source Data Fig. 3 [file 44318_2024_39_MOESM4_ESM.zip › Figure 3/3G/8OHDG ECAD staining/E18.5 PBS KO ECADgrey 8OHDG 40X 1.5X.tif]

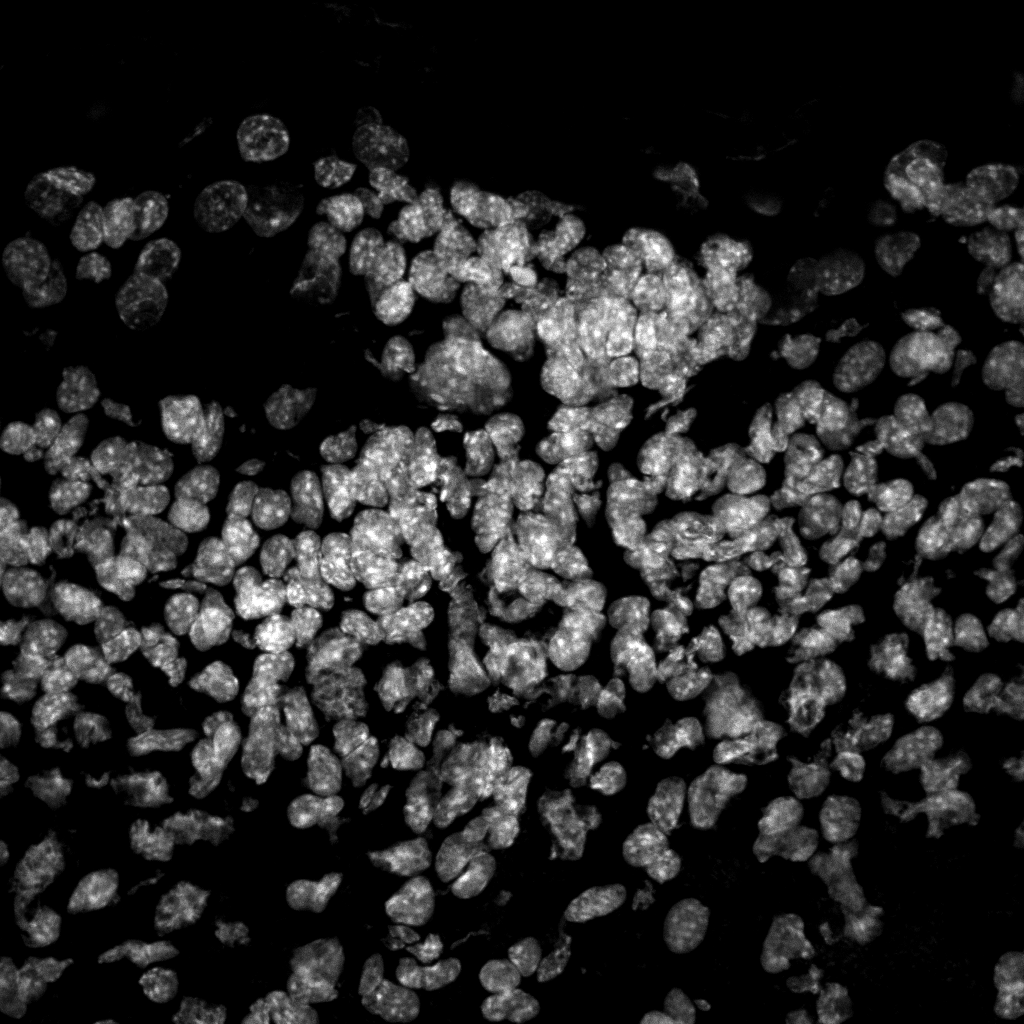

Supplement: Supplementary file 4 — Source Data Fig. 3 [file 44318_2024_39_MOESM4_ESM.zip › Figure 3/3G/8OHDG ECAD staining/E18.5 NAC KO ECADgrey 8OHDG 40X 1.5X.tif]

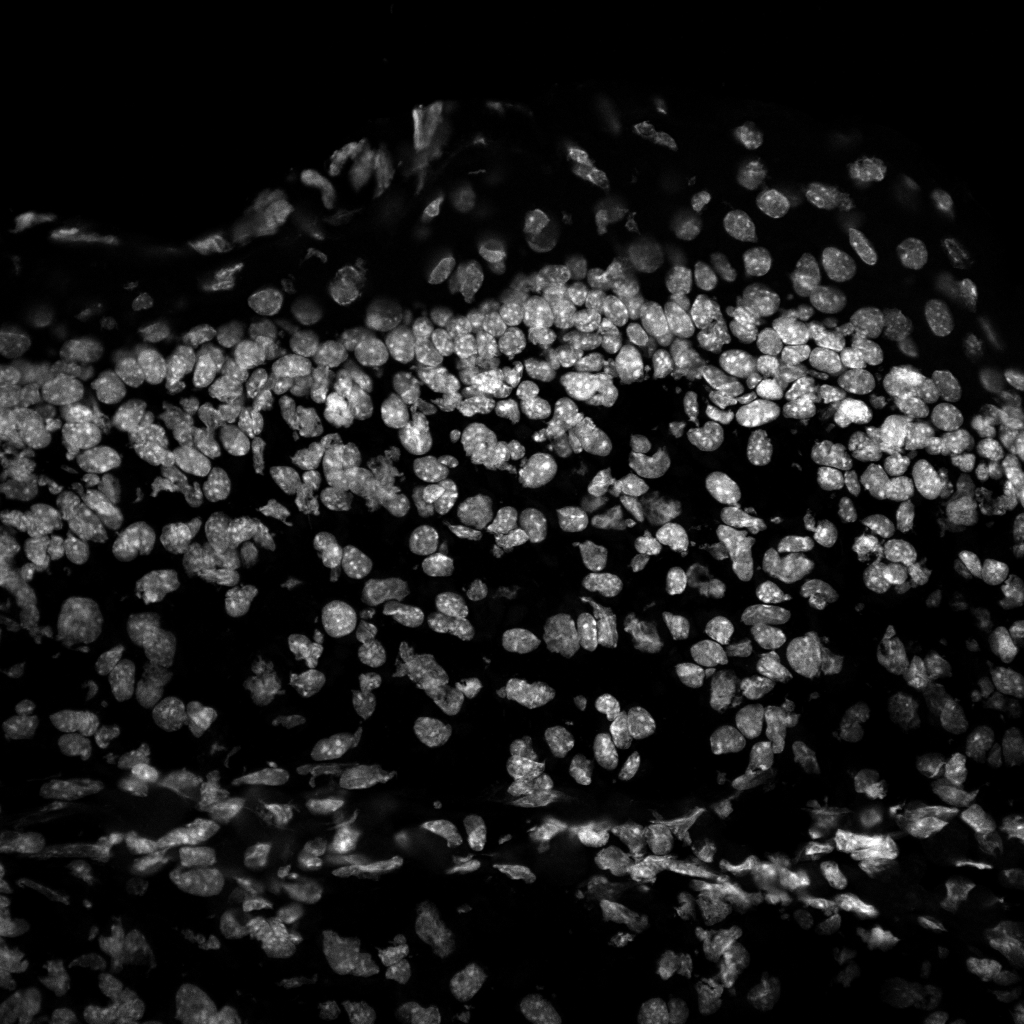

Supplement: Supplementary file 5 — Source Data Fig. 4 [file 44318_2024_39_MOESM5_ESM.zip › Figure 4/4A/E17.5 KO MCT4green 40X.tif]

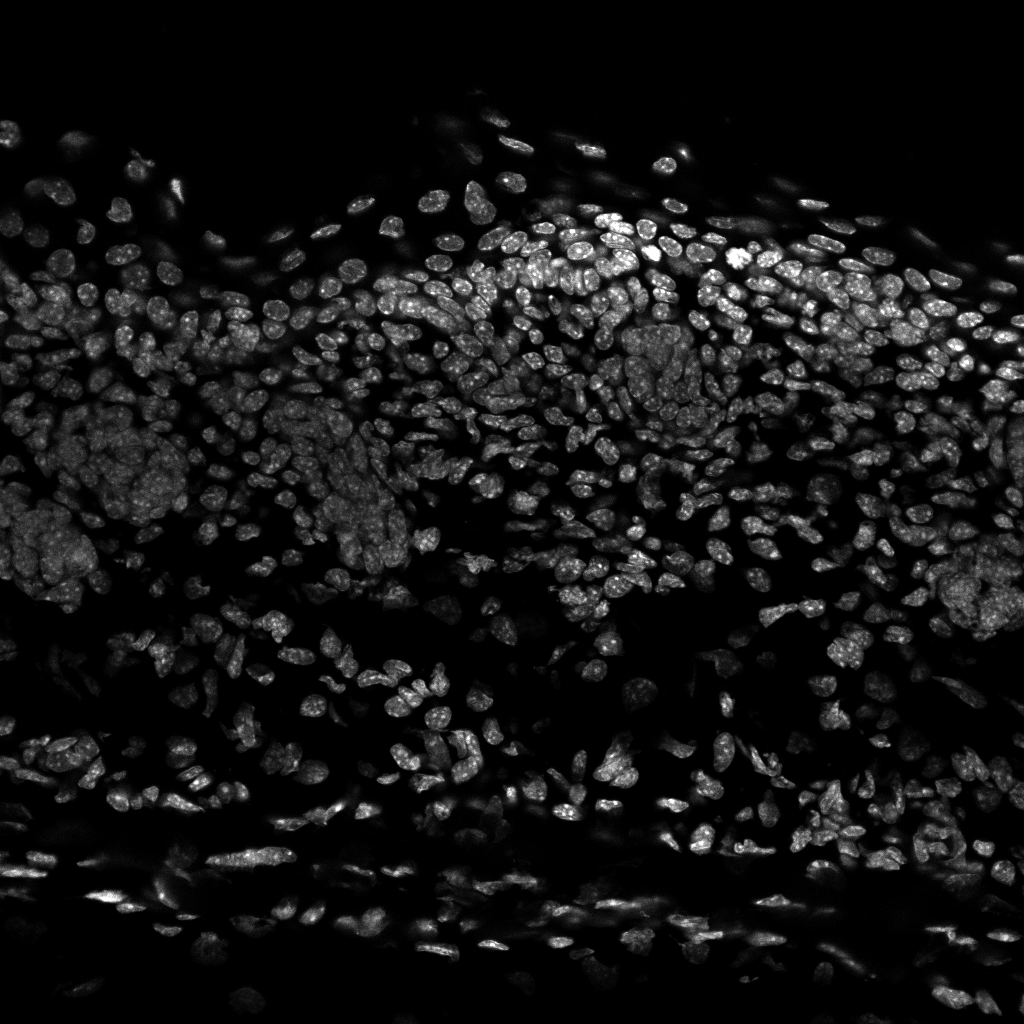

Supplement: Supplementary file 5 — Source Data Fig. 4 [file 44318_2024_39_MOESM5_ESM.zip › Figure 4/4A/E18.5 WT MCT4green 40X.tif]

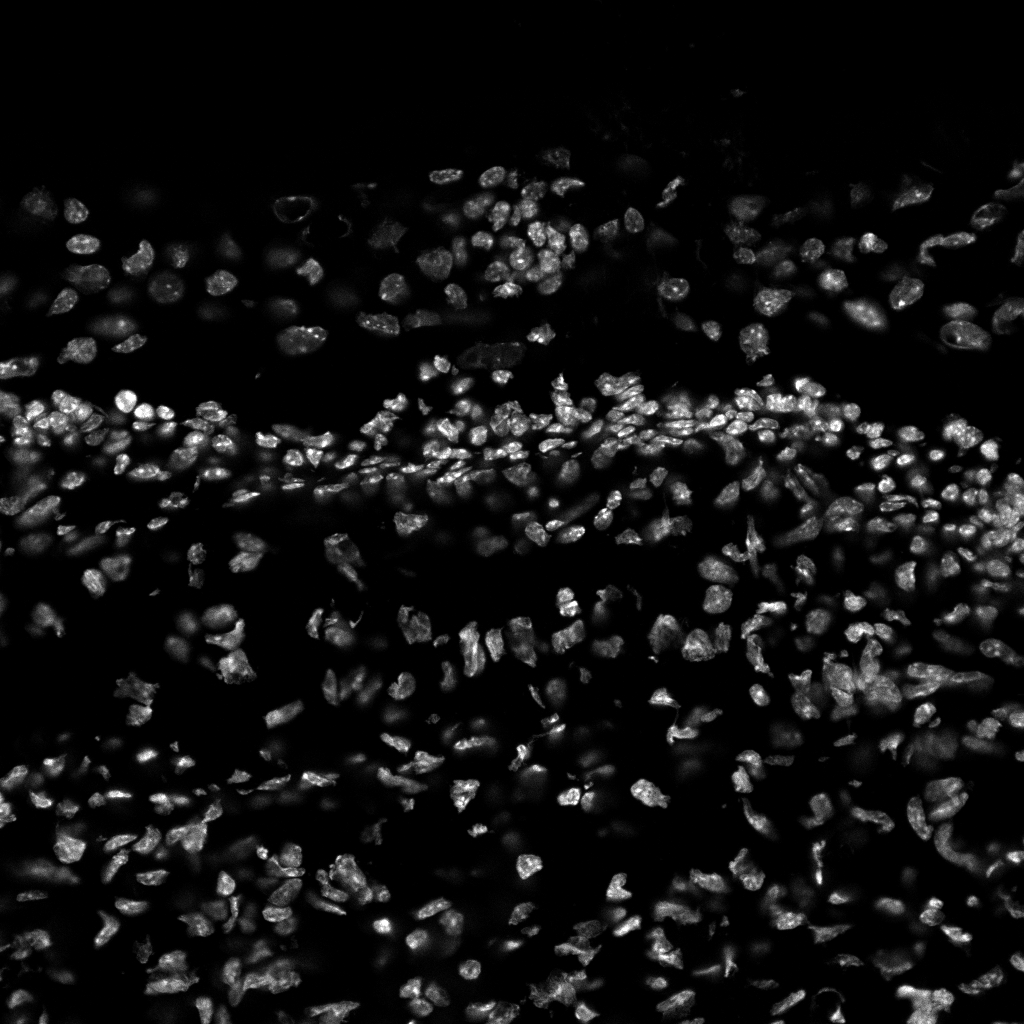

Supplement: Supplementary file 5 — Source Data Fig. 4 [file 44318_2024_39_MOESM5_ESM.zip › Figure 4/4A/E18.5 KO MCT4green 40X.tif]

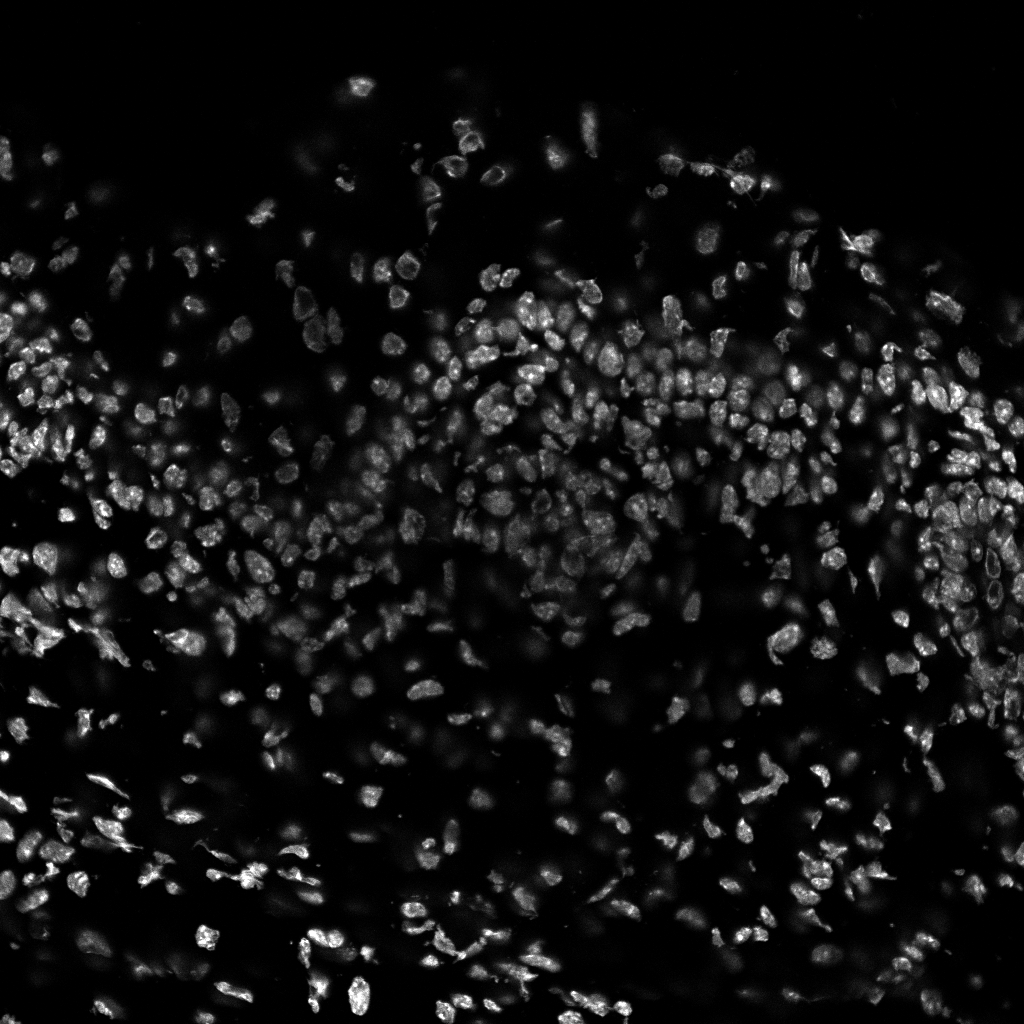

Supplement: Supplementary file 5 — Source Data Fig. 4 [file 44318_2024_39_MOESM5_ESM.zip › Figure 4/4A/E17.5 WT MCT4green 40X.tif]

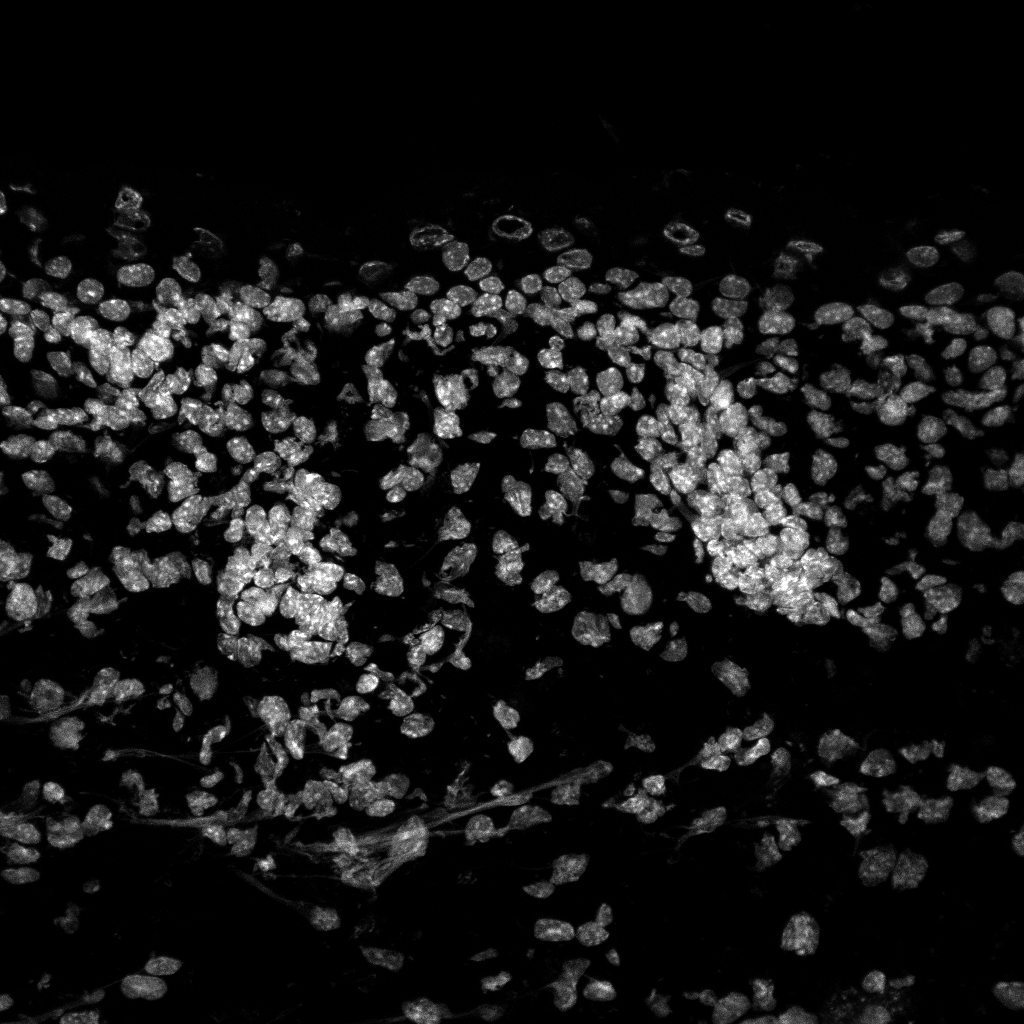

Supplement: Supplementary file 5 — Source Data Fig. 4 [file 44318_2024_39_MOESM5_ESM.zip › Figure 4/4B/F480 CS staining/E18.5 WT F480green CSred 40X.tif]

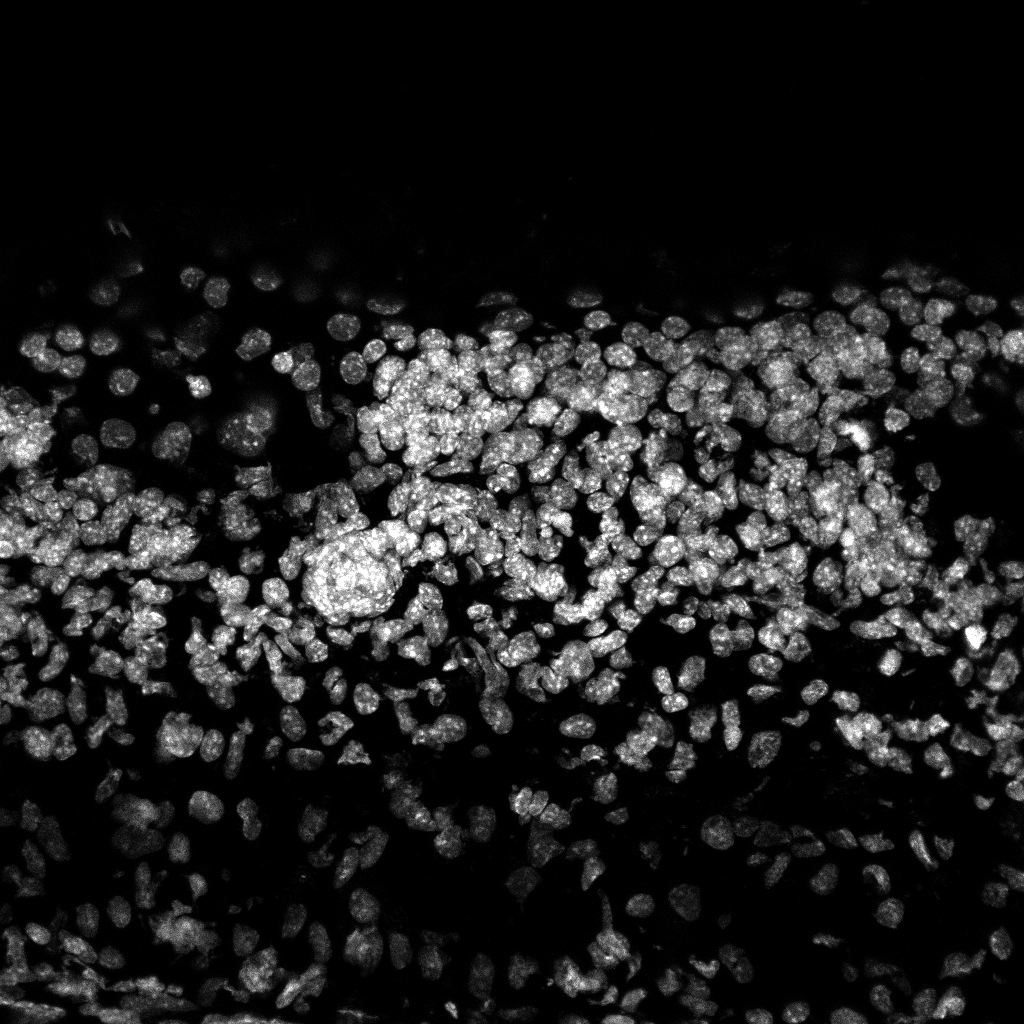

Supplement: Supplementary file 5 — Source Data Fig. 4 [file 44318_2024_39_MOESM5_ESM.zip › Figure 4/4B/F480 CS staining/E18.5 KO F480green CSred 40X.tif]

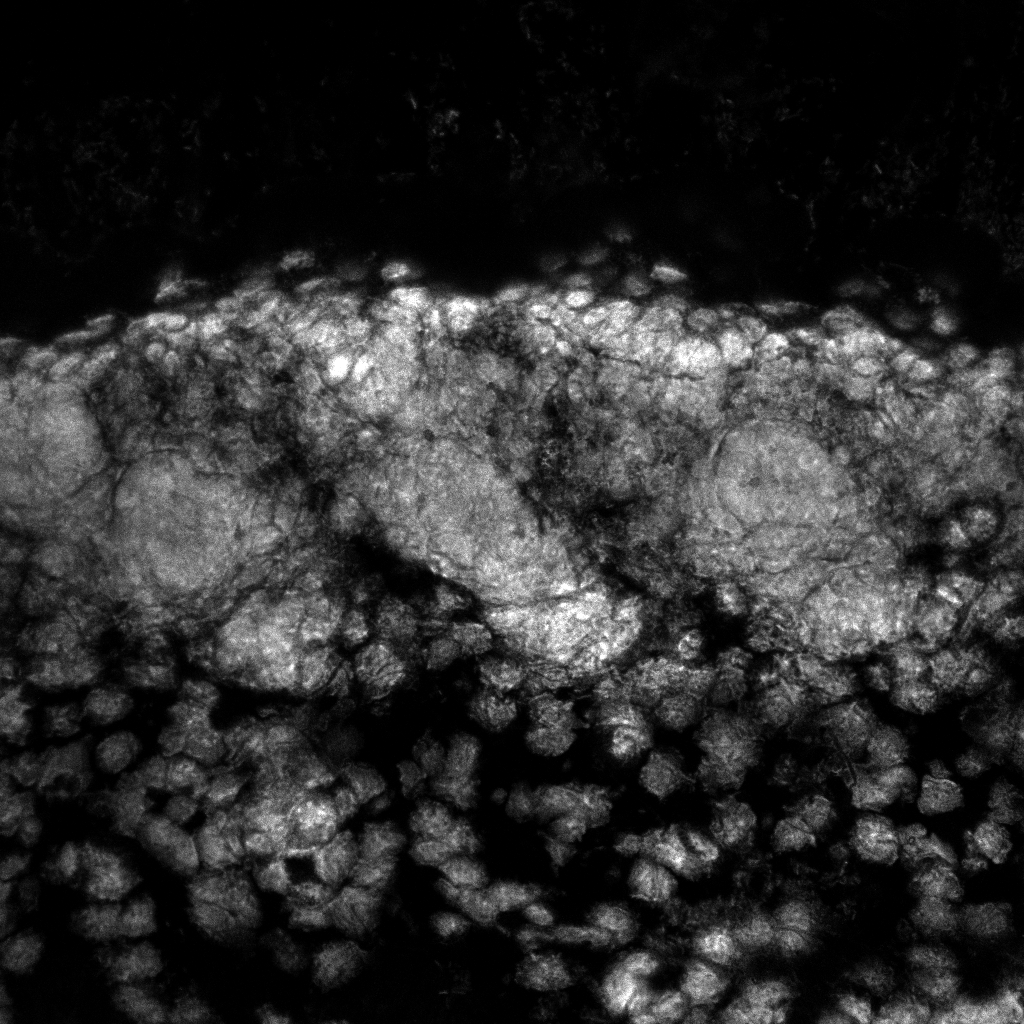

Supplement: Supplementary file 5 — Source Data Fig. 4 [file 44318_2024_39_MOESM5_ESM.zip › Figure 4/4B/IDH1 F480 staining/E18.5 WT IDH1red F480green 40X.tif]

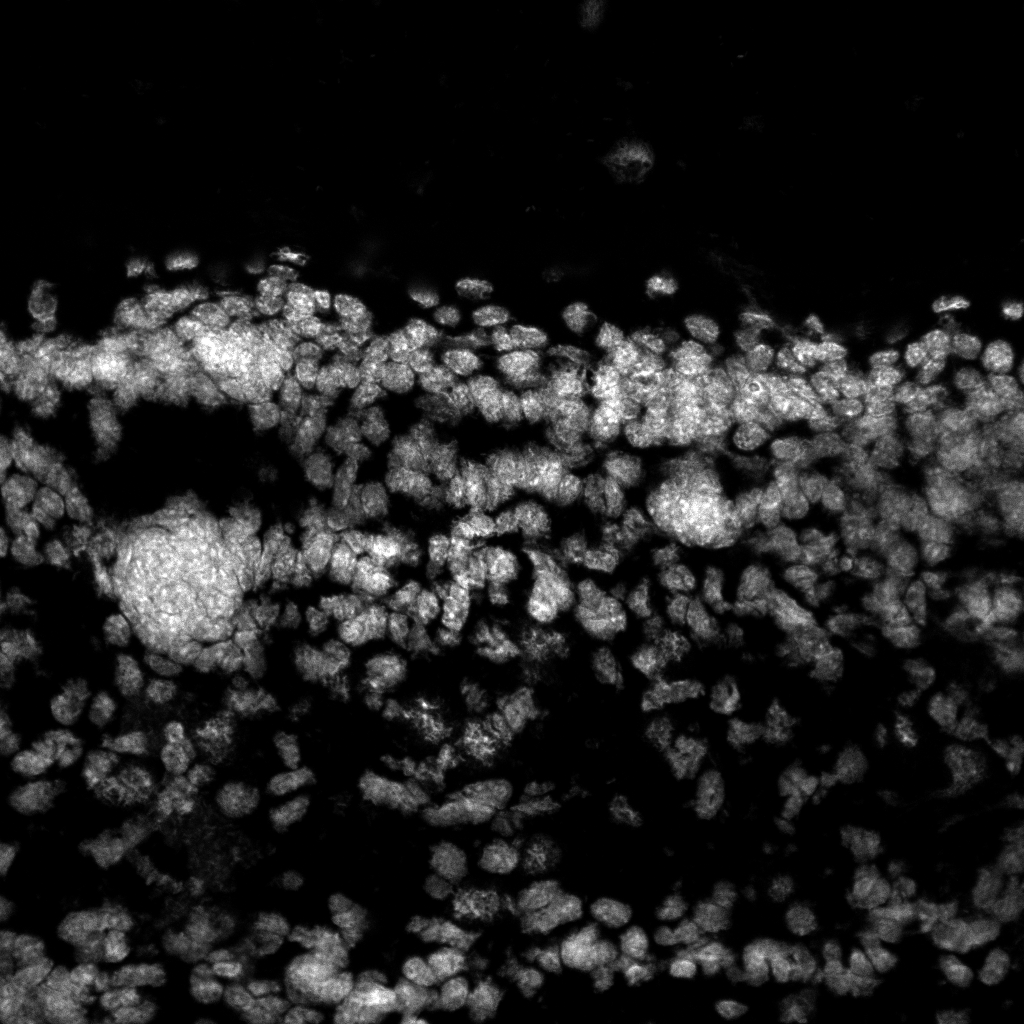

Supplement: Supplementary file 5 — Source Data Fig. 4 [file 44318_2024_39_MOESM5_ESM.zip › Figure 4/4B/IDH1 F480 staining/E18.5 KO IDH1red F480green 40X.tif]

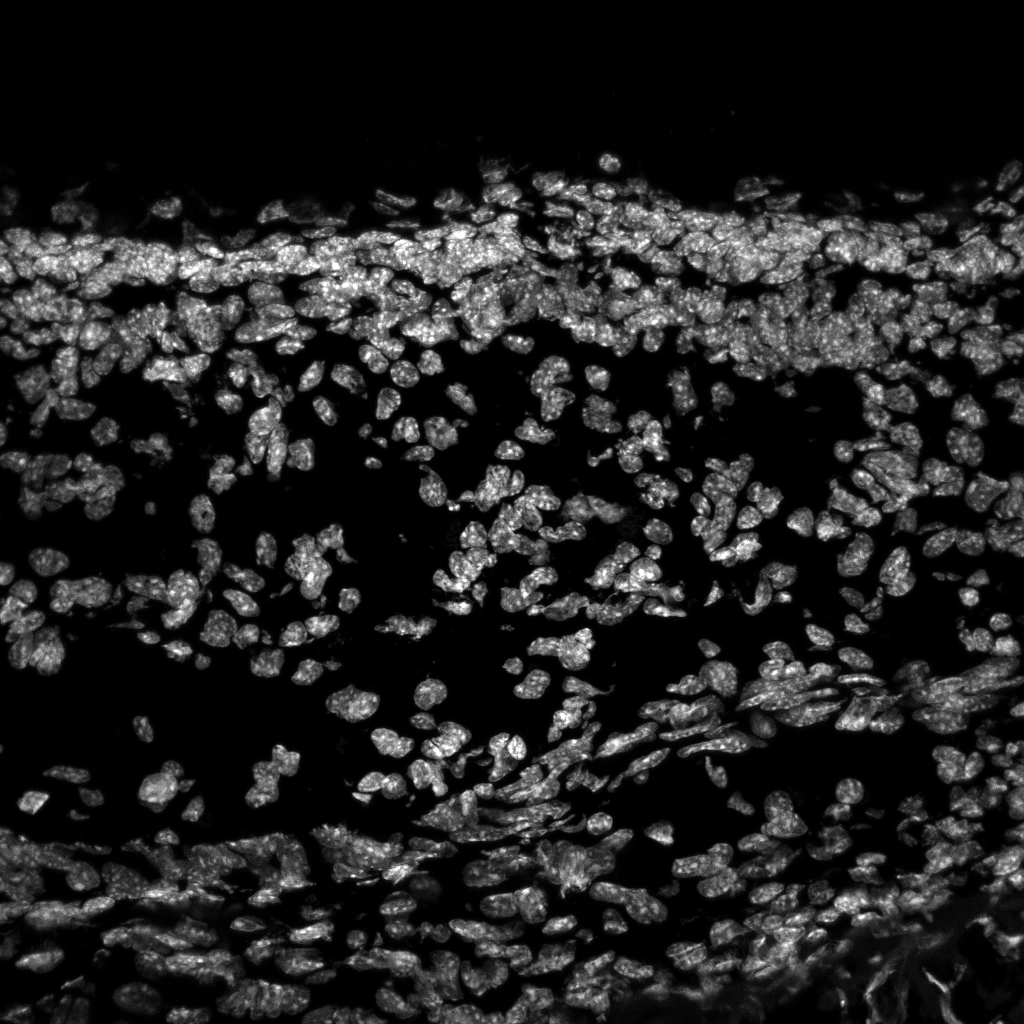

Supplement: Supplementary file 5 — Source Data Fig. 4 [file 44318_2024_39_MOESM5_ESM.zip › Figure 4/4G/F480 MMP9 staining/E18.5 KO F480green MMP9red 40X.tif]

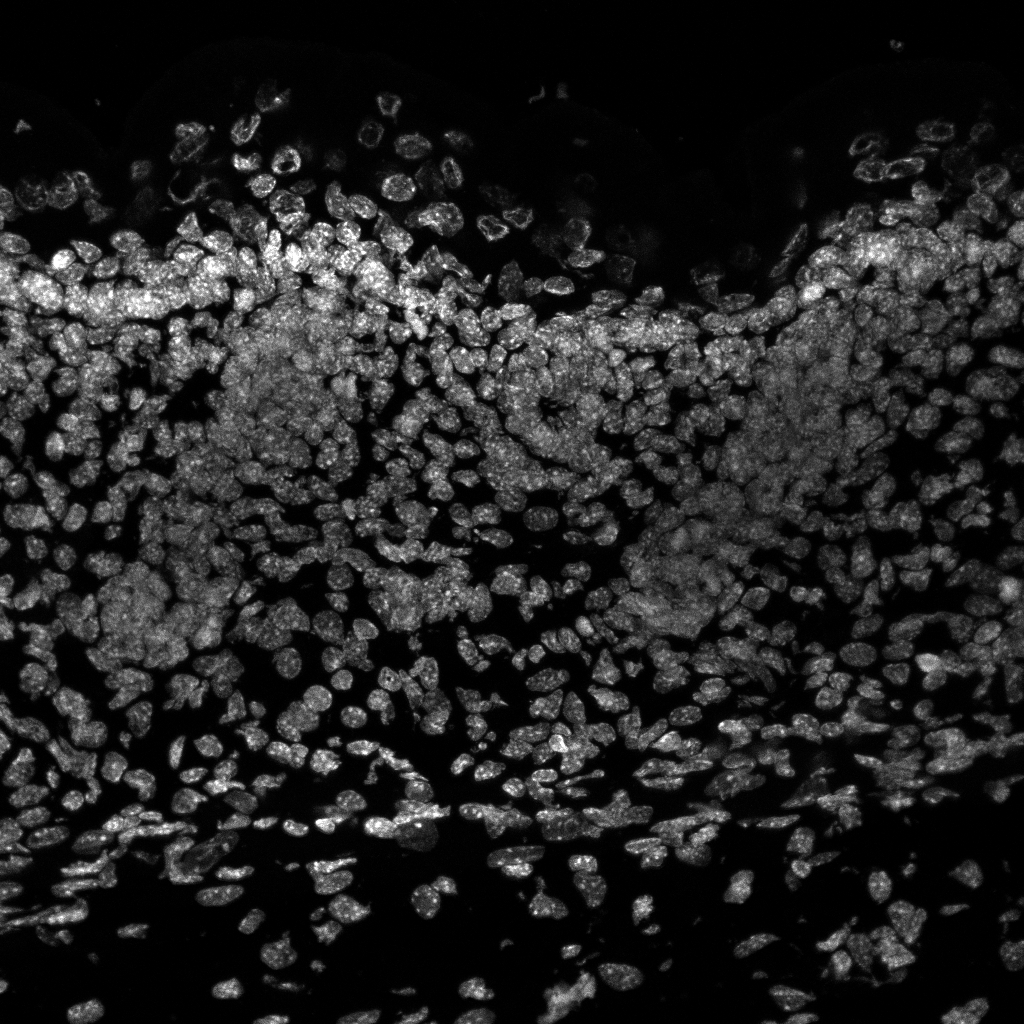

Supplement: Supplementary file 5 — Source Data Fig. 4 [file 44318_2024_39_MOESM5_ESM.zip › Figure 4/4G/F480 MMP9 staining/E18.5 WT F480green MMP9red 40X.tif]

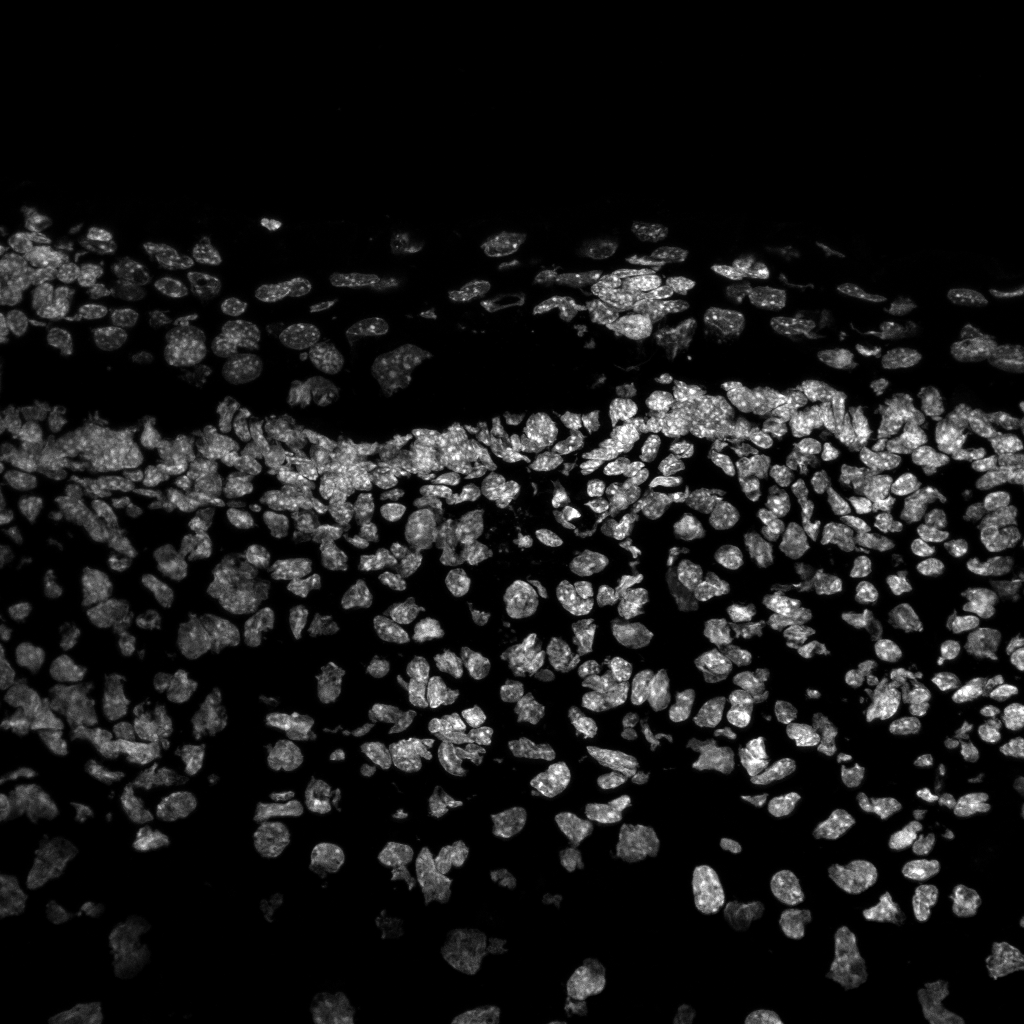

Supplement: Supplementary file 5 — Source Data Fig. 4 [file 44318_2024_39_MOESM5_ESM.zip › Figure 4/4G/BETA1 LAM332 staining/E18.5 KO B1green LAM5red 40X.tif]

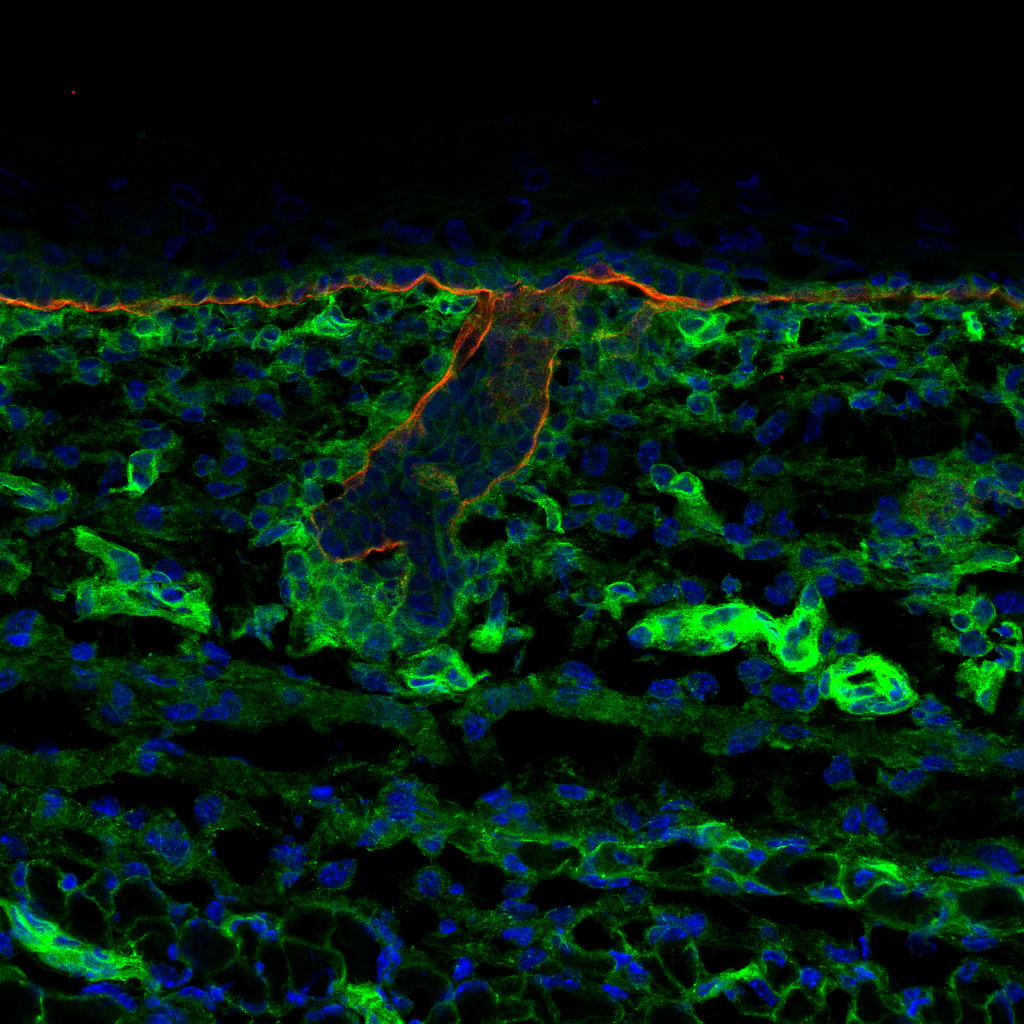

Supplement: Supplementary file 5 — Source Data Fig. 4 [file 44318_2024_39_MOESM5_ESM.zip › Figure 4/4G/BETA1 LAM332 staining/E18.5 WT LAM5red BETA1 green 40X.tif]

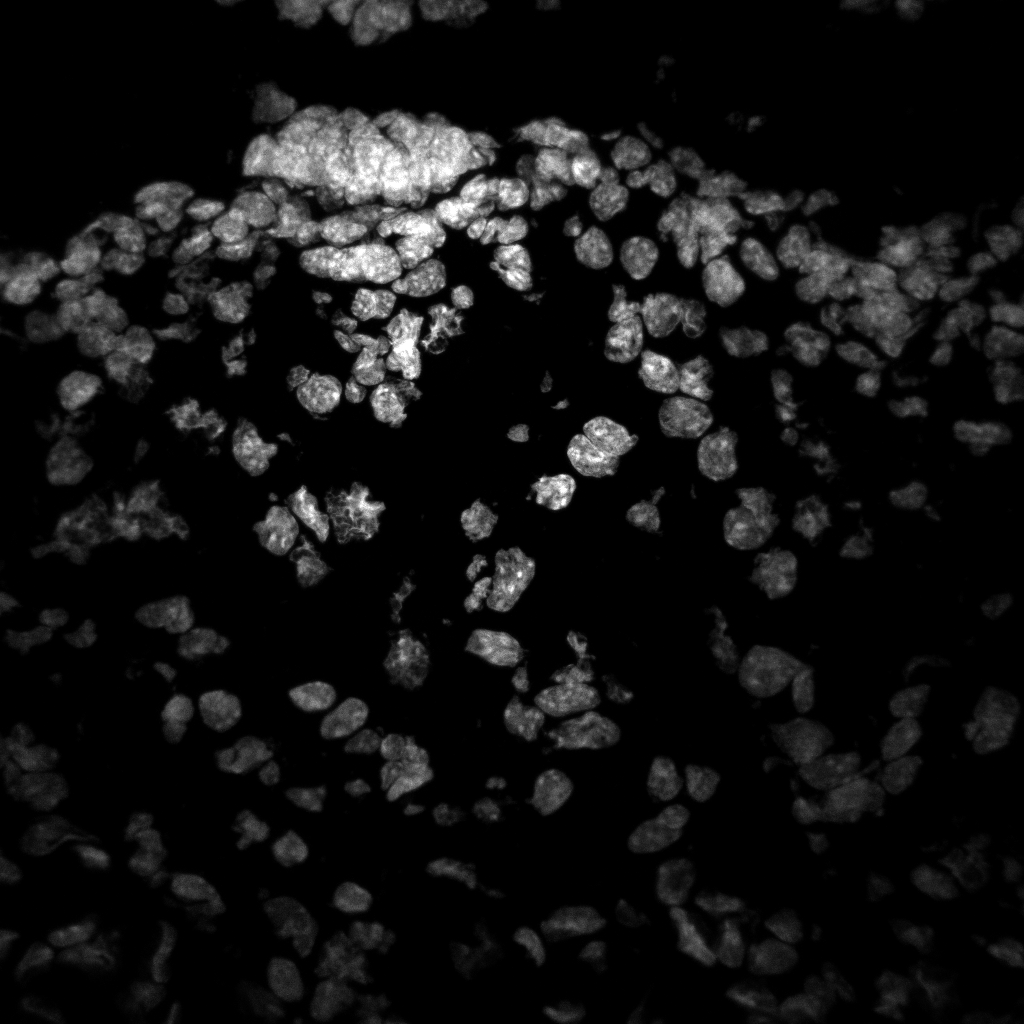

Supplement: Supplementary file 5 — Source Data Fig. 4 [file 44318_2024_39_MOESM5_ESM.zip › Figure 4/4G/F480 MCT1 staining/E18.5 KO F480green MCT1red 40X.tif]

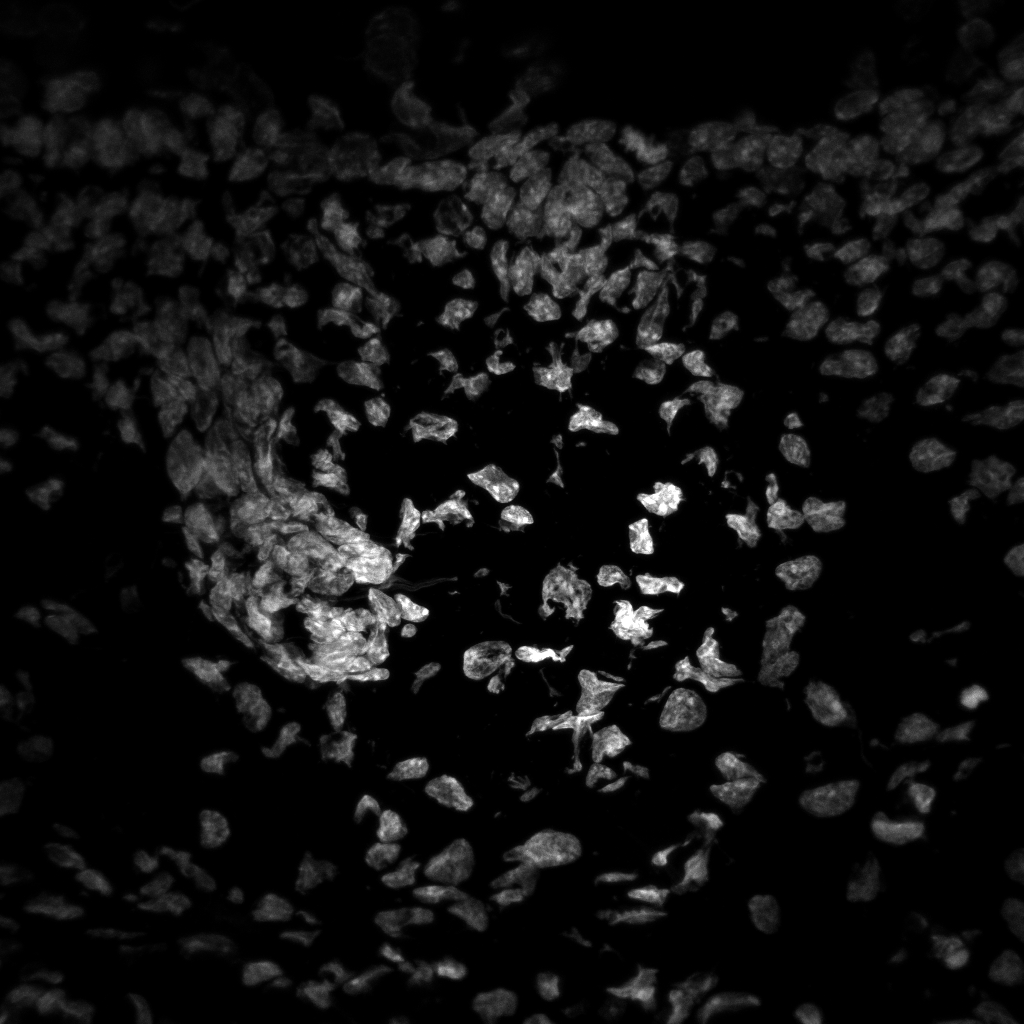

Supplement: Supplementary file 5 — Source Data Fig. 4 [file 44318_2024_39_MOESM5_ESM.zip › Figure 4/4G/F480 MCT1 staining/E18.5 WT F480green MCT1red 40X.tif]

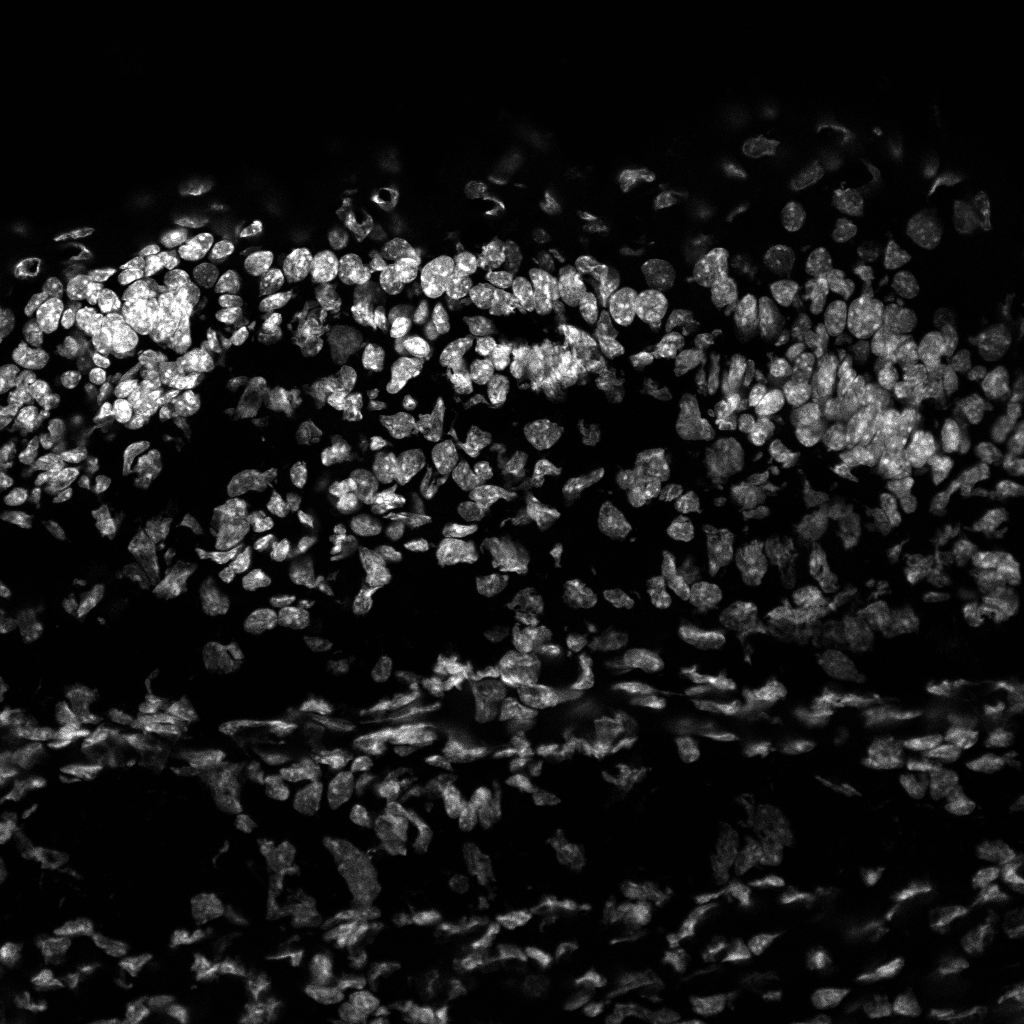

Supplement: Supplementary file 6 — Source Data Fig. 5 [file 44318_2024_39_MOESM6_ESM.zip › Figure 5/5G/E18.5 KO2 SYRO IN UTERO LAM5red BETA1green 40X.tif]

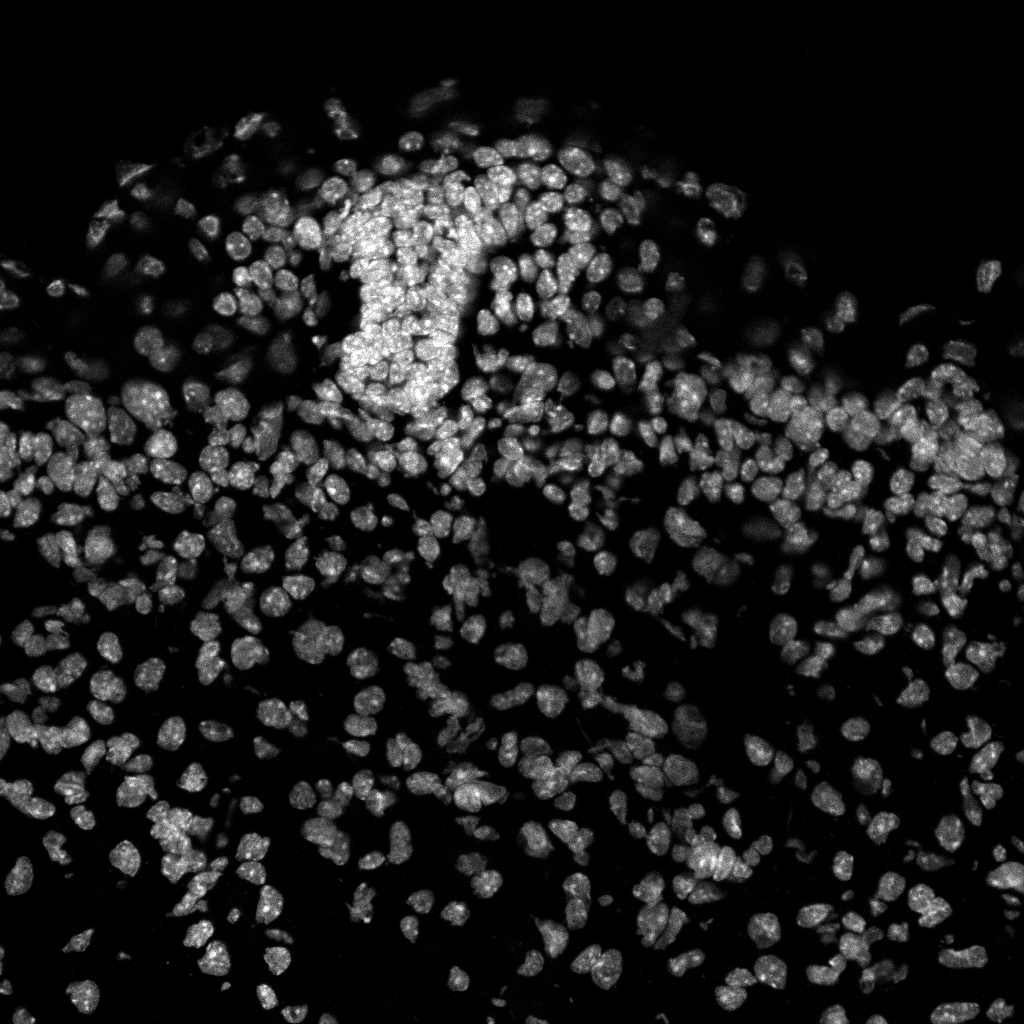

Supplement: Supplementary file 6 — Source Data Fig. 5 [file 44318_2024_39_MOESM6_ESM.zip › Figure 5/5G/E18.5 KO DMSO IN UTERO LAM332red BETA1green 40X.tif]

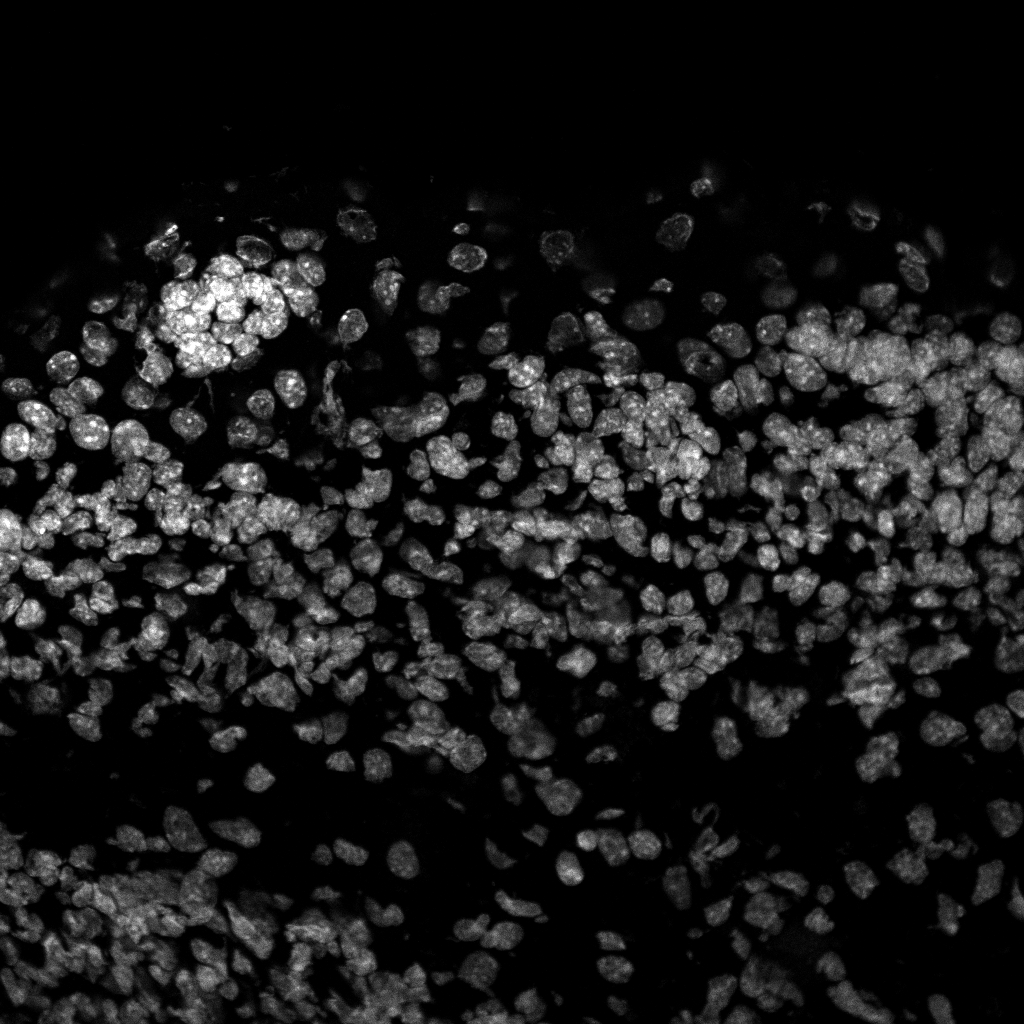

Supplement: Supplementary file 6 — Source Data Fig. 5 [file 44318_2024_39_MOESM6_ESM.zip › Figure 5/5H/SYRO in utero KO F480green MMP9red 40X.tif]

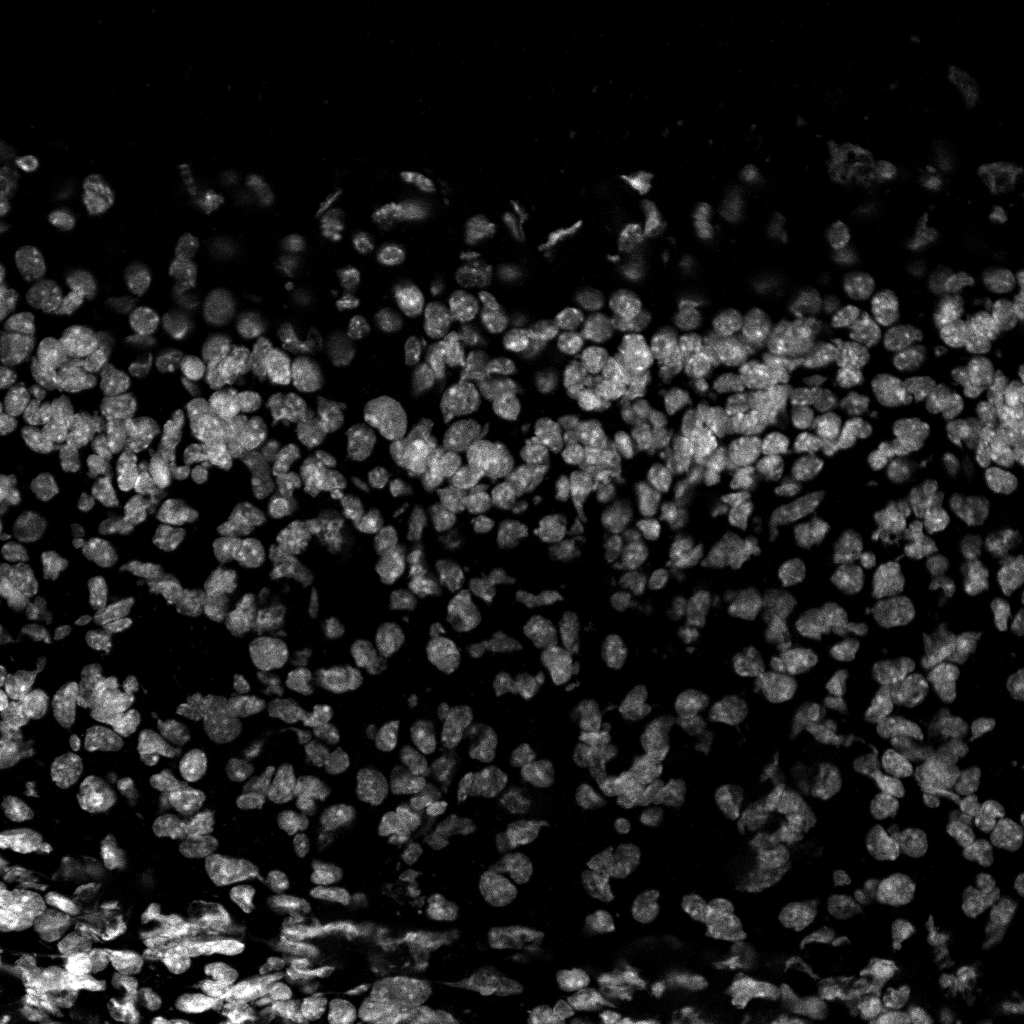

Supplement: Supplementary file 6 — Source Data Fig. 5 [file 44318_2024_39_MOESM6_ESM.zip › Figure 5/5H/DMSO in utero KO F480green MMP9red 40X.tif]

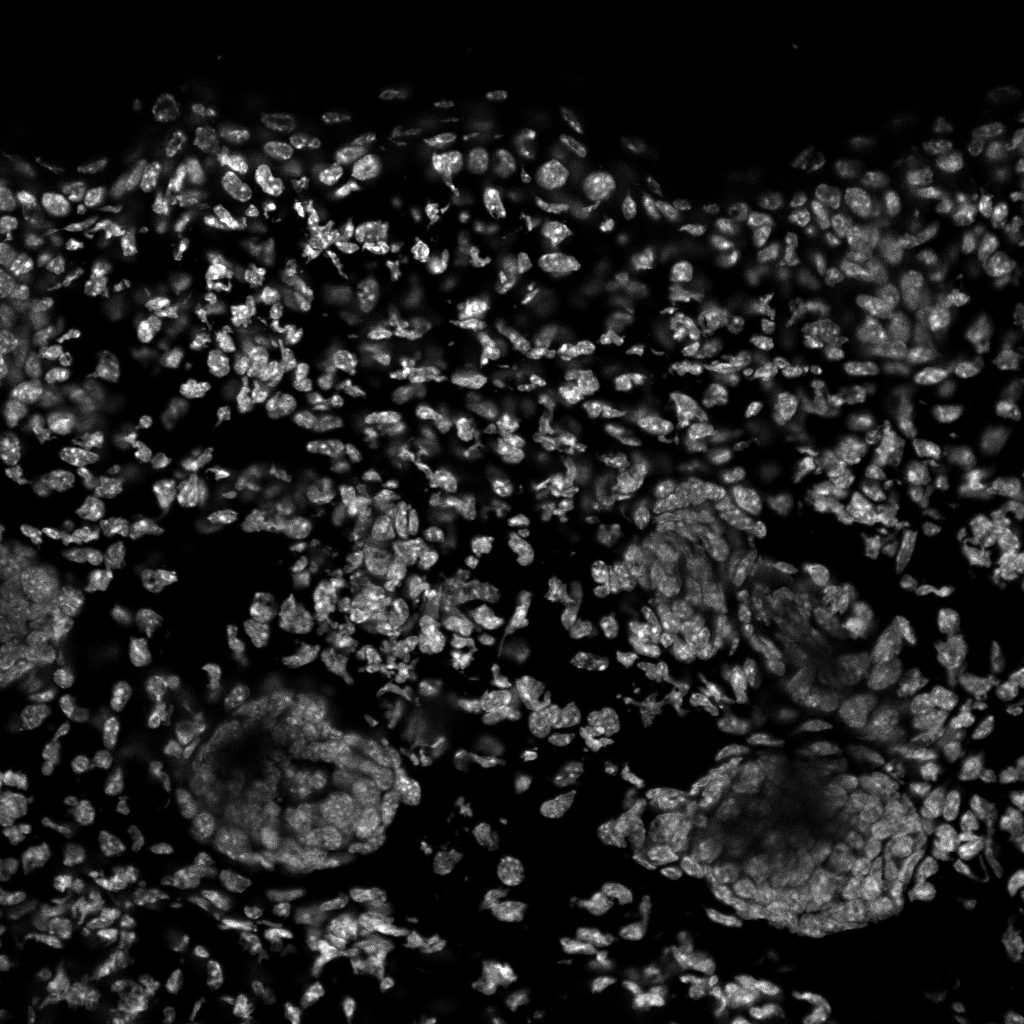

Supplement: Supplementary file 6 — Source Data Fig. 5 [file 44318_2024_39_MOESM6_ESM.zip › Figure 5/5B/P2 NaL treated 0.5mgml F480green CD206red MMP9gray 40x1.tif]

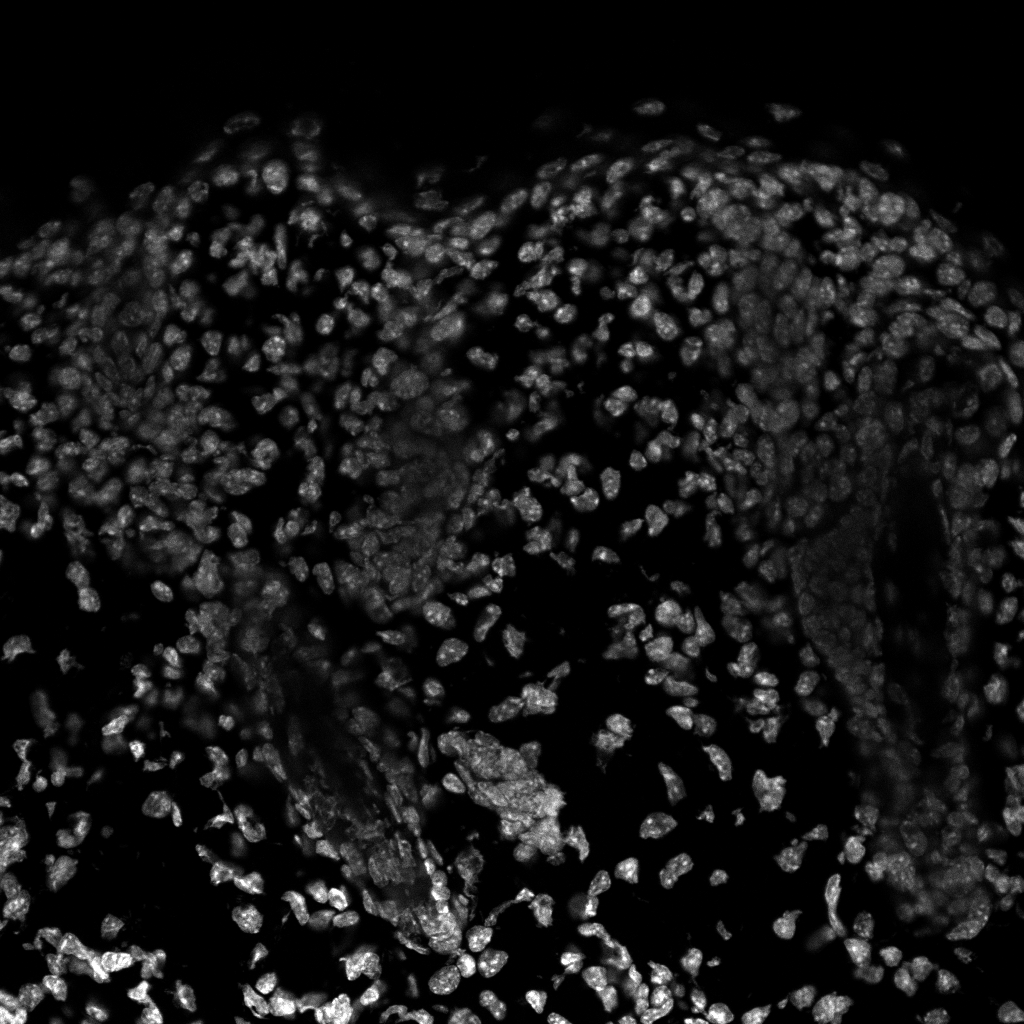

Supplement: Supplementary file 6 — Source Data Fig. 5 [file 44318_2024_39_MOESM6_ESM.zip › Figure 5/5B/P2 Saline F480green CD206red MMP9gray 40x1.tif]

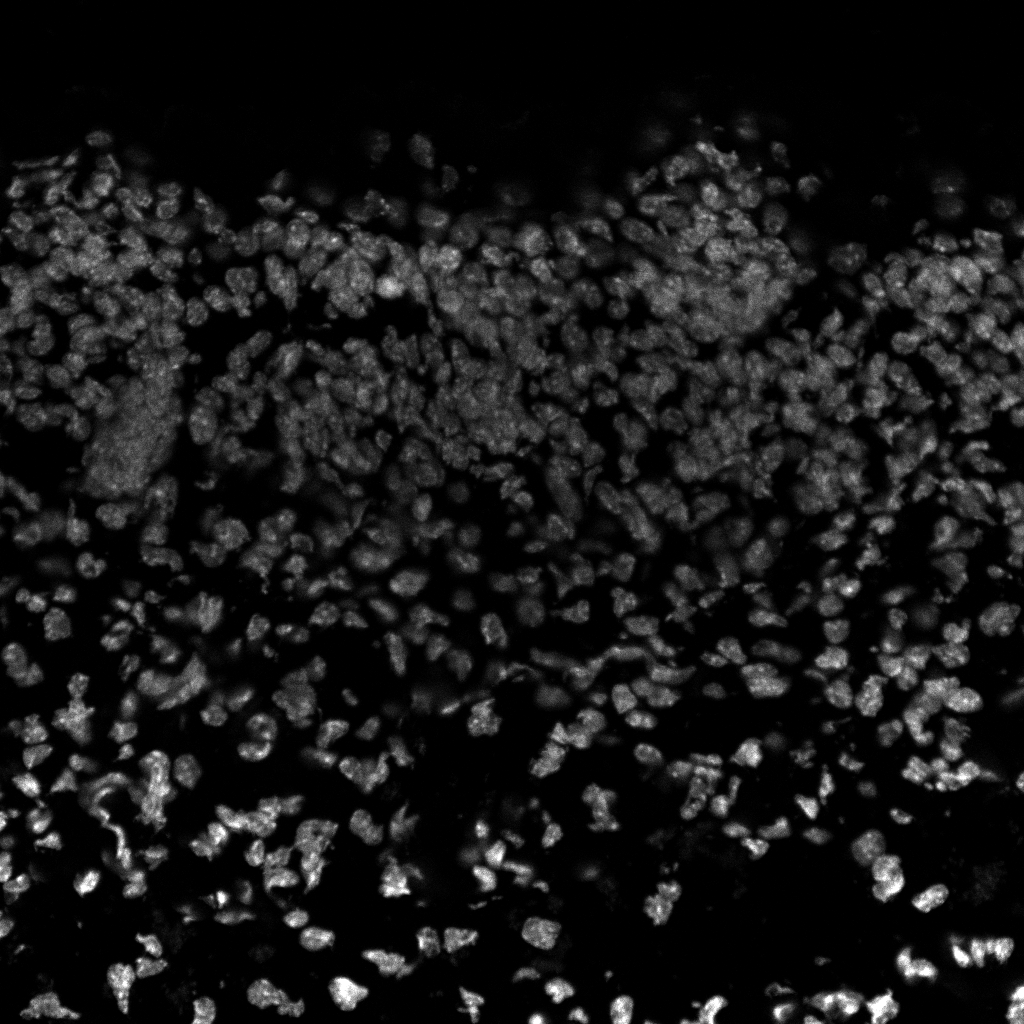

Supplement: Supplementary file 6 — Source Data Fig. 5 [file 44318_2024_39_MOESM6_ESM.zip › Figure 5/5D/Syro Treated Beta1 Lam332 Expression/E18.5 KO DMSO B1green LAM332red 40X.tif]

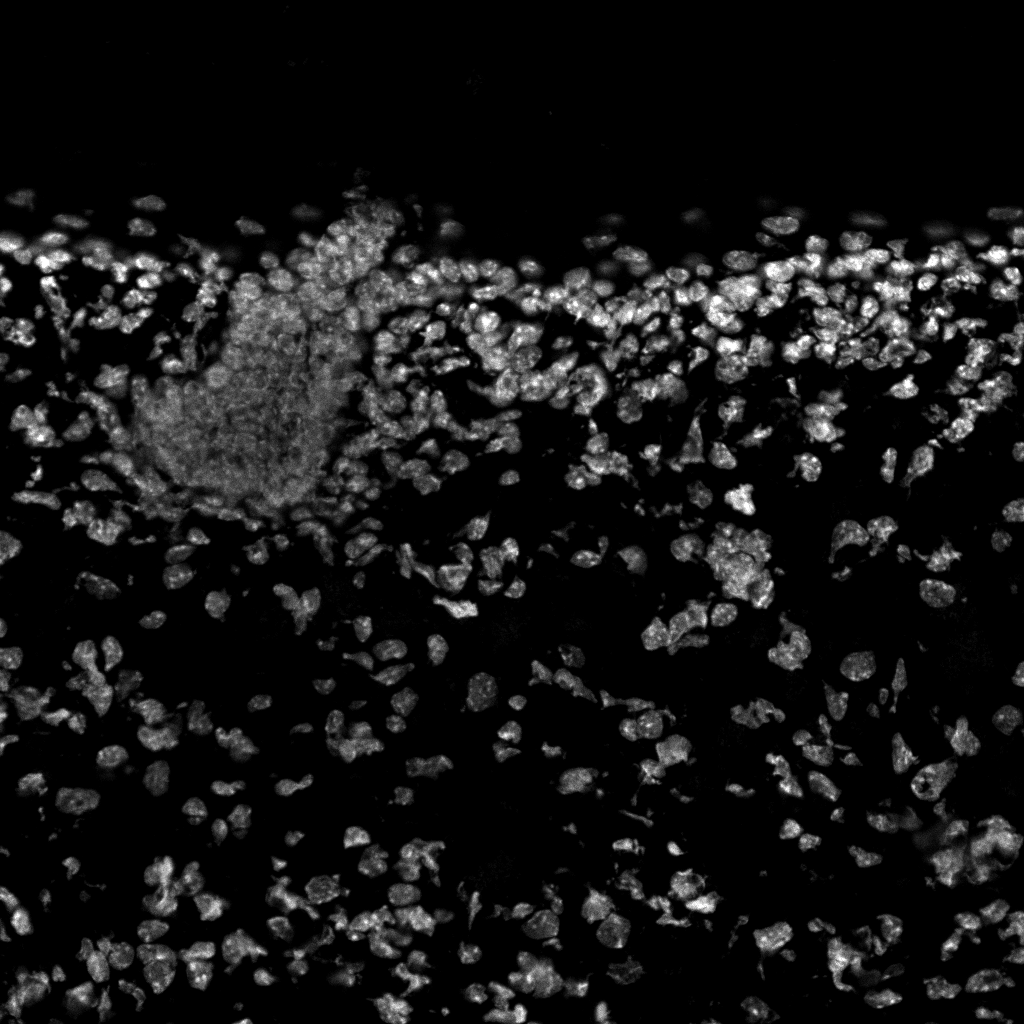

Supplement: Supplementary file 6 — Source Data Fig. 5 [file 44318_2024_39_MOESM6_ESM.zip › Figure 5/5D/Syro Treated Beta1 Lam332 Expression/E18.5 KO SYRO B1green LAM332red 40X.tif]

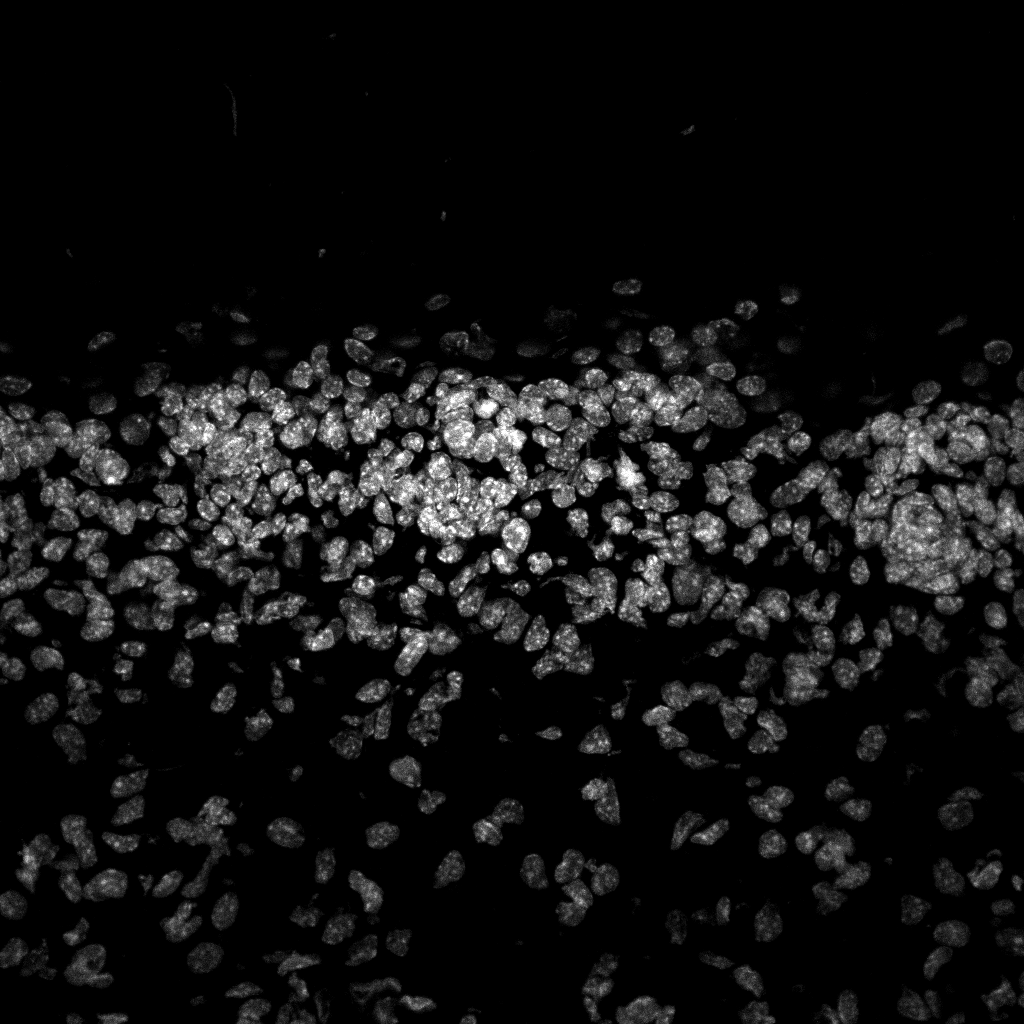

Supplement: Supplementary file 6 — Source Data Fig. 5 [file 44318_2024_39_MOESM6_ESM.zip › Figure 5/5D/AZD Treated MMP9 F480 Expression/AZD3965 KO F480green MMP9red 40X.tif]

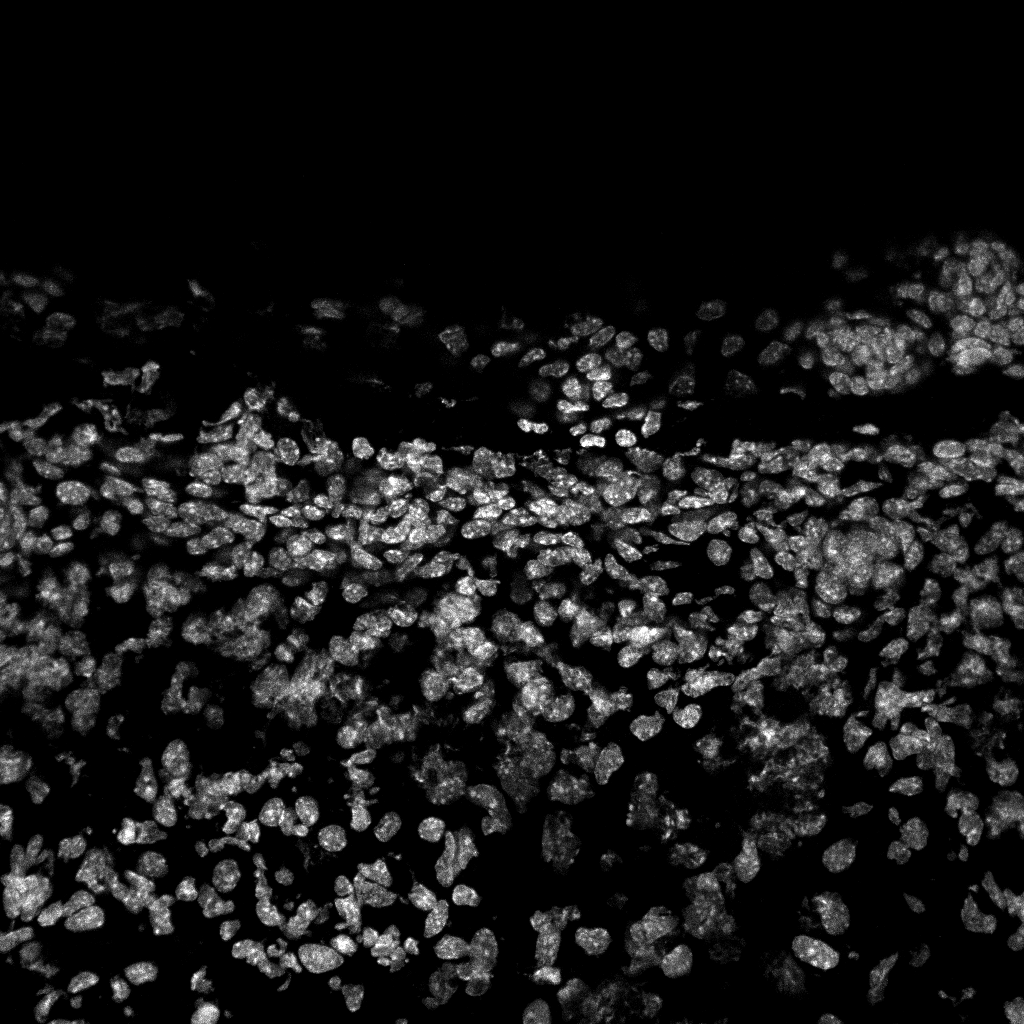

Supplement: Supplementary file 6 — Source Data Fig. 5 [file 44318_2024_39_MOESM6_ESM.zip › Figure 5/5D/AZD Treated MMP9 F480 Expression/DMSO KO F480green MMP9red 40X.tif]

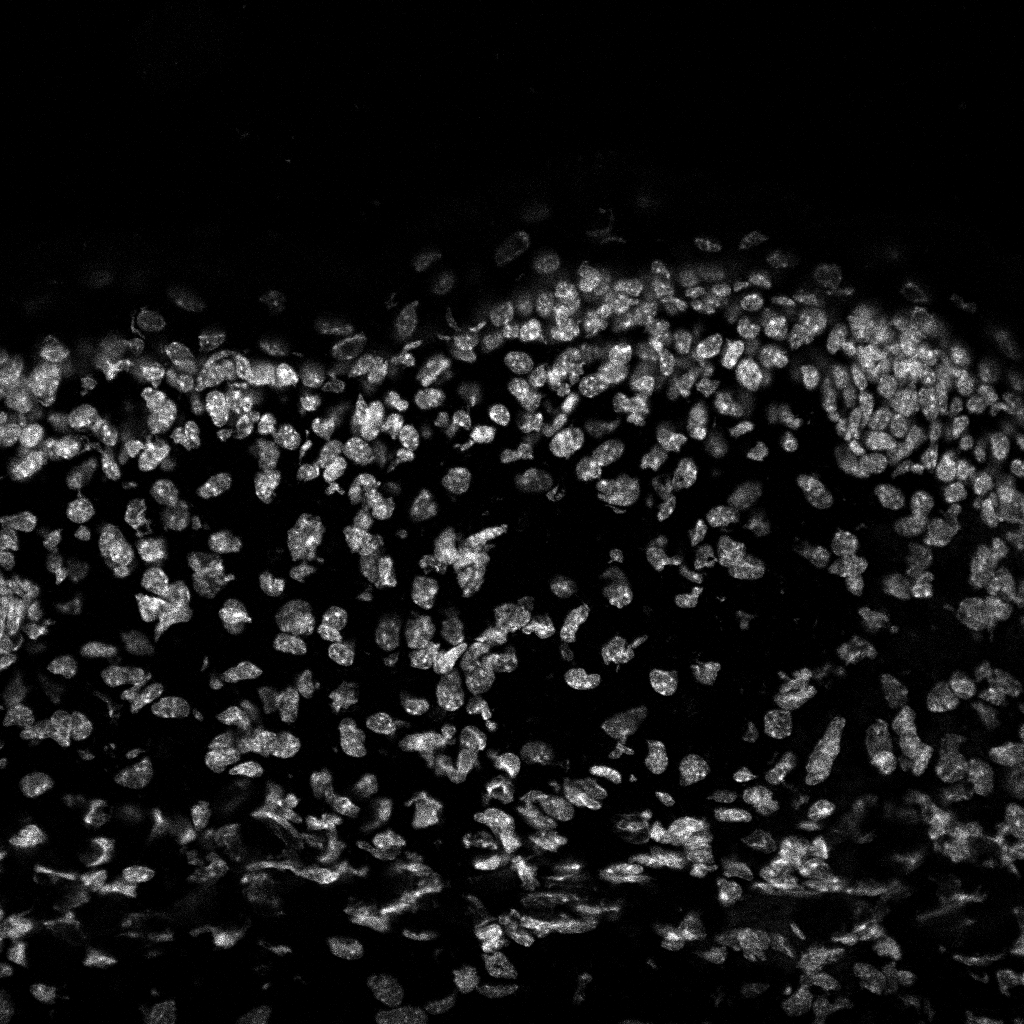

Supplement: Supplementary file 6 — Source Data Fig. 5 [file 44318_2024_39_MOESM6_ESM.zip › Figure 5/5D/AZD Treated Beta1 Lam332 Expression/AZD3965 KO1 BETA1green LAM332red 40X.tif]

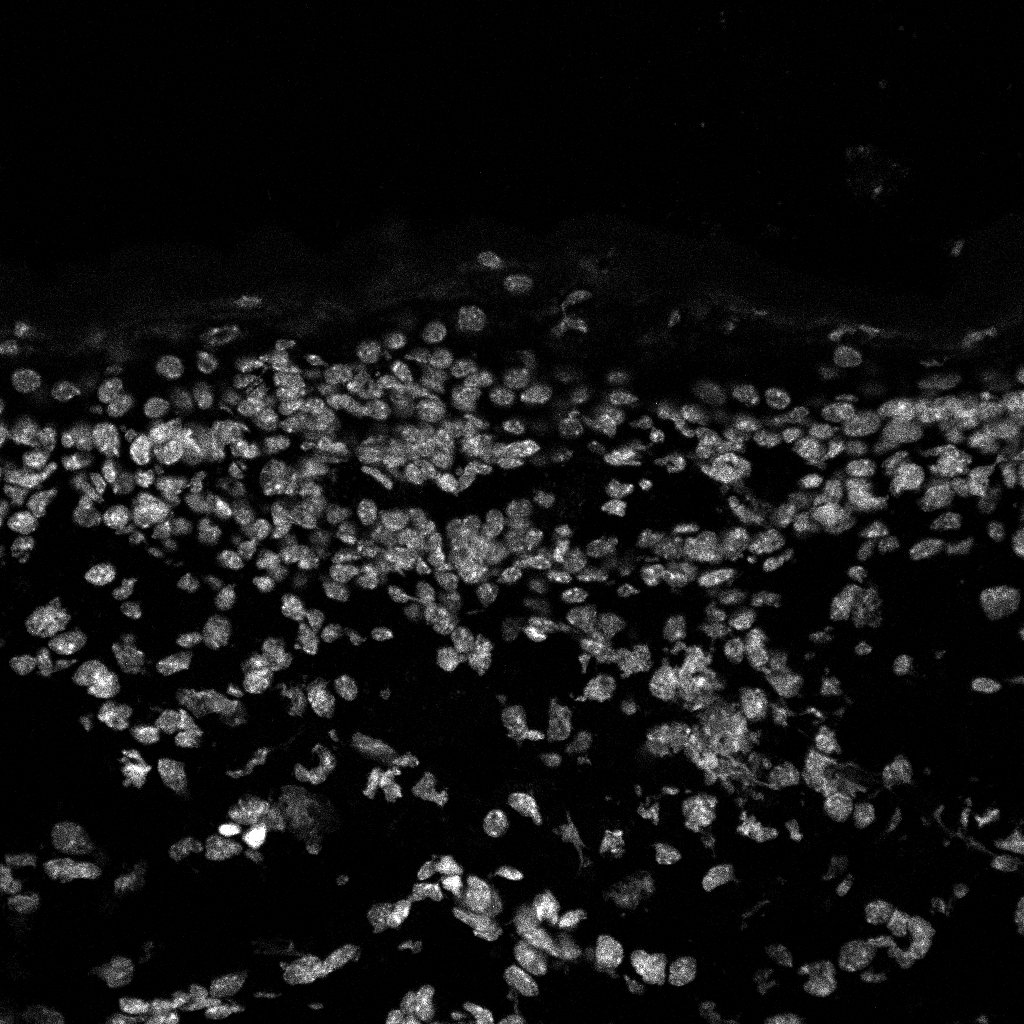

Supplement: Supplementary file 6 — Source Data Fig. 5 [file 44318_2024_39_MOESM6_ESM.zip › Figure 5/5D/AZD Treated Beta1 Lam332 Expression/DMSO KO BETA1green LAM332red 40X.tif]

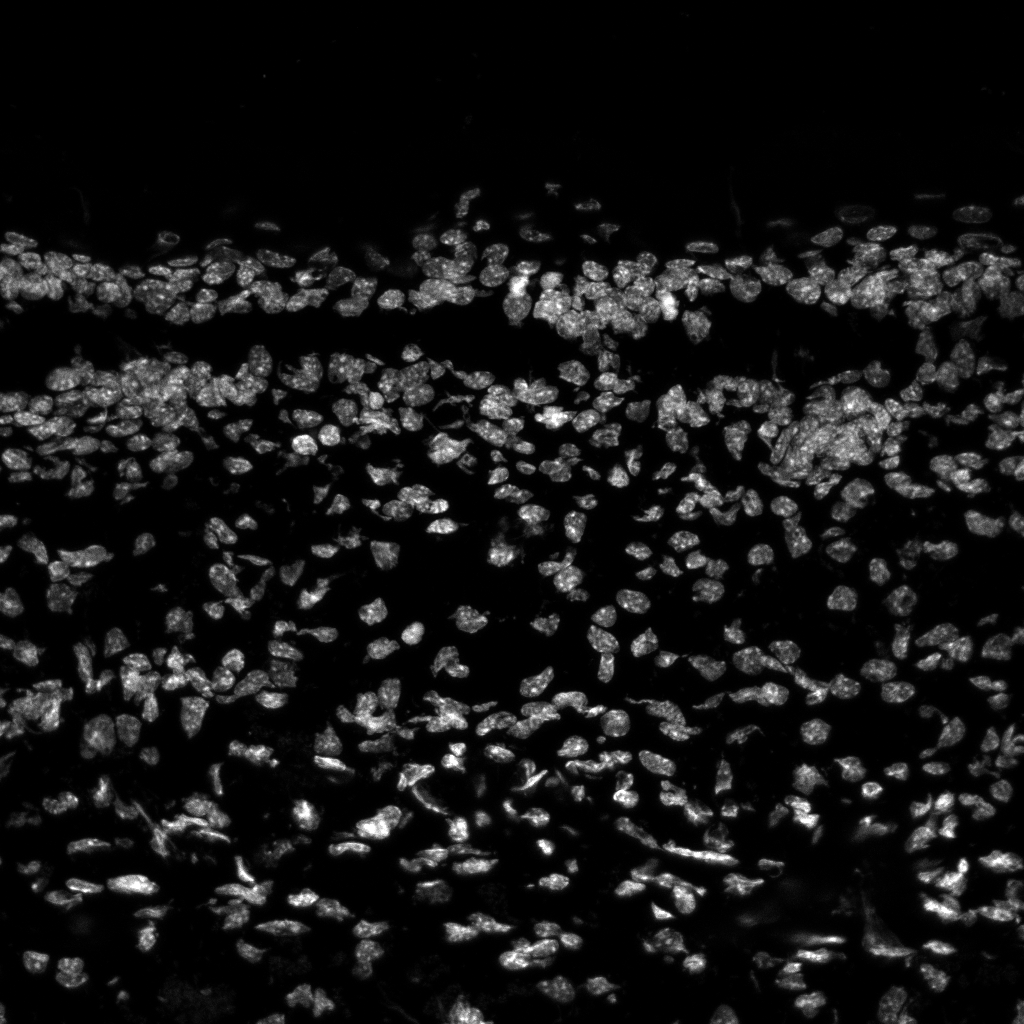

Supplement: Supplementary file 6 — Source Data Fig. 5 [file 44318_2024_39_MOESM6_ESM.zip › Figure 5/5D/Syro Treated MMP9 F480 Expression/MAX_E18.5 DMSO KO MMP9red F480green 40X.tif]

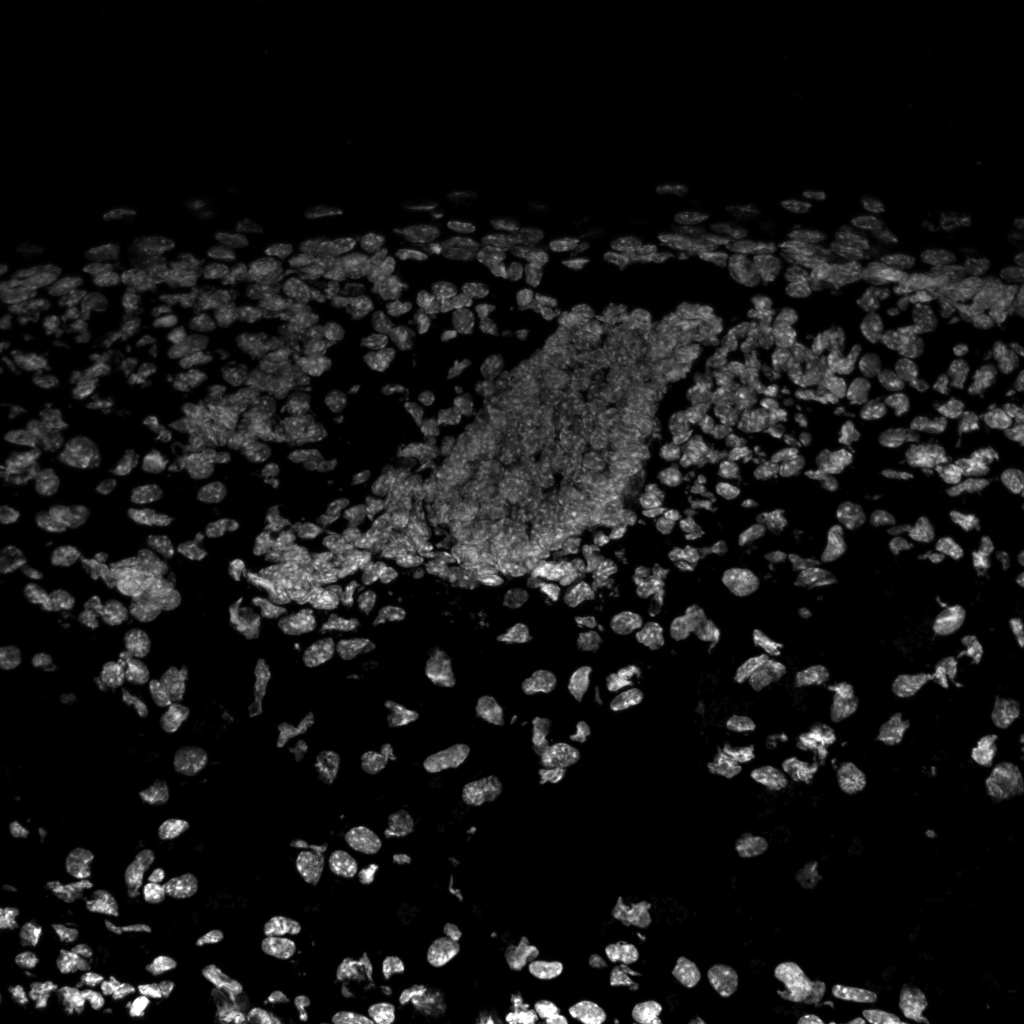

Supplement: Supplementary file 6 — Source Data Fig. 5 [file 44318_2024_39_MOESM6_ESM.zip › Figure 5/5D/Syro Treated MMP9 F480 Expression/MAX_E18.5 SYRO KO MMP9red F480green 40X.tif]

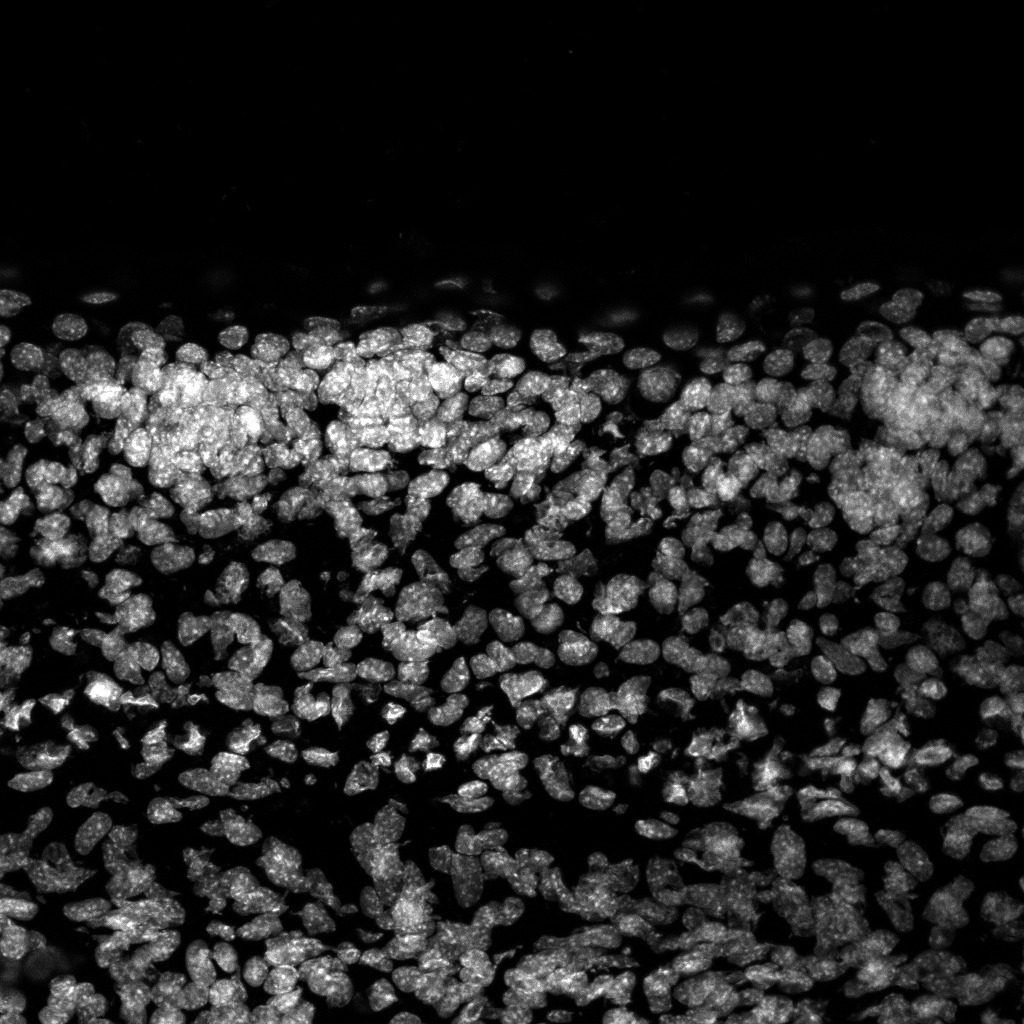

Supplement: Supplementary file 7 — Source Data Fig. 6 [file 44318_2024_39_MOESM7_ESM.zip › Figure 6/6A/F480 MMP9 staining/E18.5 PBS KO F480green MMP9red 40X.tif]

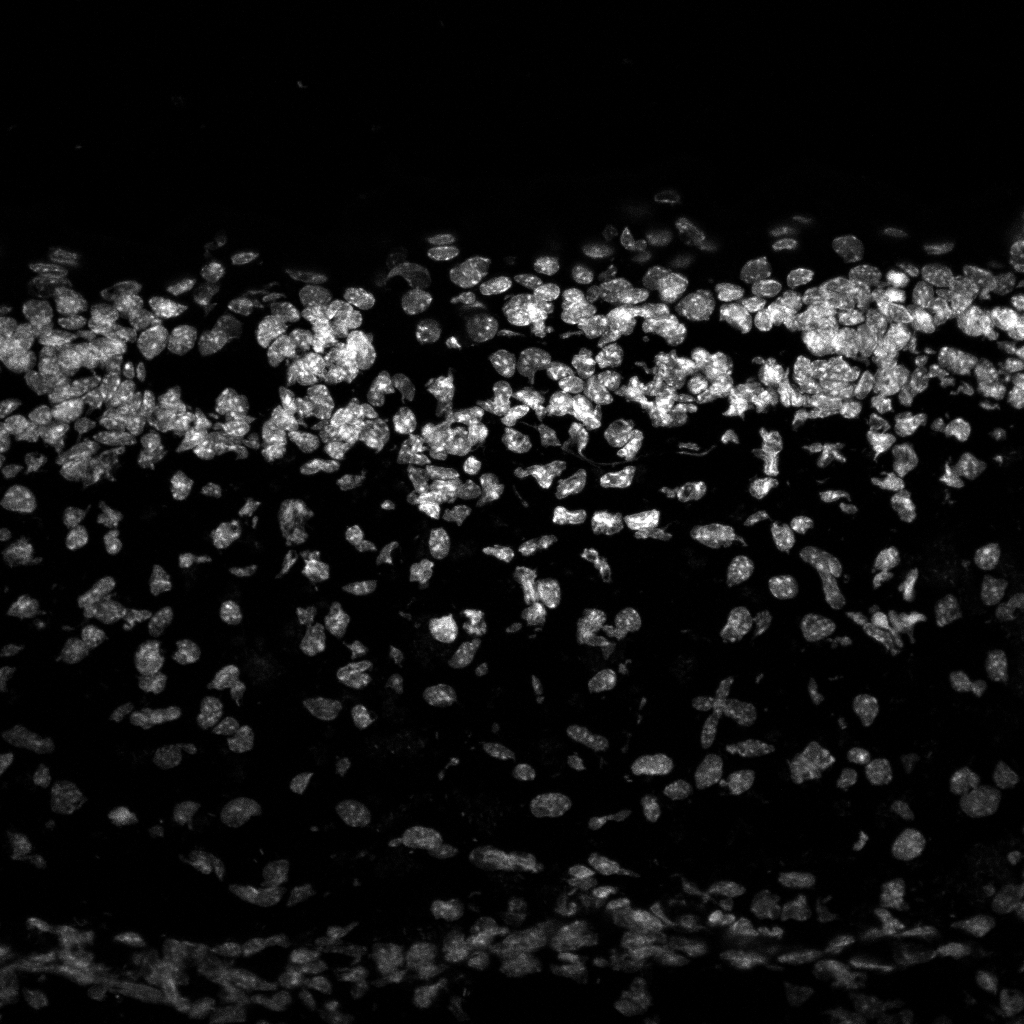

Supplement: Supplementary file 7 — Source Data Fig. 6 [file 44318_2024_39_MOESM7_ESM.zip › Figure 6/6A/F480 MMP9 staining/E18.5 2DG KO F480green MMP9red 40X.tif]

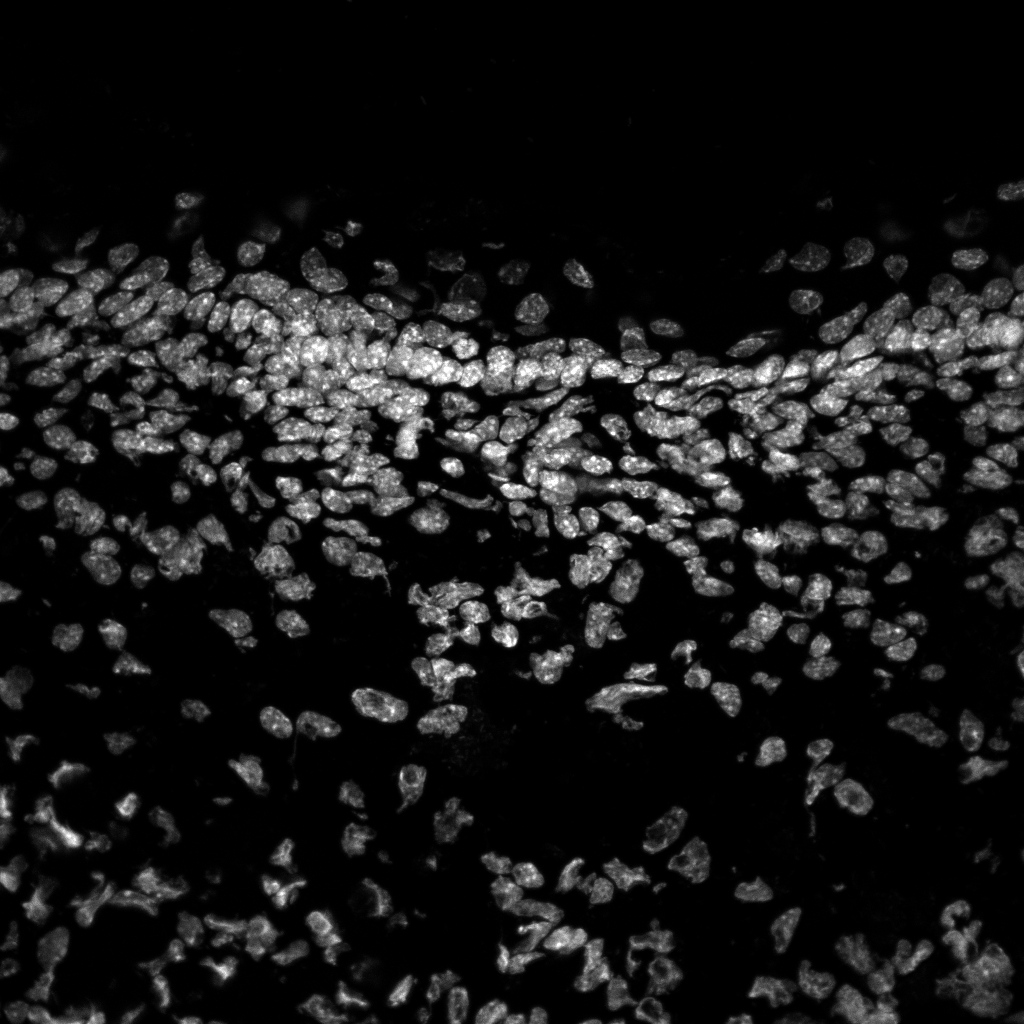

Supplement: Supplementary file 7 — Source Data Fig. 6 [file 44318_2024_39_MOESM7_ESM.zip › Figure 6/6A/F480 MMP9 staining/E18.5 PA KO F480green MMP9red 40X.tif]

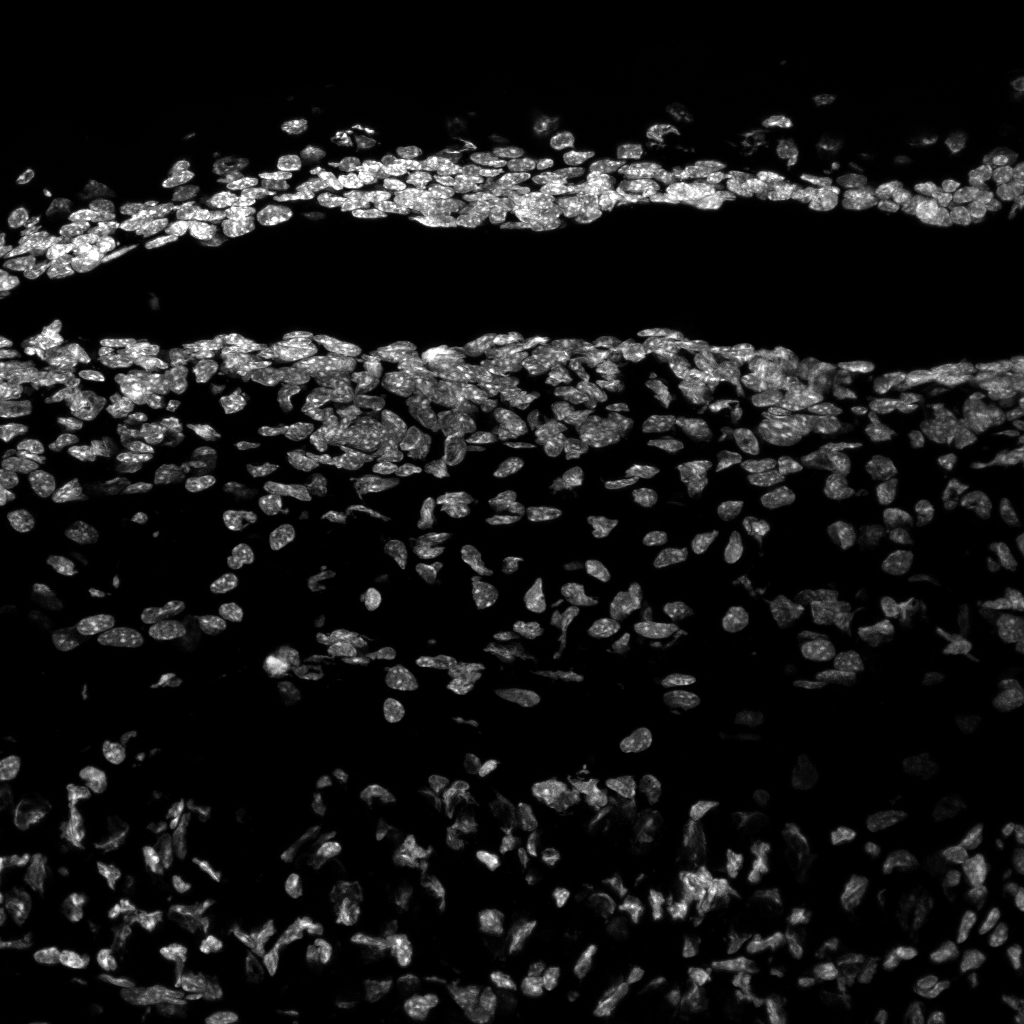

Supplement: Supplementary file 7 — Source Data Fig. 6 [file 44318_2024_39_MOESM7_ESM.zip › Figure 6/6A/BETA1 LAM332 staining/E18.5 2DG KO B1green LAM332red 40X.tif]

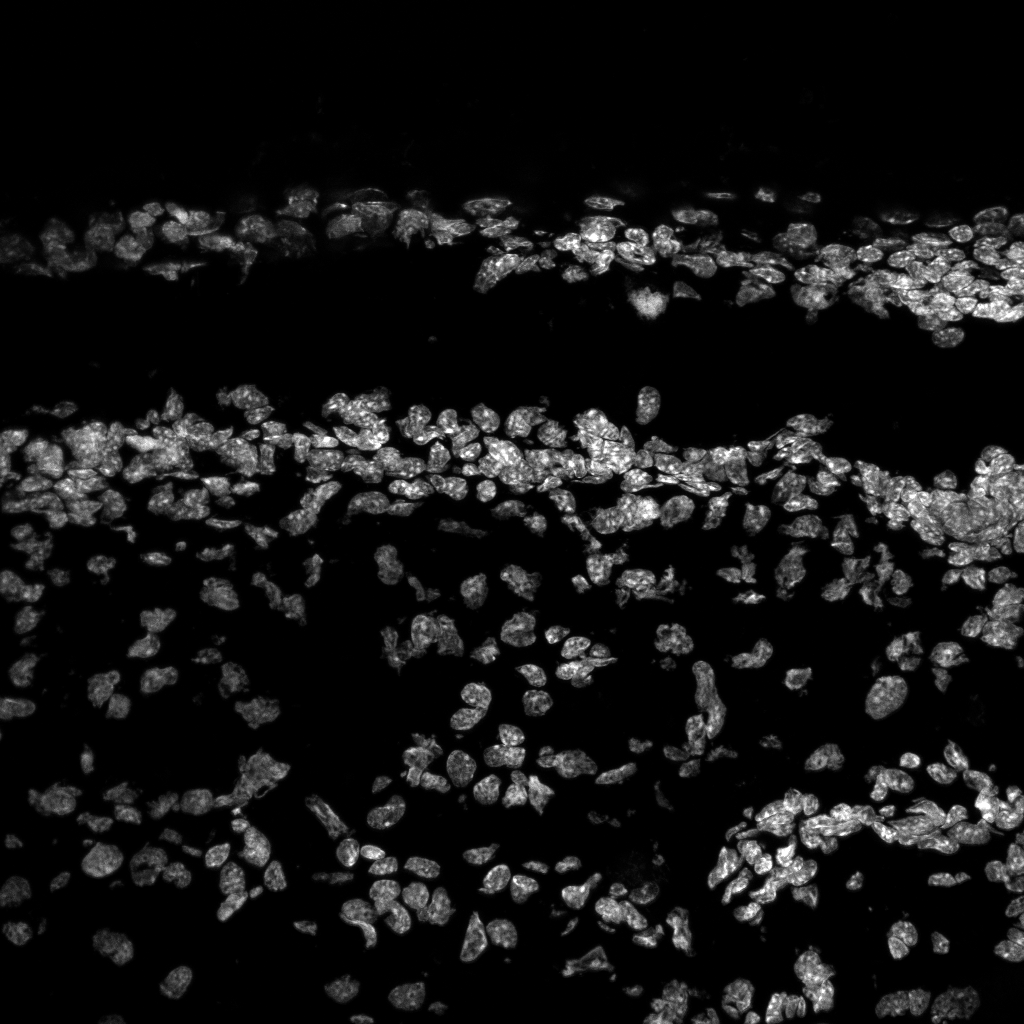

Supplement: Supplementary file 7 — Source Data Fig. 6 [file 44318_2024_39_MOESM7_ESM.zip › Figure 6/6A/BETA1 LAM332 staining/E18.5 PBS KO B1green LAM332red 40X.tif]

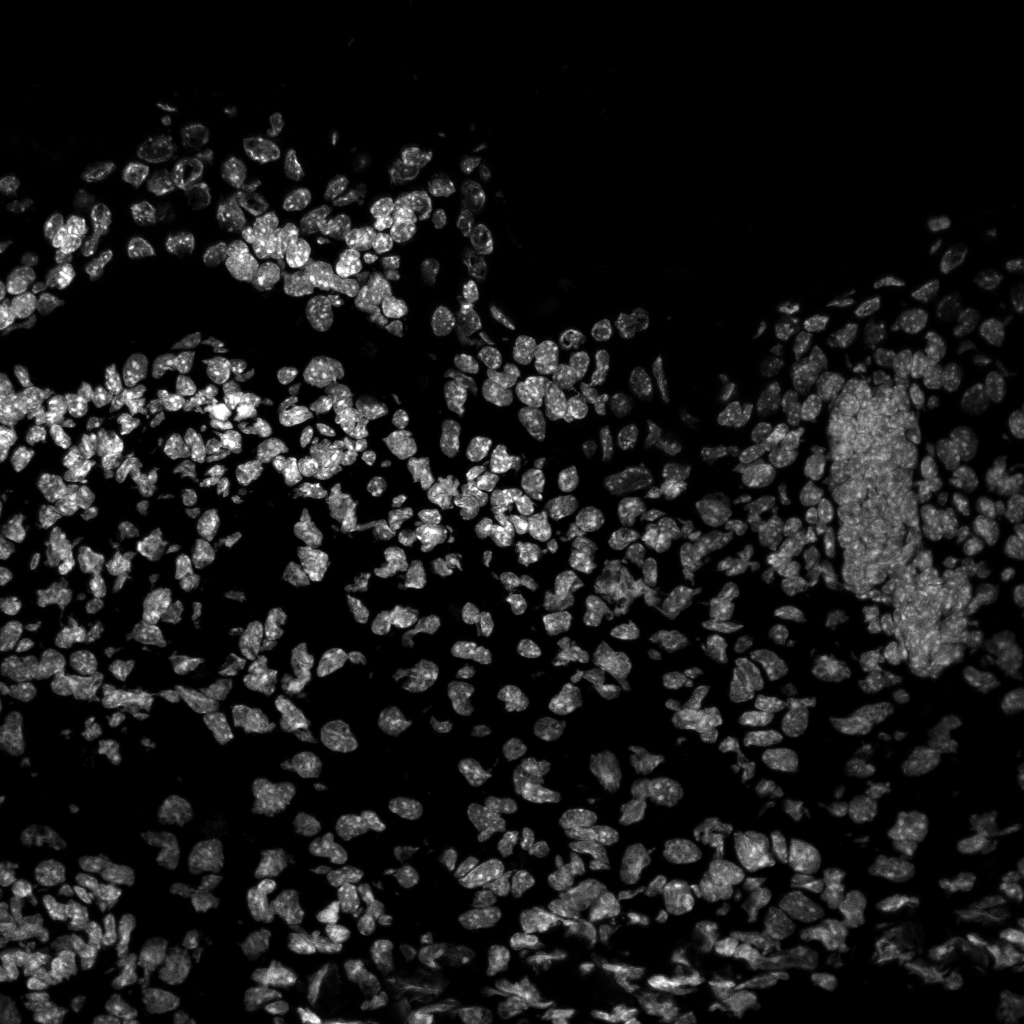

Supplement: Supplementary file 7 — Source Data Fig. 6 [file 44318_2024_39_MOESM7_ESM.zip › Figure 6/6A/BETA1 LAM332 staining/E18.5 PA KO B1green LAM5red 40X.tif]

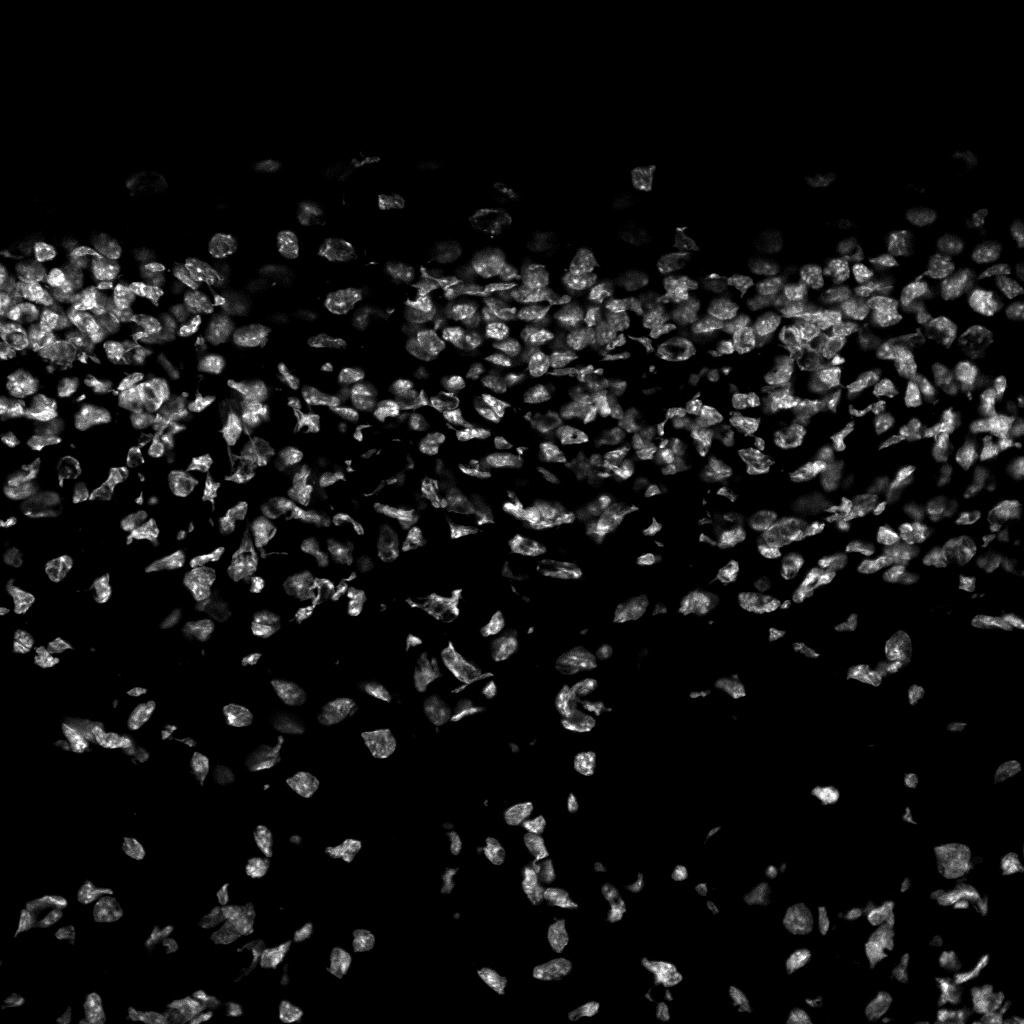

Supplement: Supplementary file 7 — Source Data Fig. 6 [file 44318_2024_39_MOESM7_ESM.zip › Figure 6/6D/NAC BETA1 LAM332 staining/E18.5 KO PBS B1green LAM332red 40X.tif]

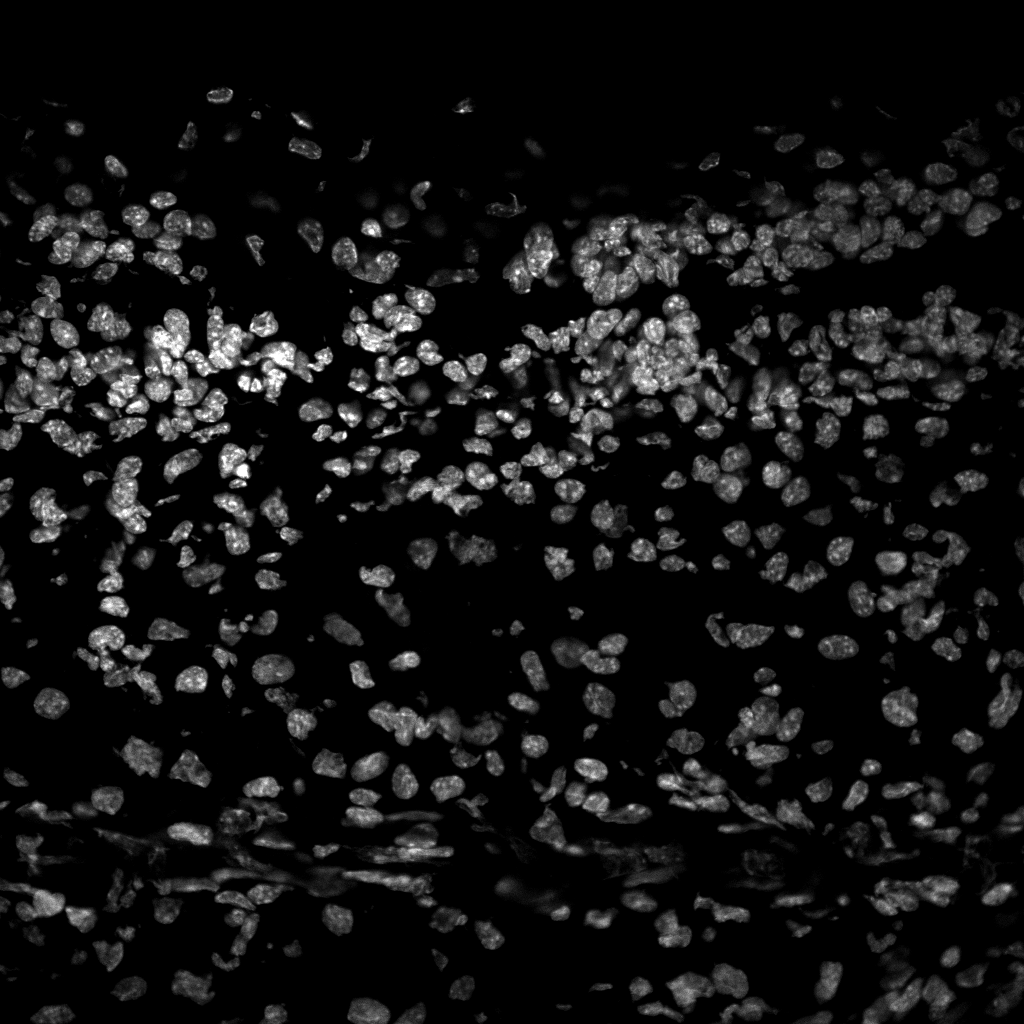

Supplement: Supplementary file 7 — Source Data Fig. 6 [file 44318_2024_39_MOESM7_ESM.zip › Figure 6/6D/NAC BETA1 LAM332 staining/E18.5 KO NAC B1green LAM332red 40X.tif]

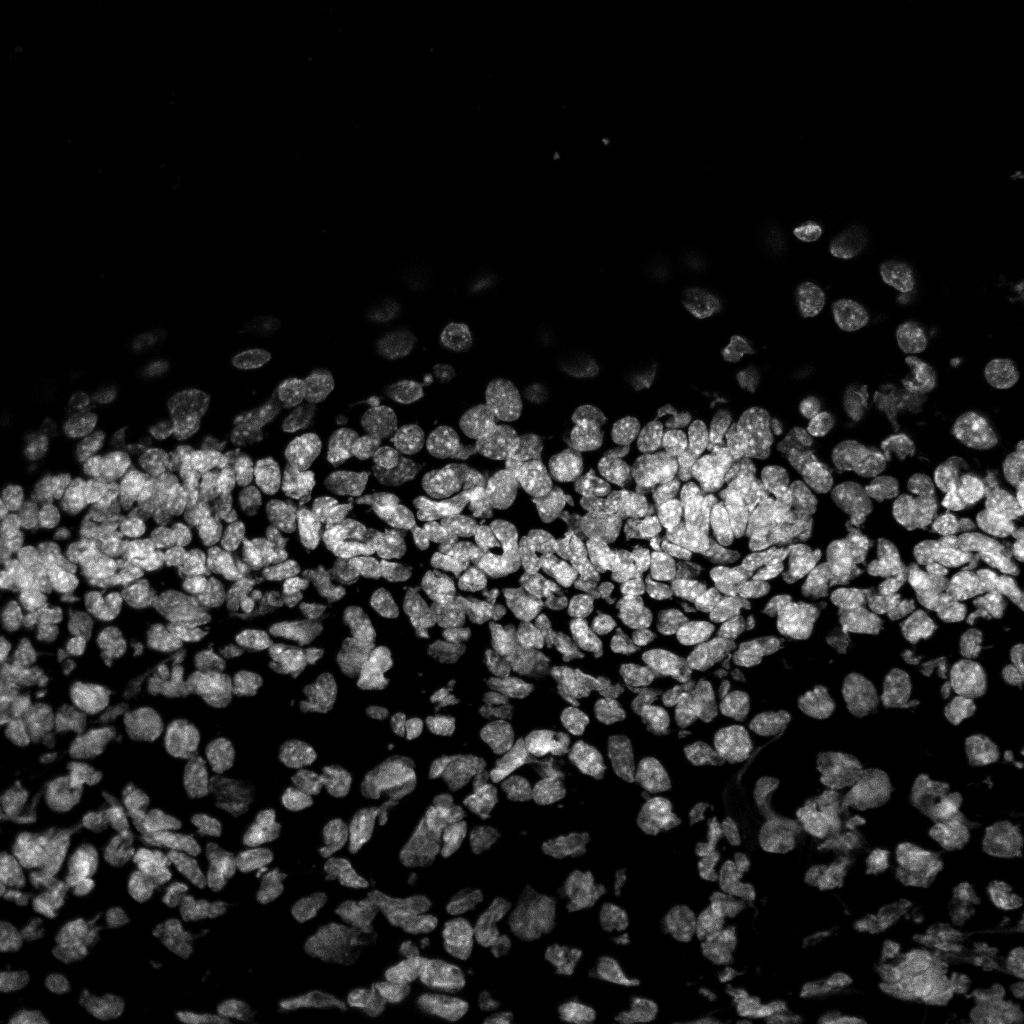

Supplement: Supplementary file 7 — Source Data Fig. 6 [file 44318_2024_39_MOESM7_ESM.zip › Figure 6/6D/Chetomin F480 MMP9 staining/Chetomin KO F480green MMP9red 40X.tif]

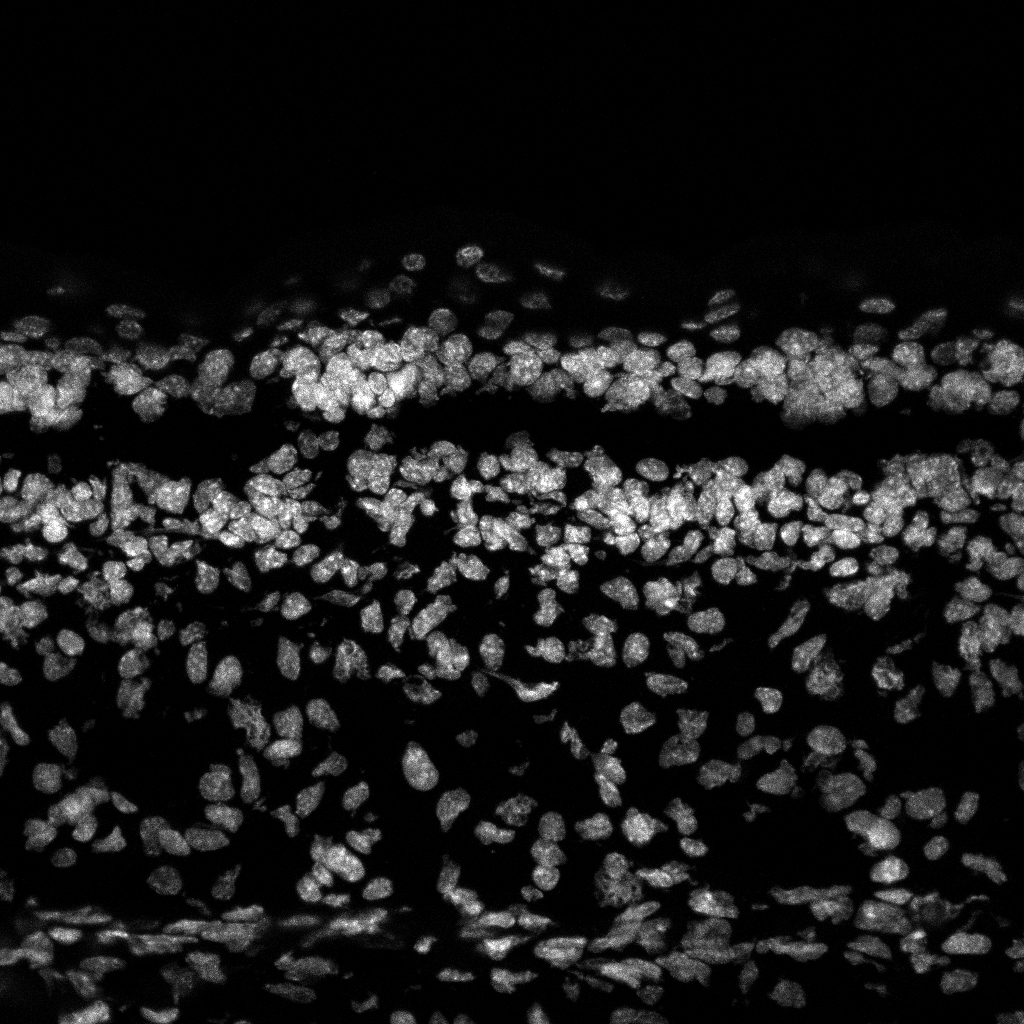

Supplement: Supplementary file 7 — Source Data Fig. 6 [file 44318_2024_39_MOESM7_ESM.zip › Figure 6/6D/Chetomin F480 MMP9 staining/DMSO KO F480green MMP9red 40X.tif]

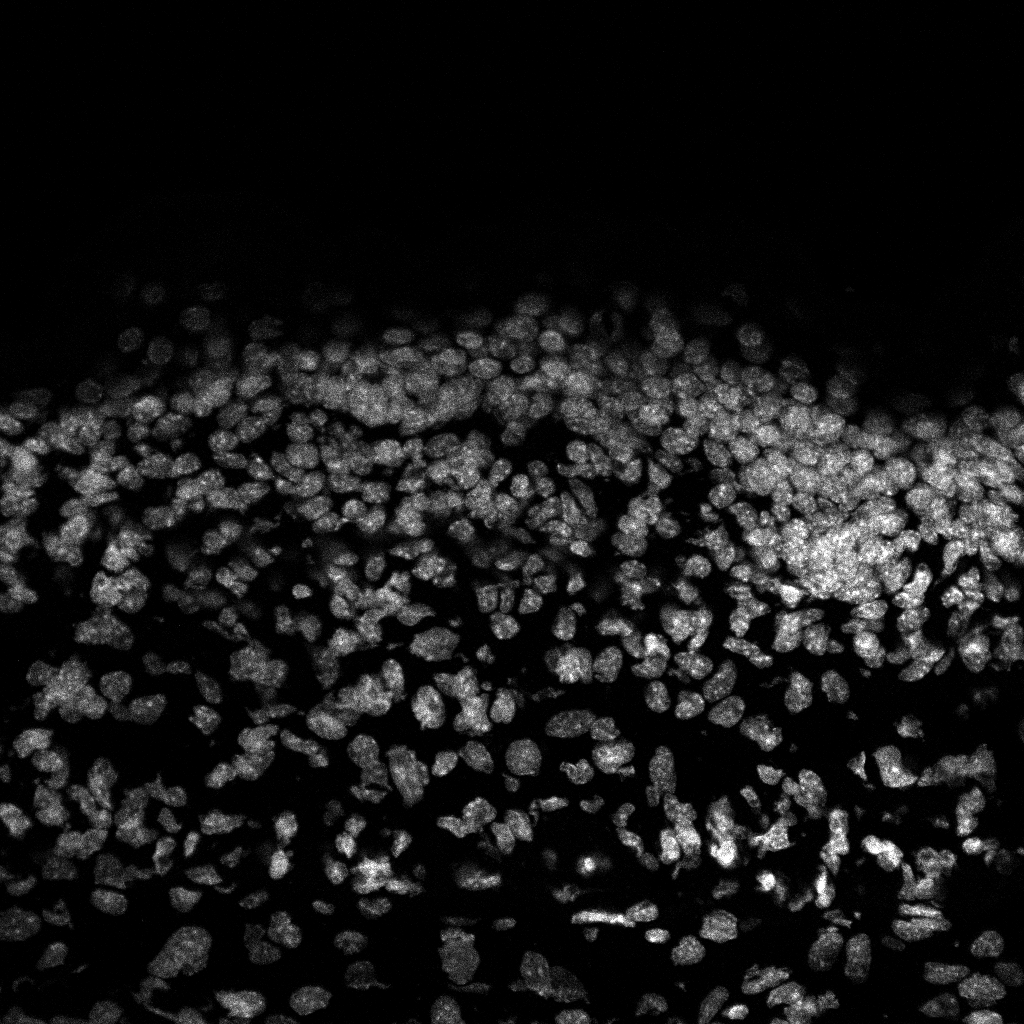

Supplement: Supplementary file 7 — Source Data Fig. 6 [file 44318_2024_39_MOESM7_ESM.zip › Figure 6/6D/Chetomin BETA1 LAM332 staining/DMSO KO BETA1green LAM332red 40X.tif]

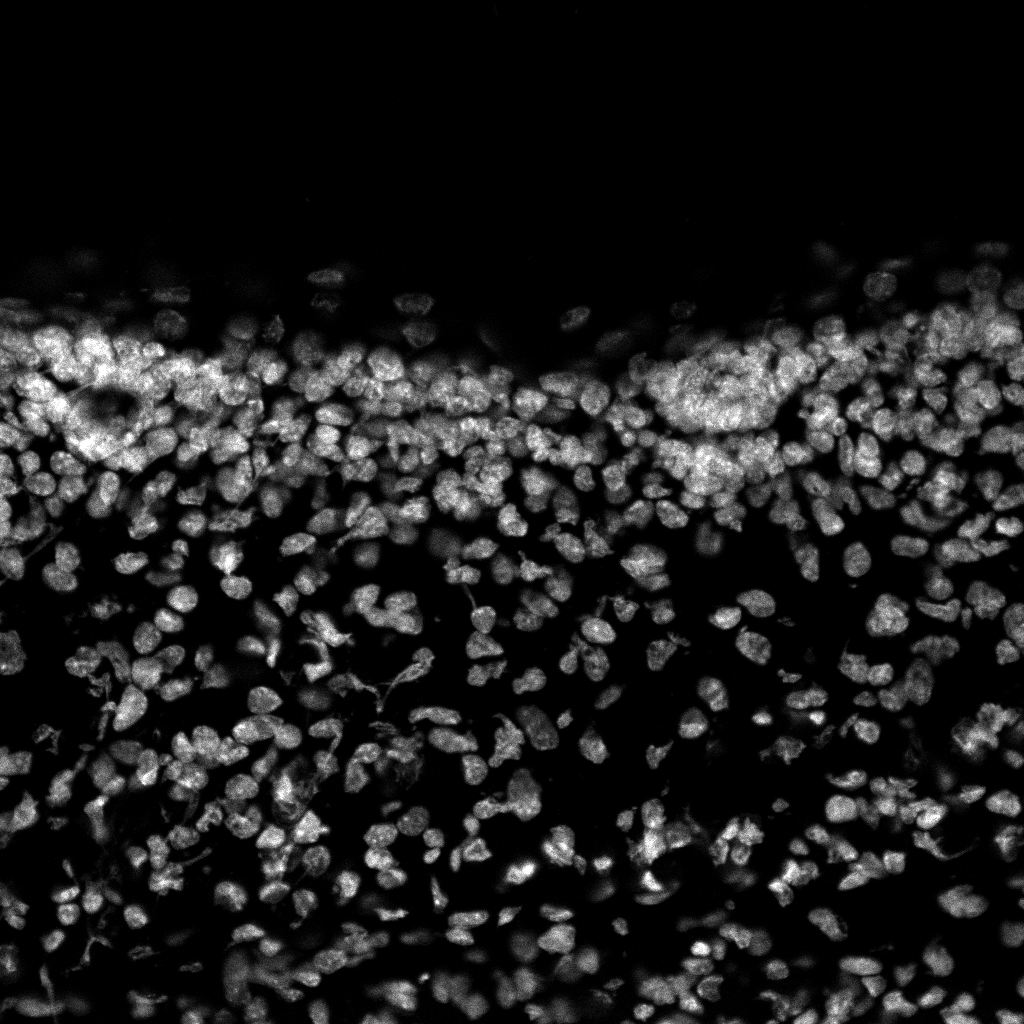

Supplement: Supplementary file 7 — Source Data Fig. 6 [file 44318_2024_39_MOESM7_ESM.zip › Figure 6/6D/Chetomin BETA1 LAM332 staining/Chetomin KO BETA1green LAM332red 40X.tif]

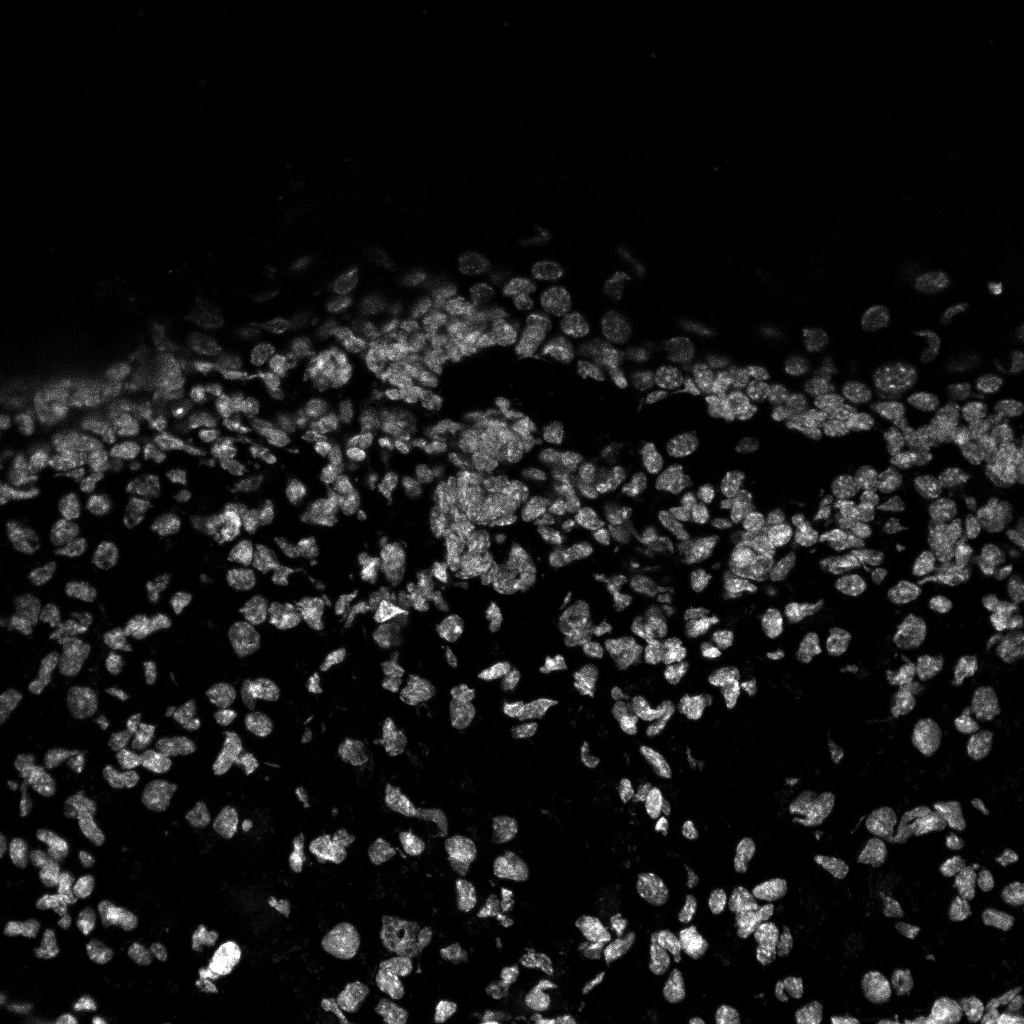

Supplement: Supplementary file 7 — Source Data Fig. 6 [file 44318_2024_39_MOESM7_ESM.zip › Figure 6/6D/NAC F480 MMP9 staining/E18.5 PBS KO F480green MMP9red 40X.tif]

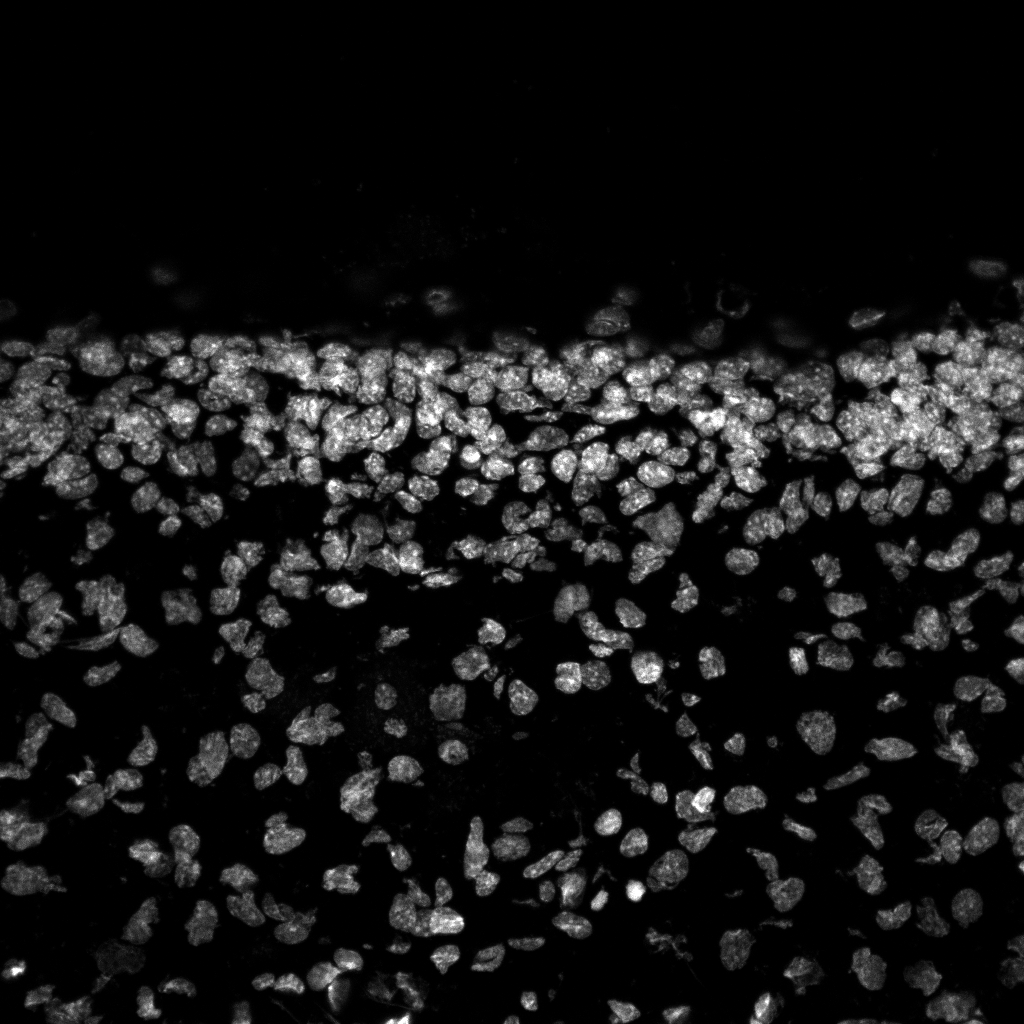

Supplement: Supplementary file 7 — Source Data Fig. 6 [file 44318_2024_39_MOESM7_ESM.zip › Figure 6/6D/NAC F480 MMP9 staining/E18.5 NAC KO F480green MMP9red 40X.tif]

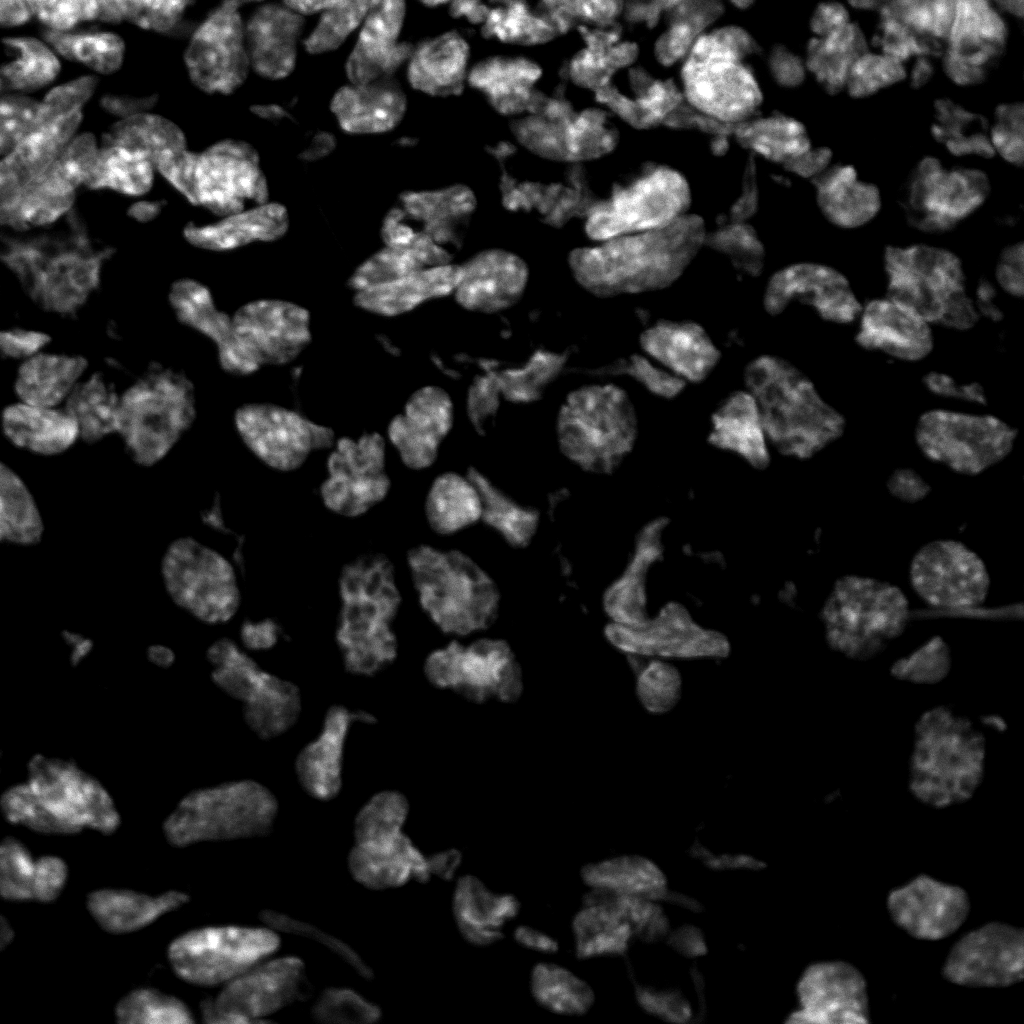

Supplement: Supplementary file 8 — Source Data Fig. 7 [file 44318_2024_39_MOESM8_ESM.zip › Figure 7/7B/Chetomin KO F480green p65NFKBred 40X 2.41X]

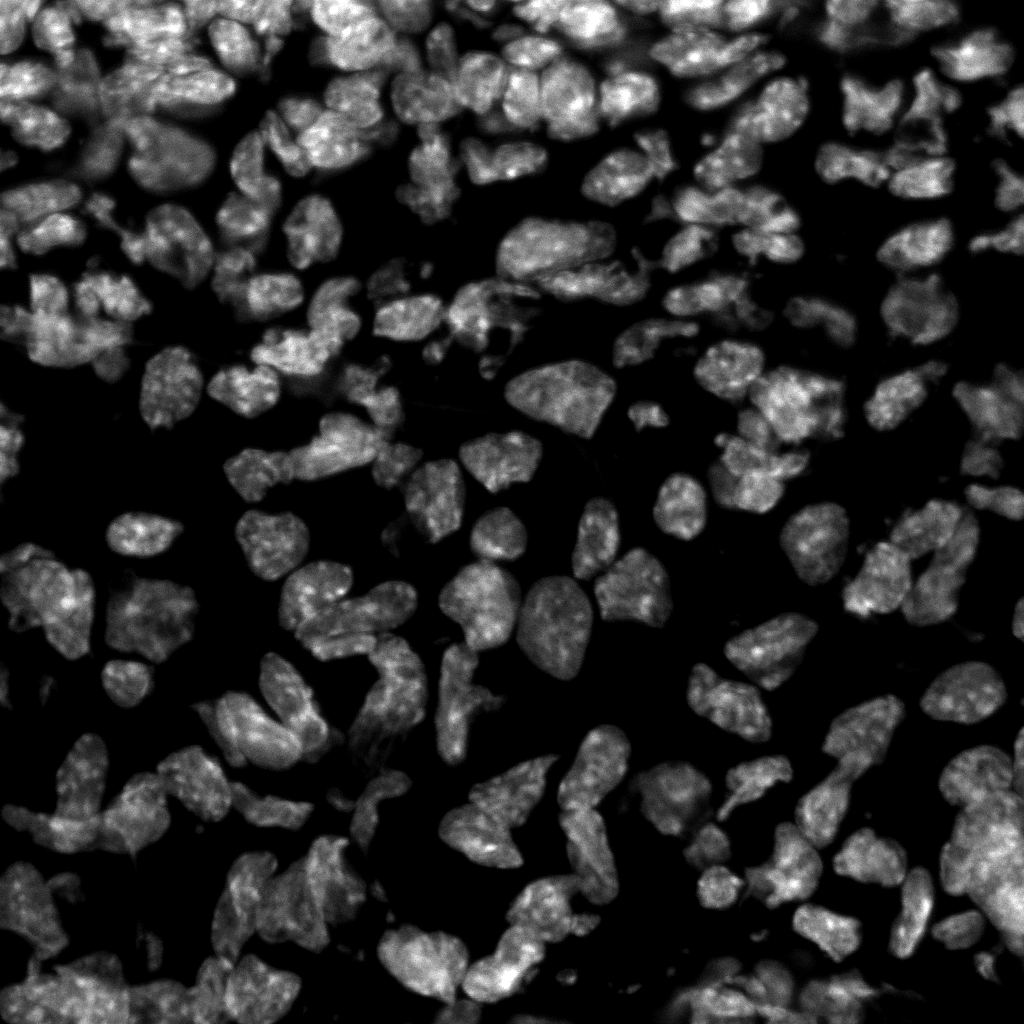

Supplement: Supplementary file 8 — Source Data Fig. 7 [file 44318_2024_39_MOESM8_ESM.zip › Figure 7/7B/DMSO KO F480green p65NFKBred 40X 2.41X]

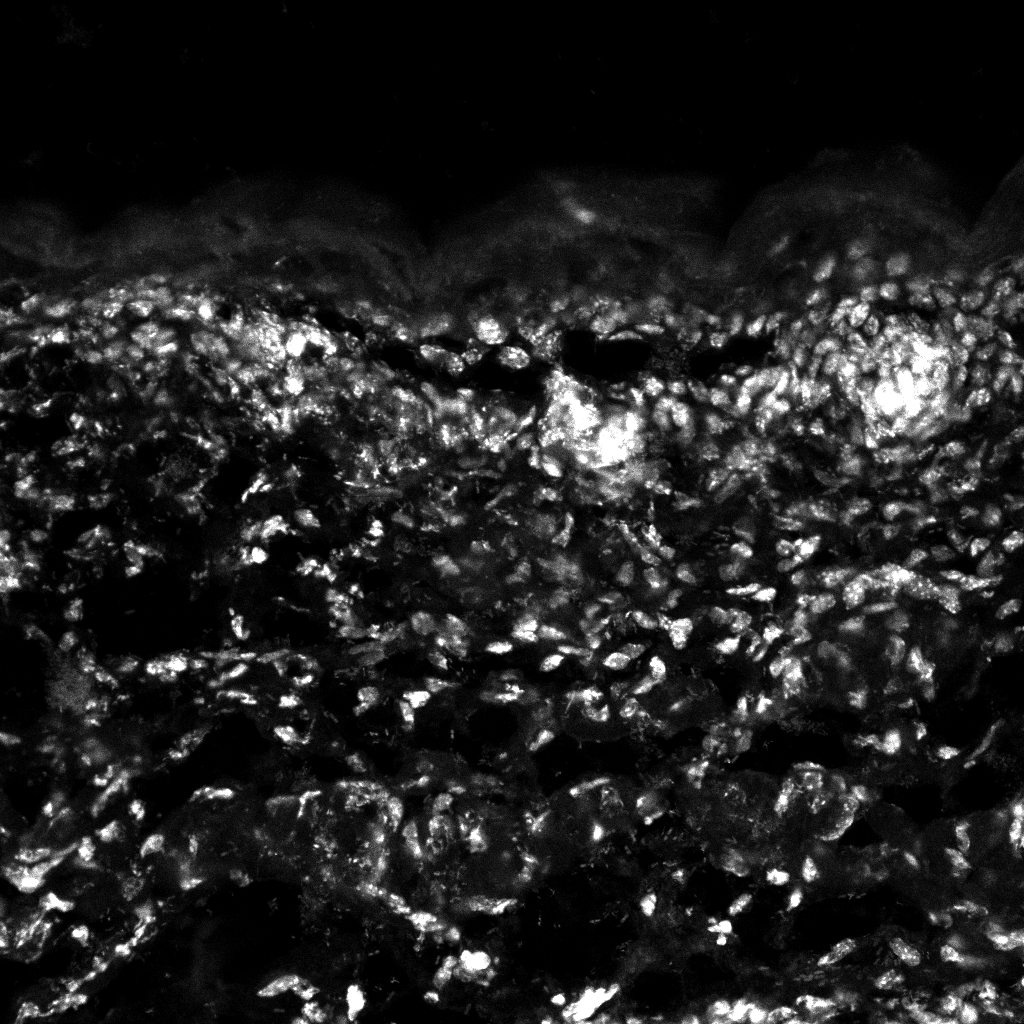

Supplement: Supplementary file 8 — Source Data Fig. 7 [file 44318_2024_39_MOESM8_ESM.zip › Figure 7/7E/DAPI-Vehicle treated cKO p65NFKBgreen MMP9red 40X.tif]

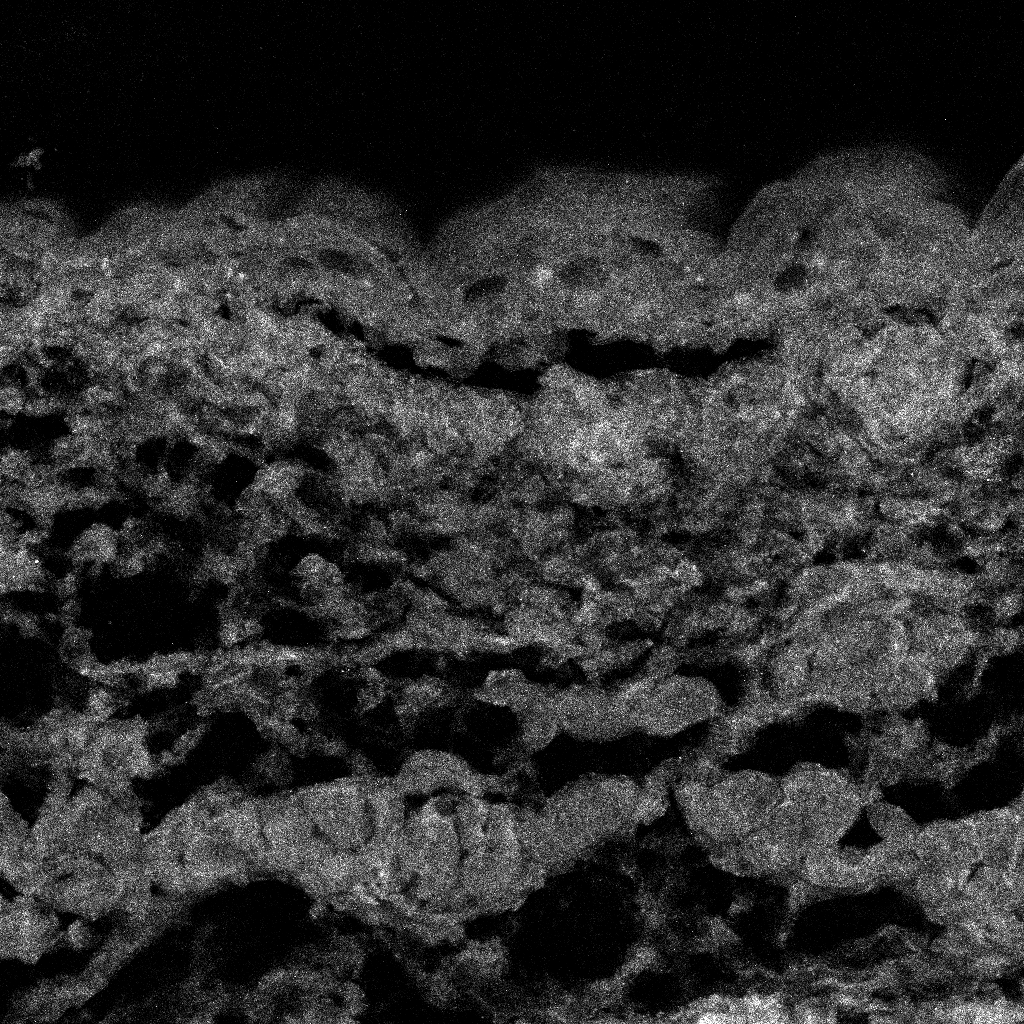

Supplement: Supplementary file 8 — Source Data Fig. 7 [file 44318_2024_39_MOESM8_ESM.zip › Figure 7/7E/P65NFKB-Vehicle treated cKO p65NFKBgreen MMP9red 40X.tif]

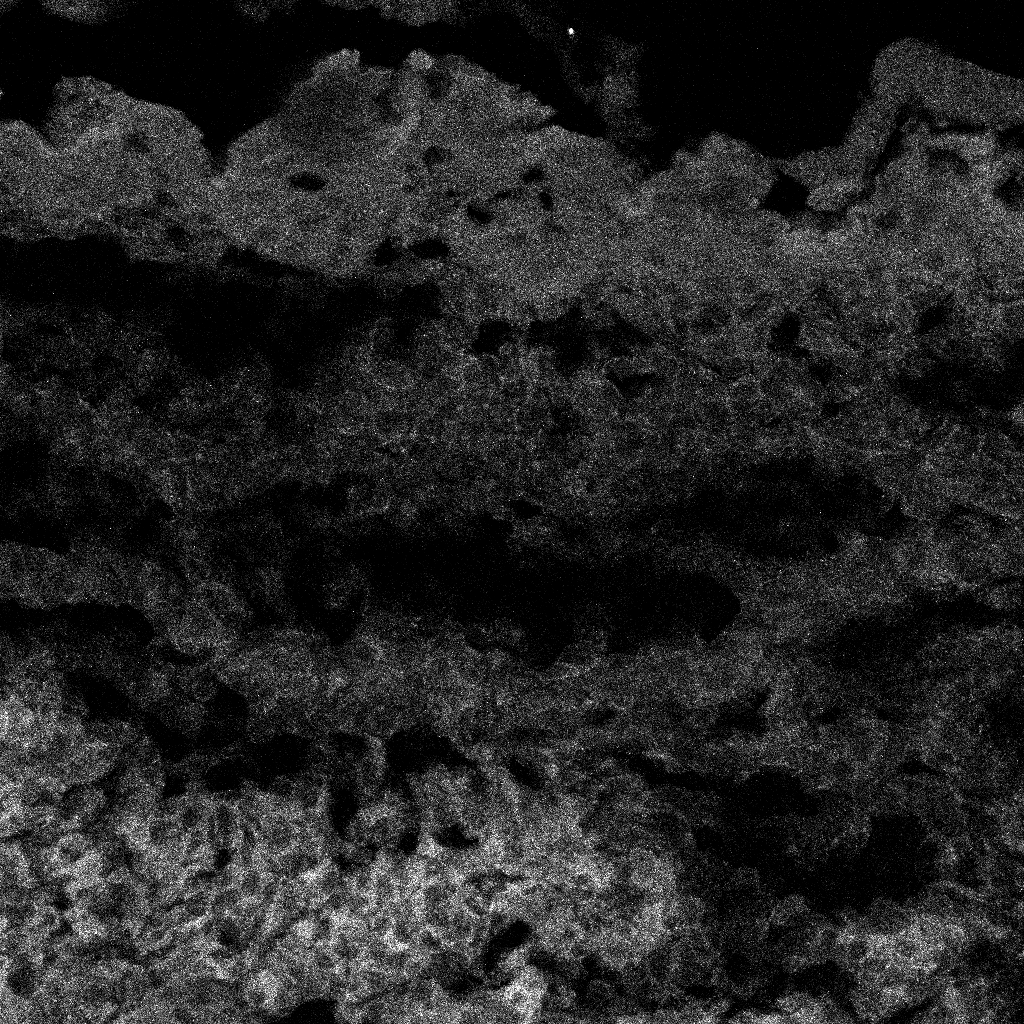

Supplement: Supplementary file 8 — Source Data Fig. 7 [file 44318_2024_39_MOESM8_ESM.zip › Figure 7/7E/P65NFKB-BAY11 treated cKO p65NFKBgreen MMP9red 40X.tif]

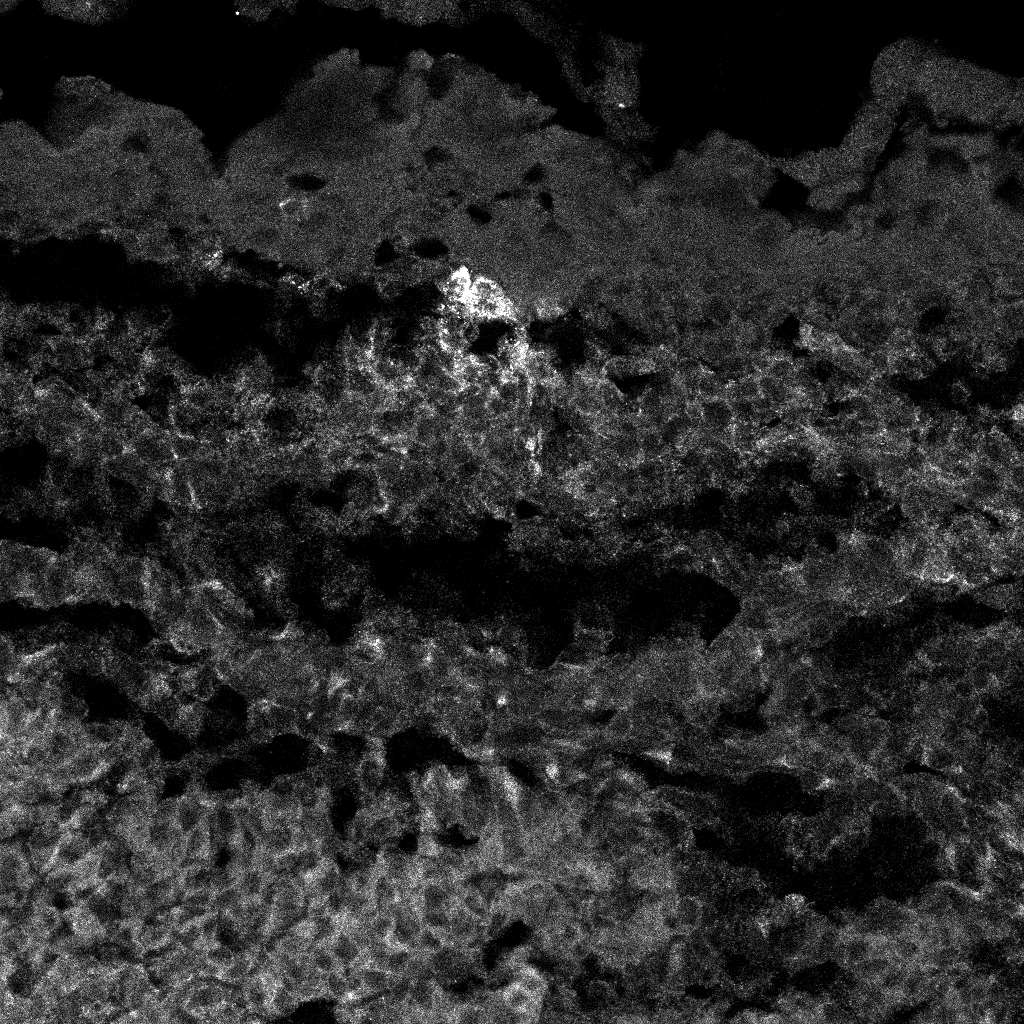

Supplement: Supplementary file 8 — Source Data Fig. 7 [file 44318_2024_39_MOESM8_ESM.zip › Figure 7/7E/MMP9-BAY11 treated cKO p65NFKBgreen MMP9red 40X.tif]

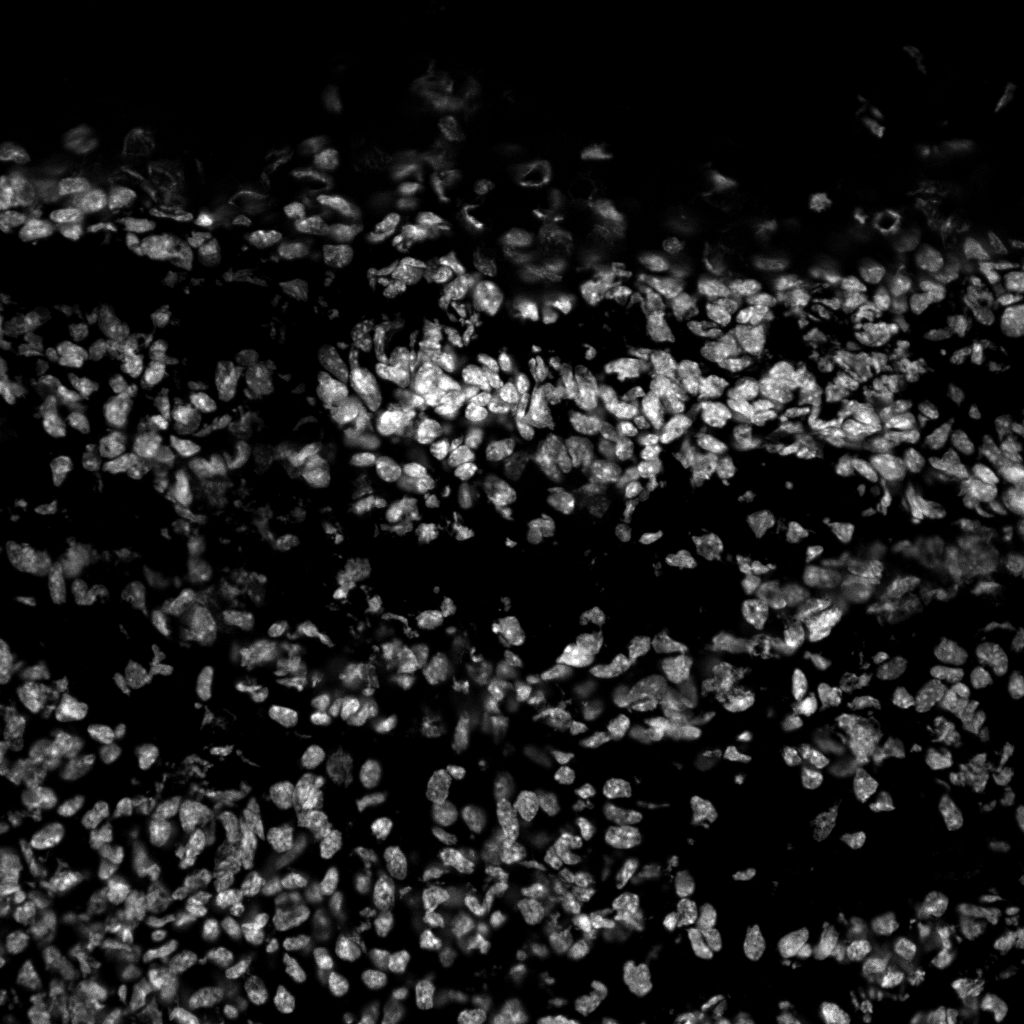

Supplement: Supplementary file 8 — Source Data Fig. 7 [file 44318_2024_39_MOESM8_ESM.zip › Figure 7/7E/DAPI-BAY11 treated cKO p65NFKBgreen MMP9red 40X.tif]

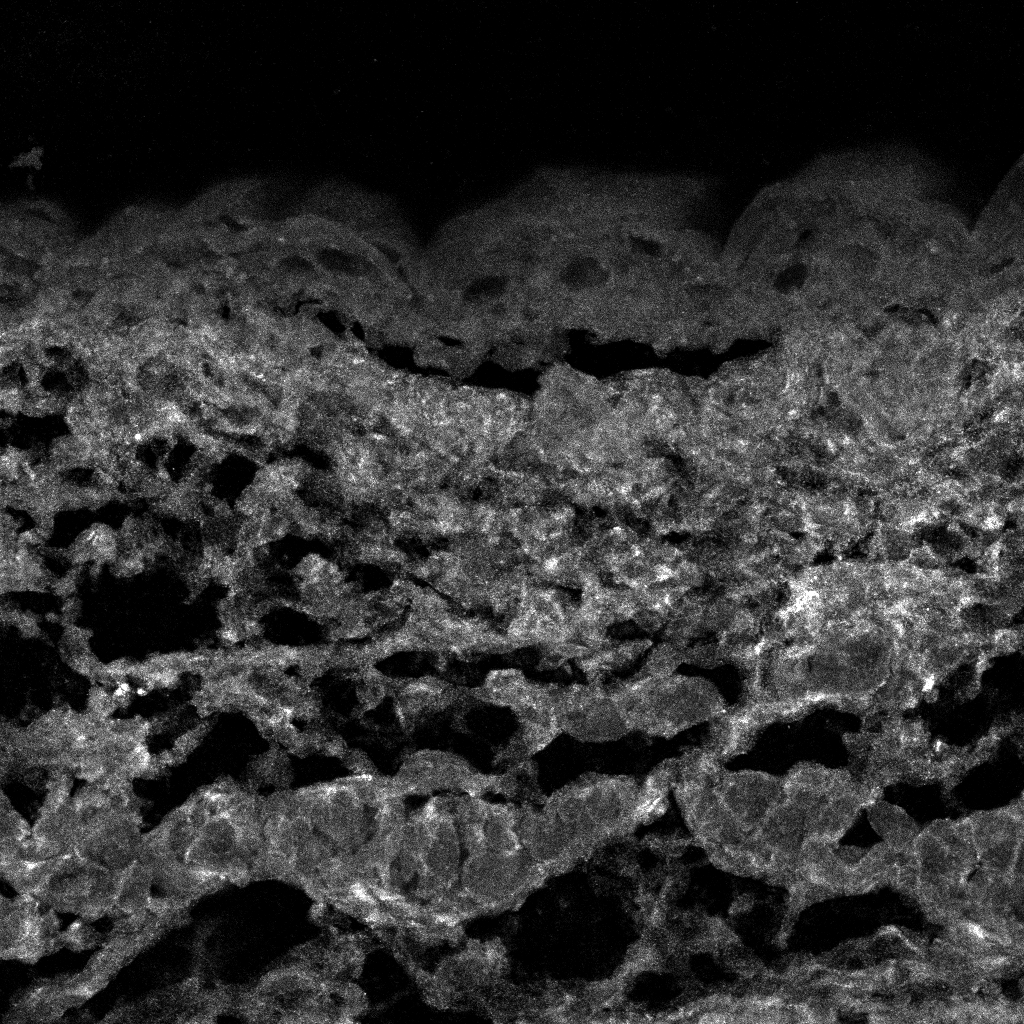

Supplement: Supplementary file 8 — Source Data Fig. 7 [file 44318_2024_39_MOESM8_ESM.zip › Figure 7/7E/MMP9-Vehicle treated cKO p65NFKBgreen MMP9red 40X.tif]

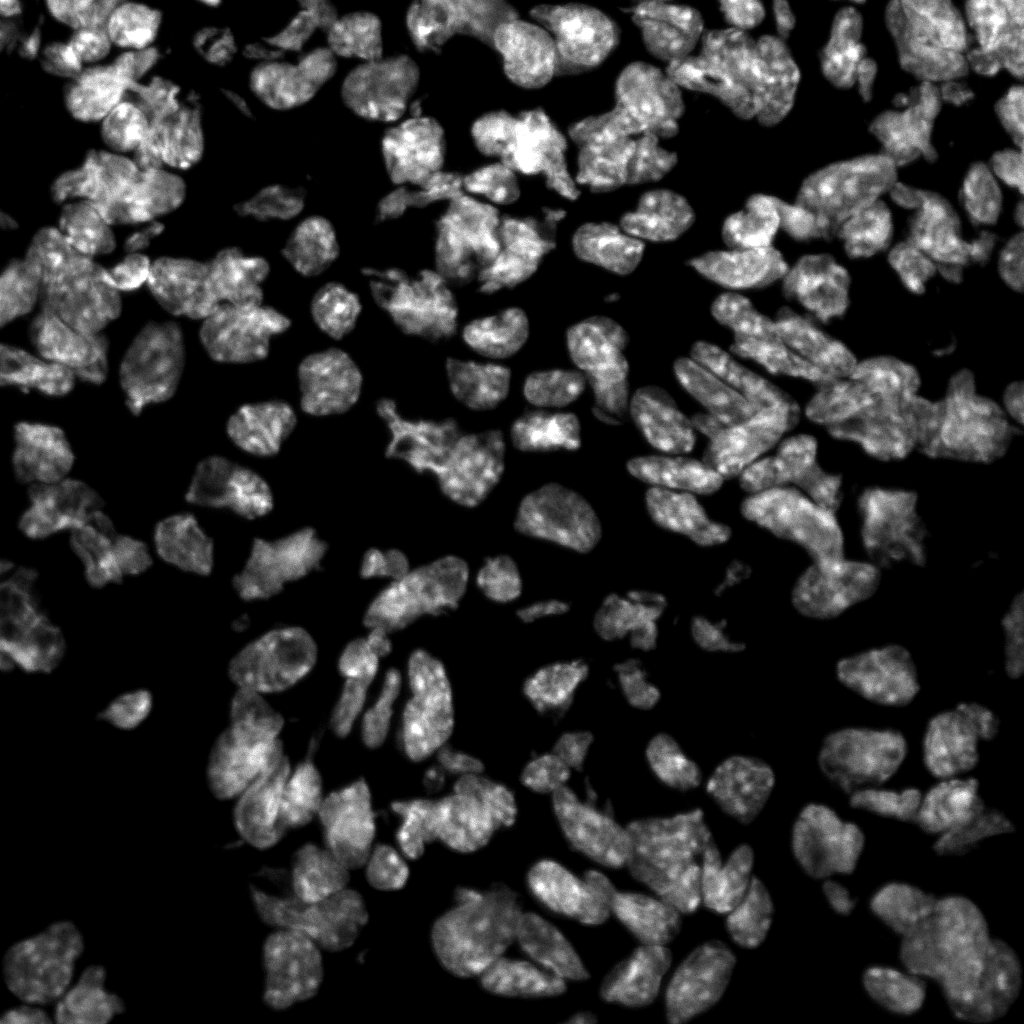

Supplement: Supplementary file 8 — Source Data Fig. 7 [file 44318_2024_39_MOESM8_ESM.zip › Figure 7/7A/DMSO KO F480green NFKBred 40X 2.41X]

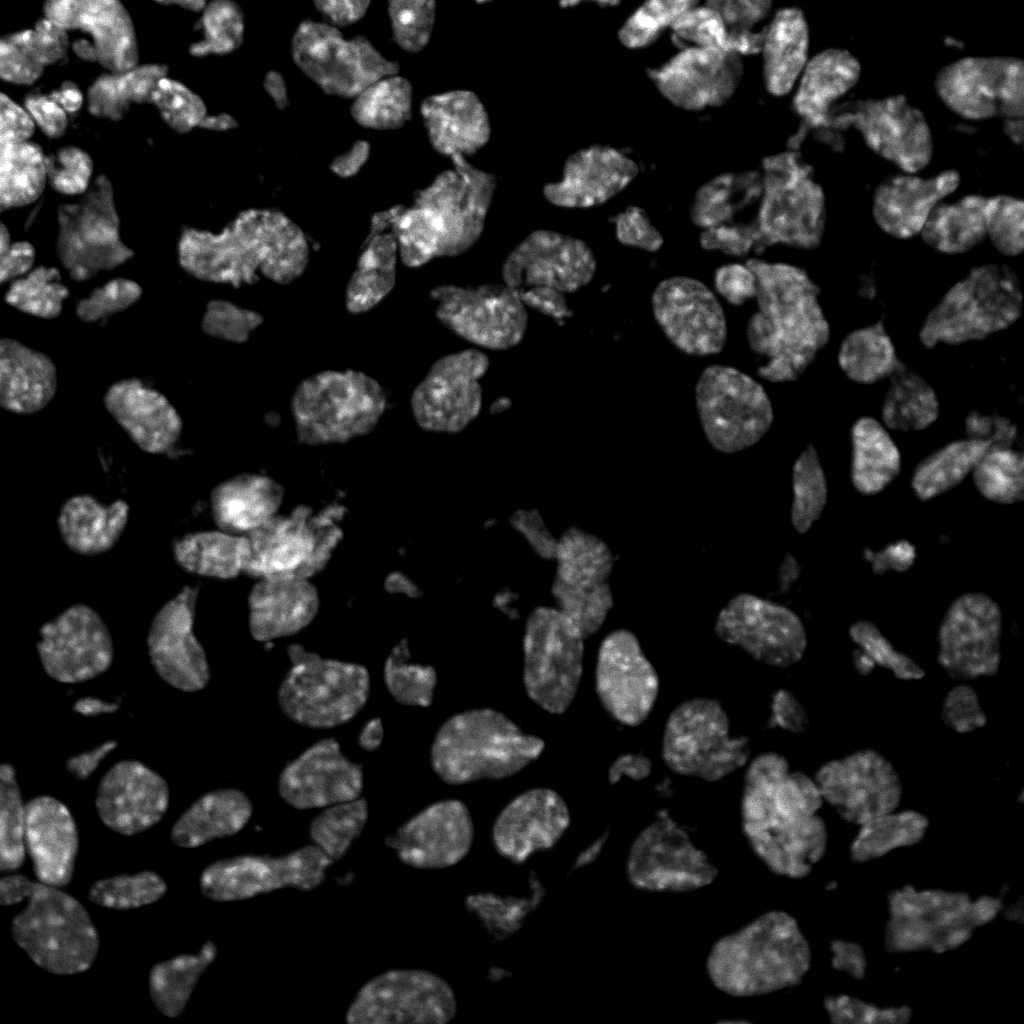

Supplement: Supplementary file 8 — Source Data Fig. 7 [file 44318_2024_39_MOESM8_ESM.zip › Figure 7/7A/AZD3965 KO F480green NFKBred 40X 2.41X]

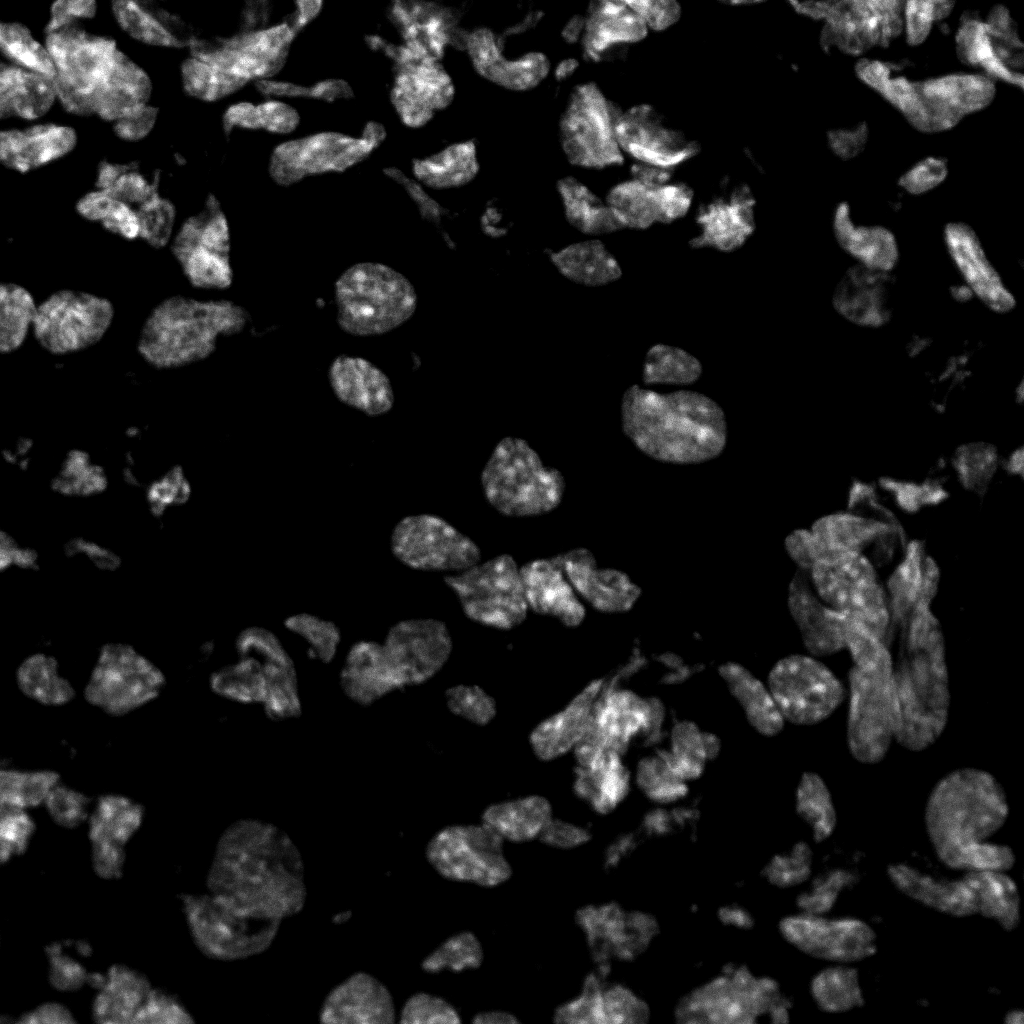

Supplement: Supplementary file 8 — Source Data Fig. 7 [file 44318_2024_39_MOESM8_ESM.zip › Figure 7/7A/SYRO KO F480green NFKBred 40X 2.41X]

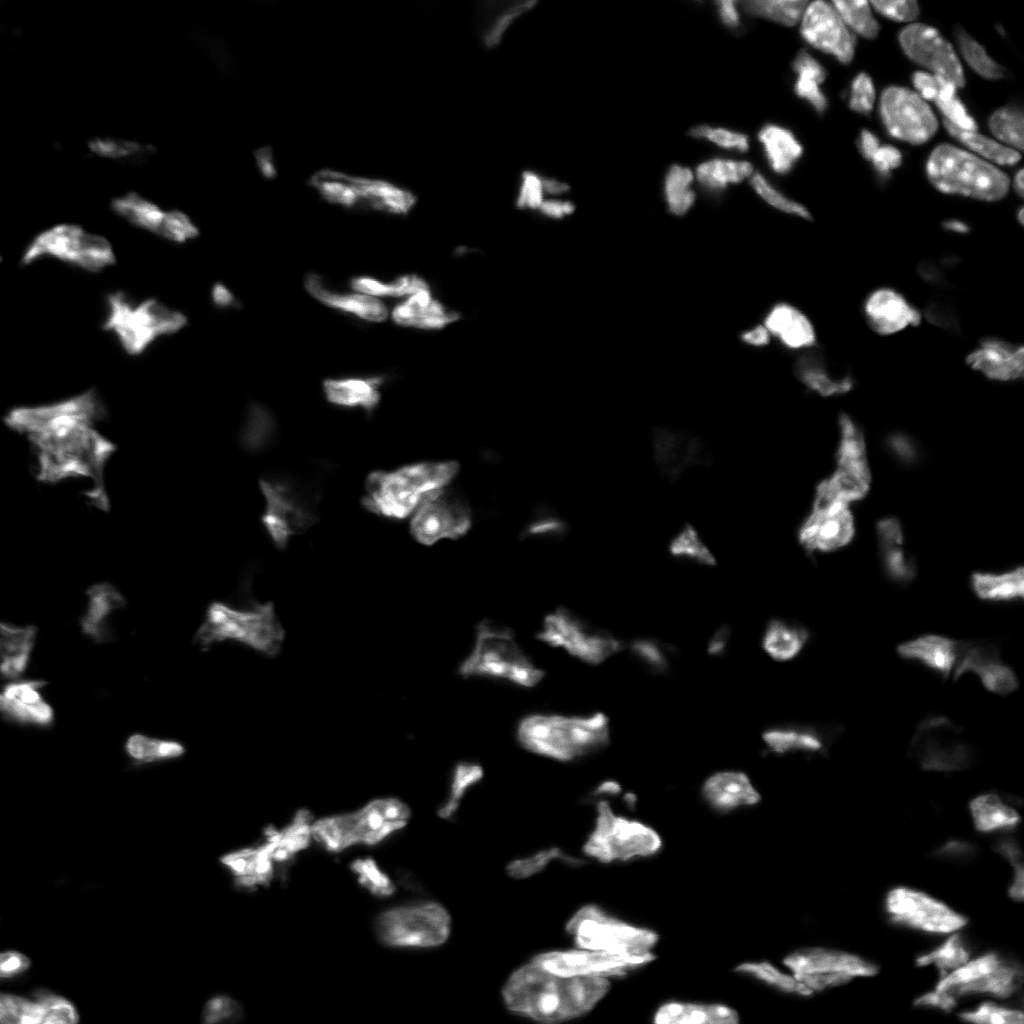

Supplement: Supplementary file 9 — Source Data Fig. 8 [file 44318_2024_39_MOESM9_ESM.zip › Figure 8/8B/2 month Imiquimod NFKBred F480green 40x.tif]

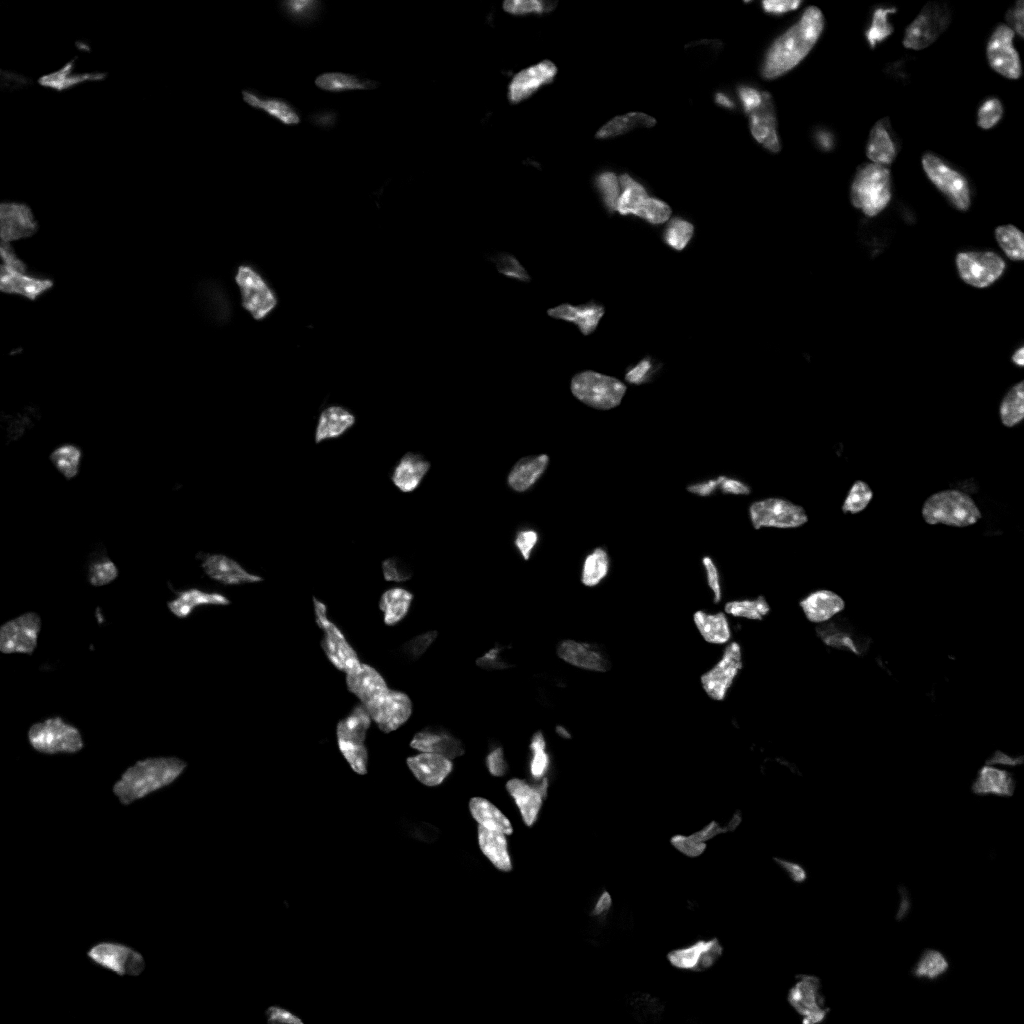

Supplement: Supplementary file 9 — Source Data Fig. 8 [file 44318_2024_39_MOESM9_ESM.zip › Figure 8/8B/2 month Vaseline F480green CSred 40X 1.8X]

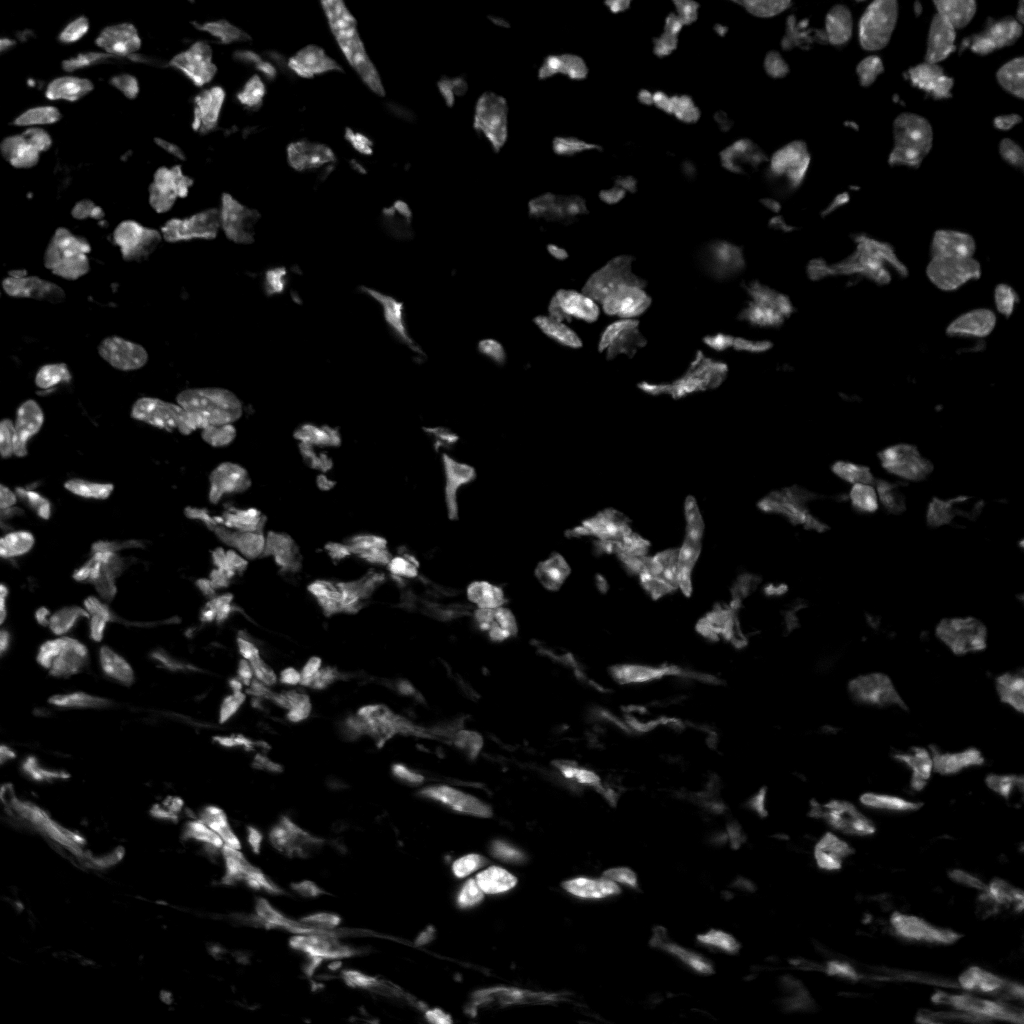

Supplement: Supplementary file 9 — Source Data Fig. 8 [file 44318_2024_39_MOESM9_ESM.zip › Figure 8/8B/2 month Imiquimod F480green IDH1red 40X 1.8X]

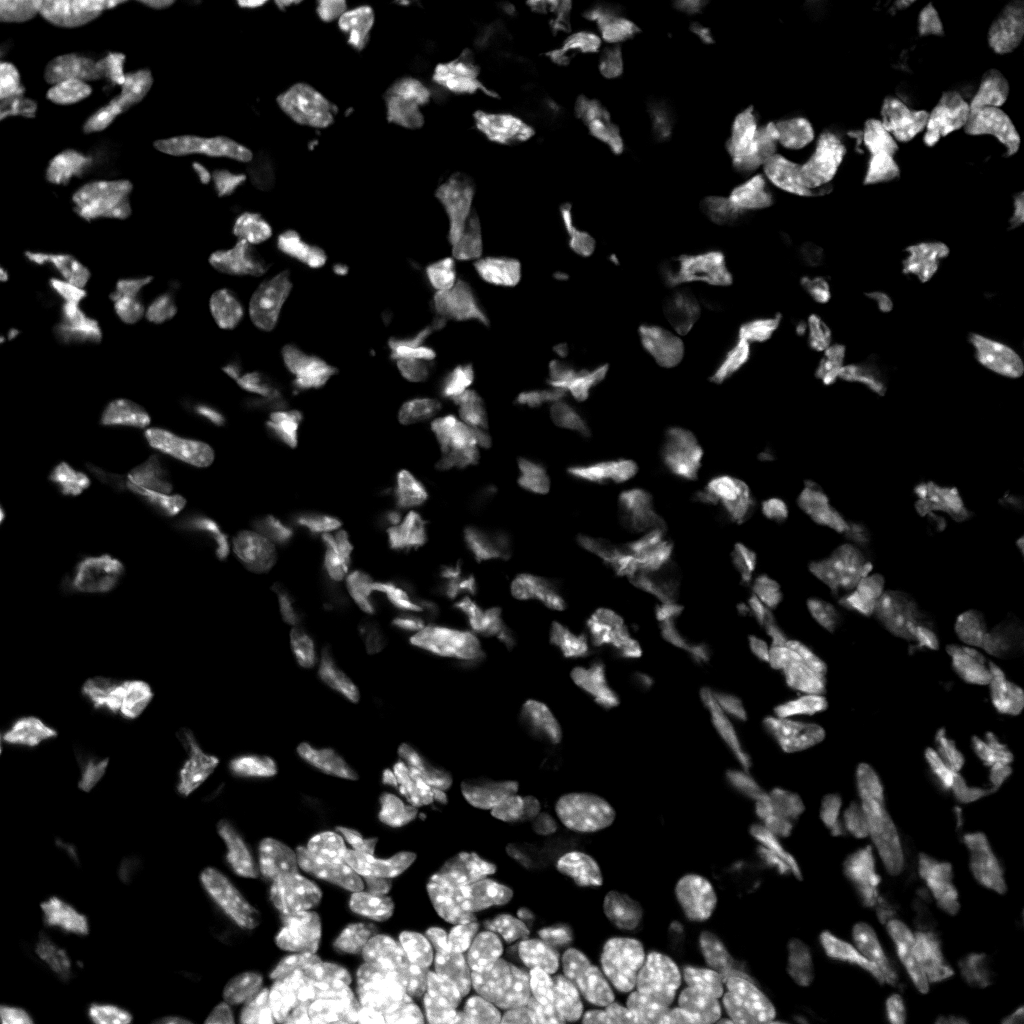

Supplement: Supplementary file 9 — Source Data Fig. 8 [file 44318_2024_39_MOESM9_ESM.zip › Figure 8/8B/2 month Imiquimod F480green CSred 40X 1.tif]

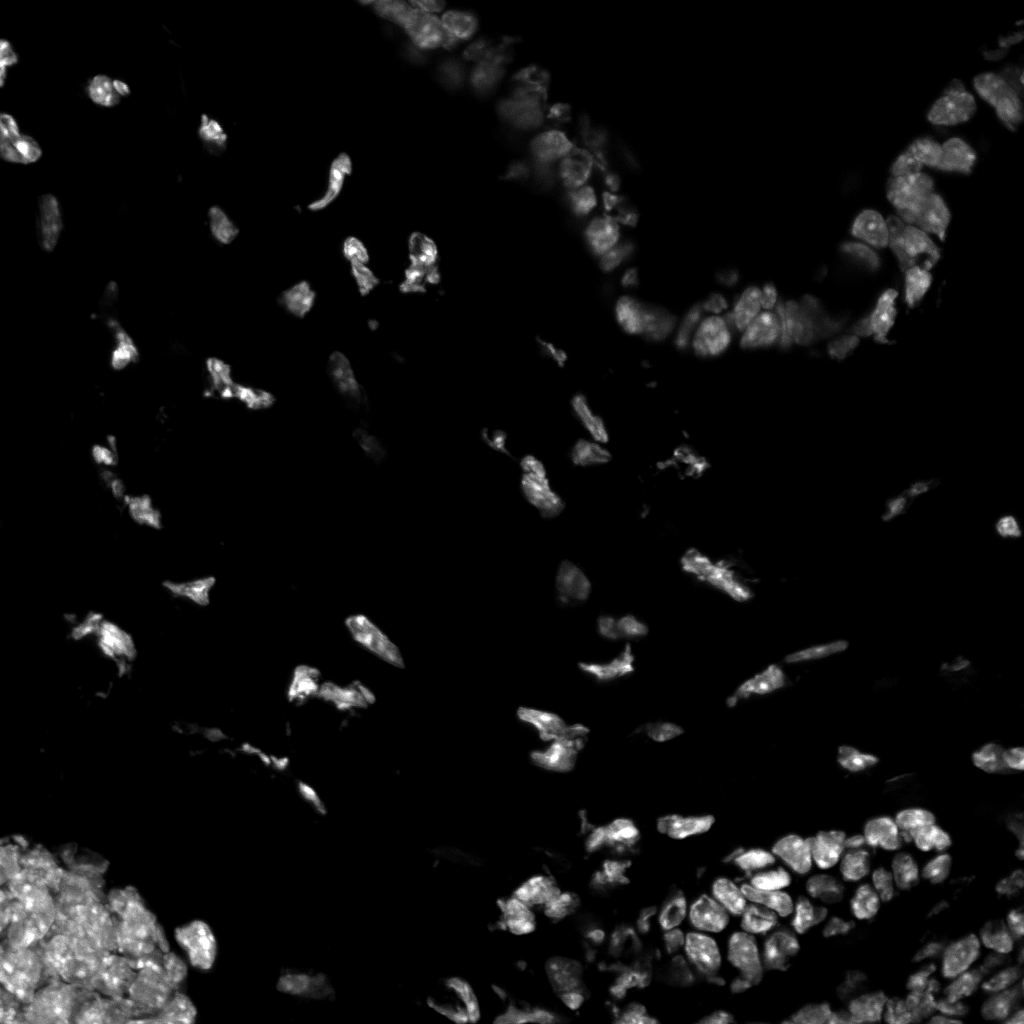

Supplement: Supplementary file 9 — Source Data Fig. 8 [file 44318_2024_39_MOESM9_ESM.zip › Figure 8/8B/2 month Vaseline F480green IDH1red 40X.tif]

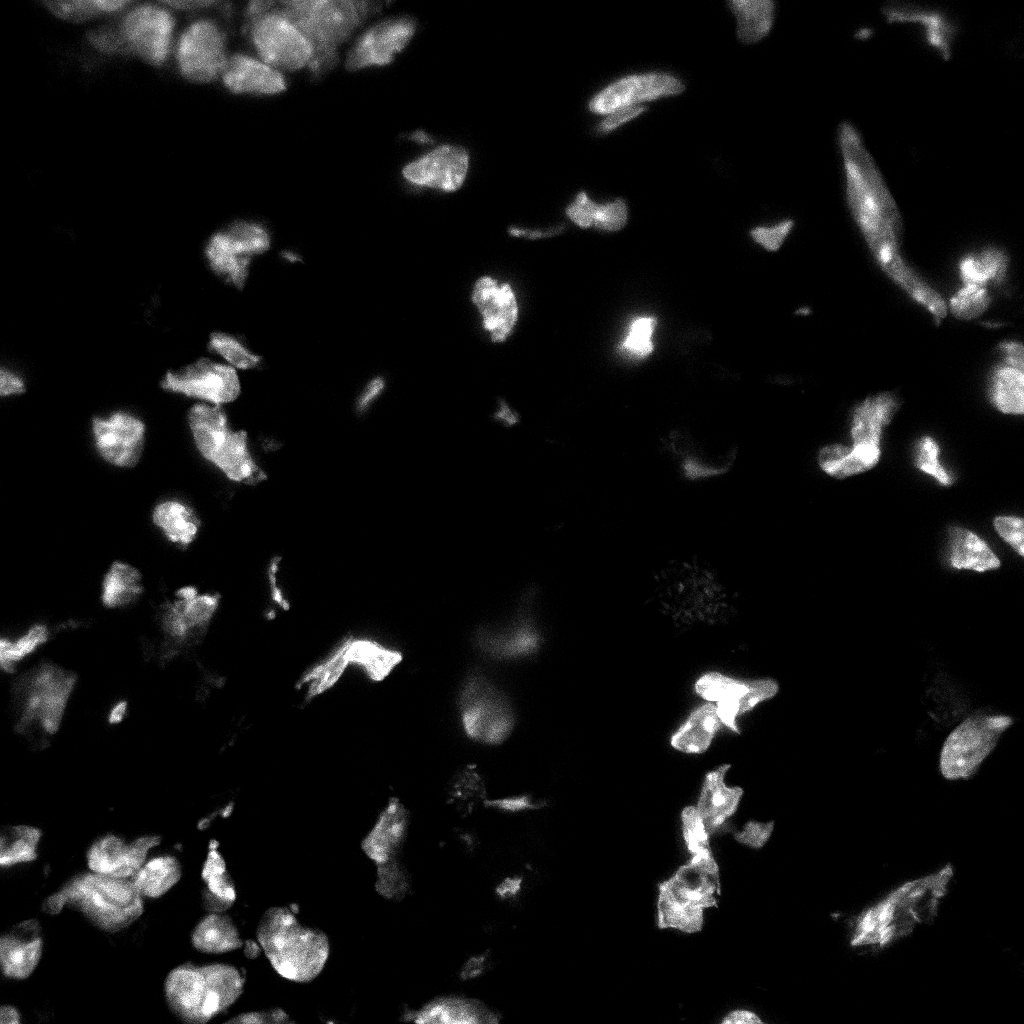

Supplement: Supplementary file 9 — Source Data Fig. 8 [file 44318_2024_39_MOESM9_ESM.zip › Figure 8/8B/2 month Vaseline F480green MCT1red 60X 1.8X]

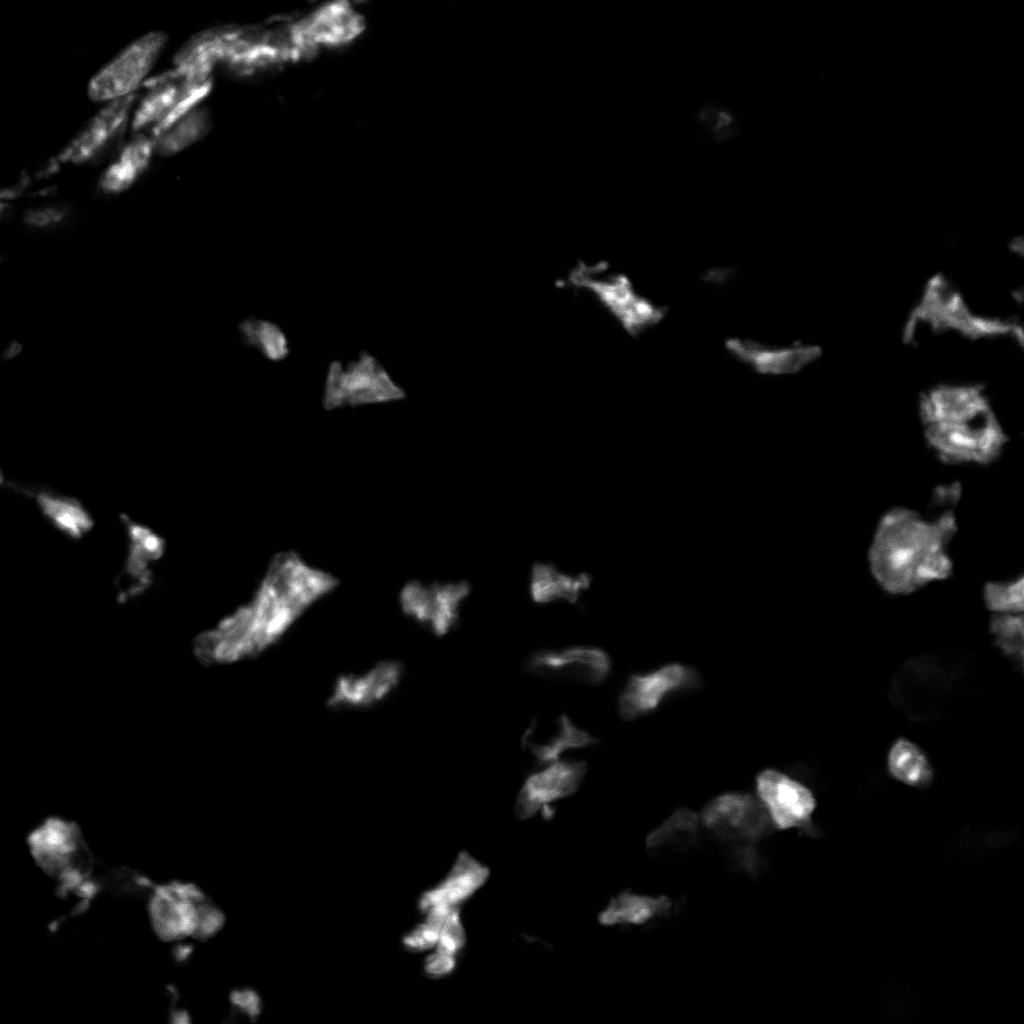

Supplement: Supplementary file 9 — Source Data Fig. 8 [file 44318_2024_39_MOESM9_ESM.zip › Figure 8/8B/2 month Vaseline NFKBred F480green 40X.tif]

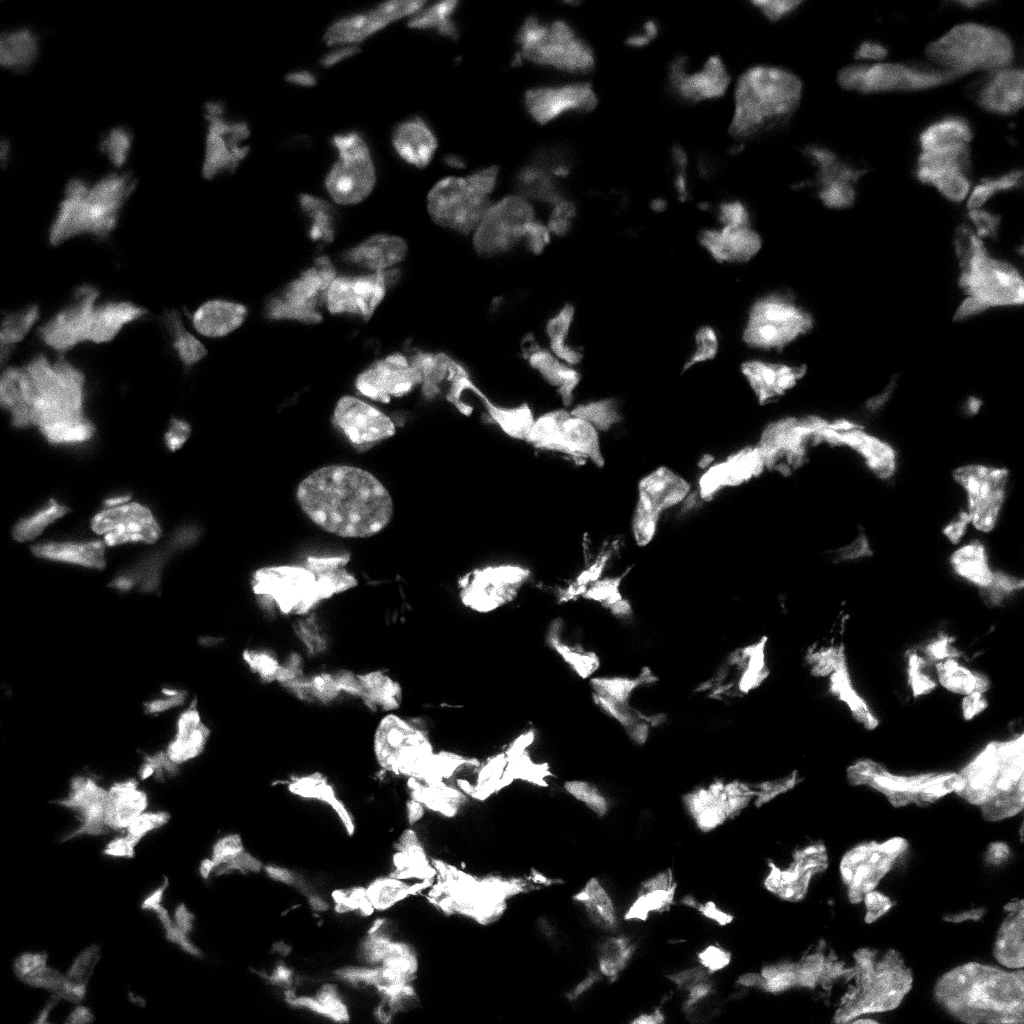

Supplement: Supplementary file 9 — Source Data Fig. 8 [file 44318_2024_39_MOESM9_ESM.zip › Figure 8/8B/2 month Imiquimod F480green MCT1red 60X 1.8X]

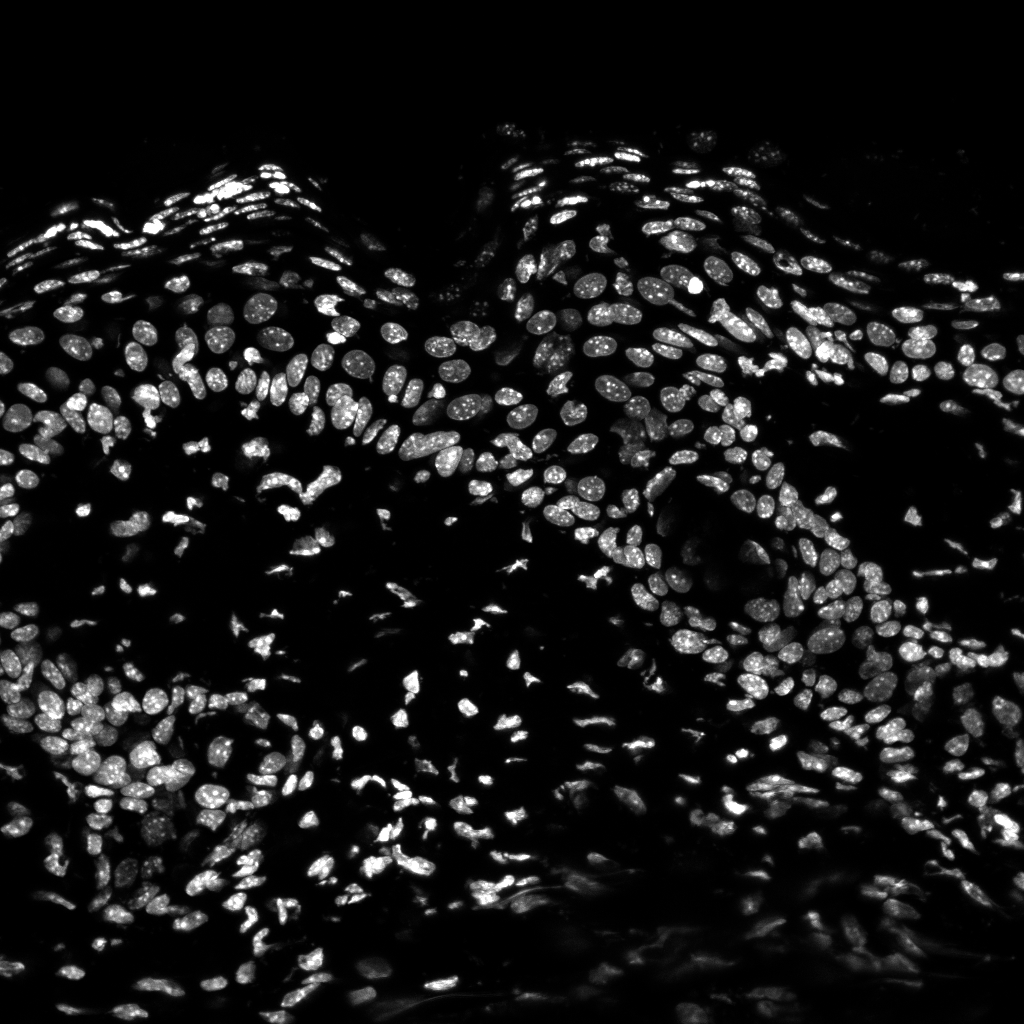

Supplement: Supplementary file 9 — Source Data Fig. 8 [file 44318_2024_39_MOESM9_ESM.zip › Figure 8/8A/GLUT1 staining/2 month Imiquimod GLUT1green 40X.tif]
