# Supplementary figures and images for: Metabolic rewiring of macrophages by epidermal-derived lactate promotes sterile inflammation in the murine skin (part 2 of 2)
Source: EMBO J. 2024 Feb 28;43(7):1113–34. doi: 10.1038/s44318-024-00039-y (PMC10987662; doi:10.1038/s44318-024-00039-y)

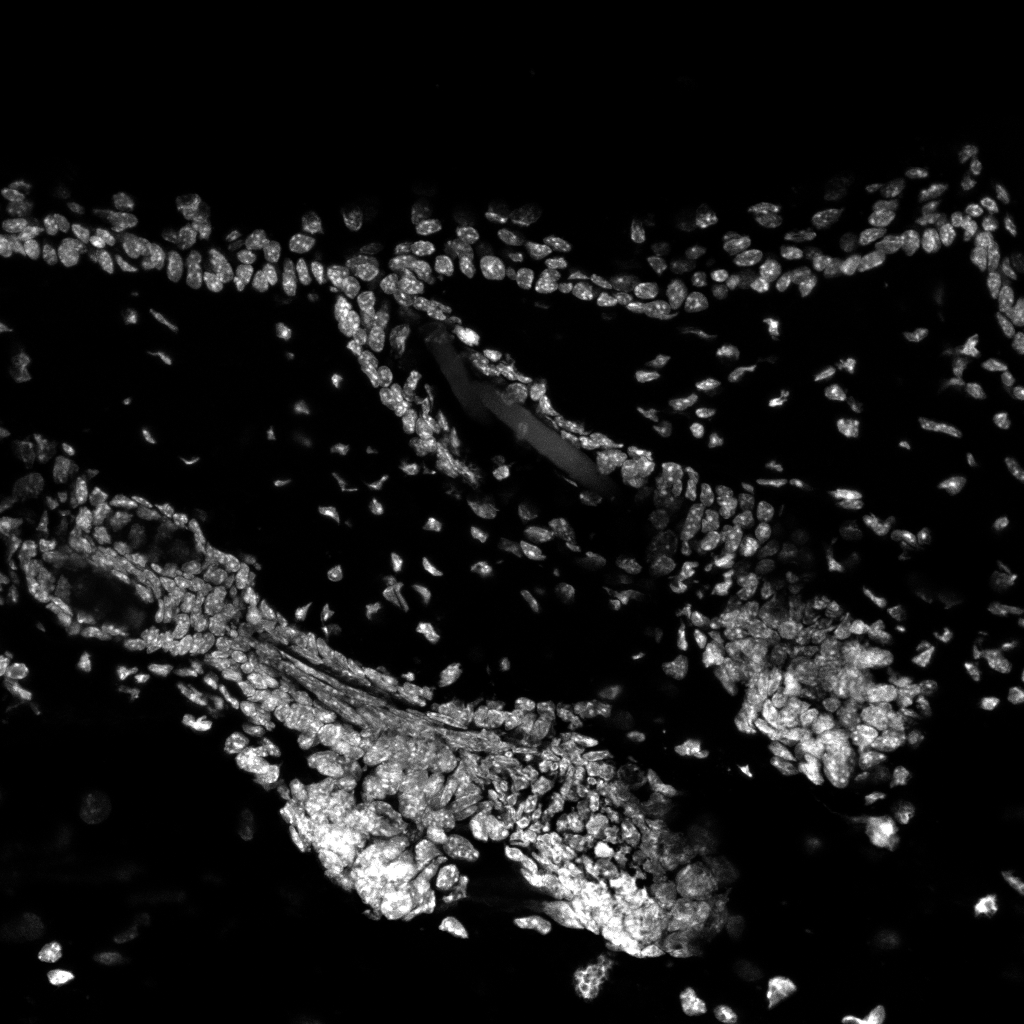

Supplement: Supplementary file 9 — Source Data Fig. 8 [file 44318_2024_39_MOESM9_ESM.zip › Figure 8/8A/GLUT1 staining/2 month Vaseline GLUT1green 40X.tif]

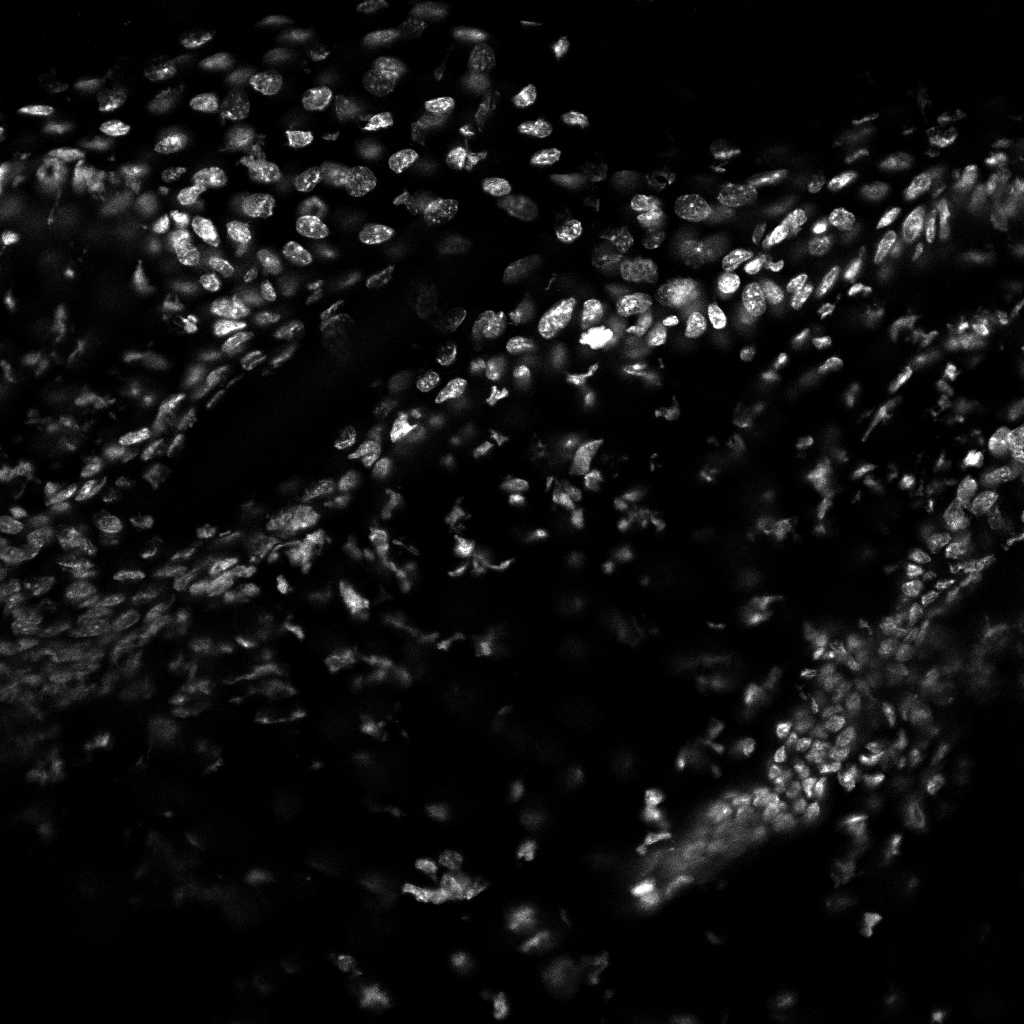

Supplement: Supplementary file 9 — Source Data Fig. 8 [file 44318_2024_39_MOESM9_ESM.zip › Figure 8/8A/MCT4 staining/2 month Imiquimod MCT4 40X.tif]

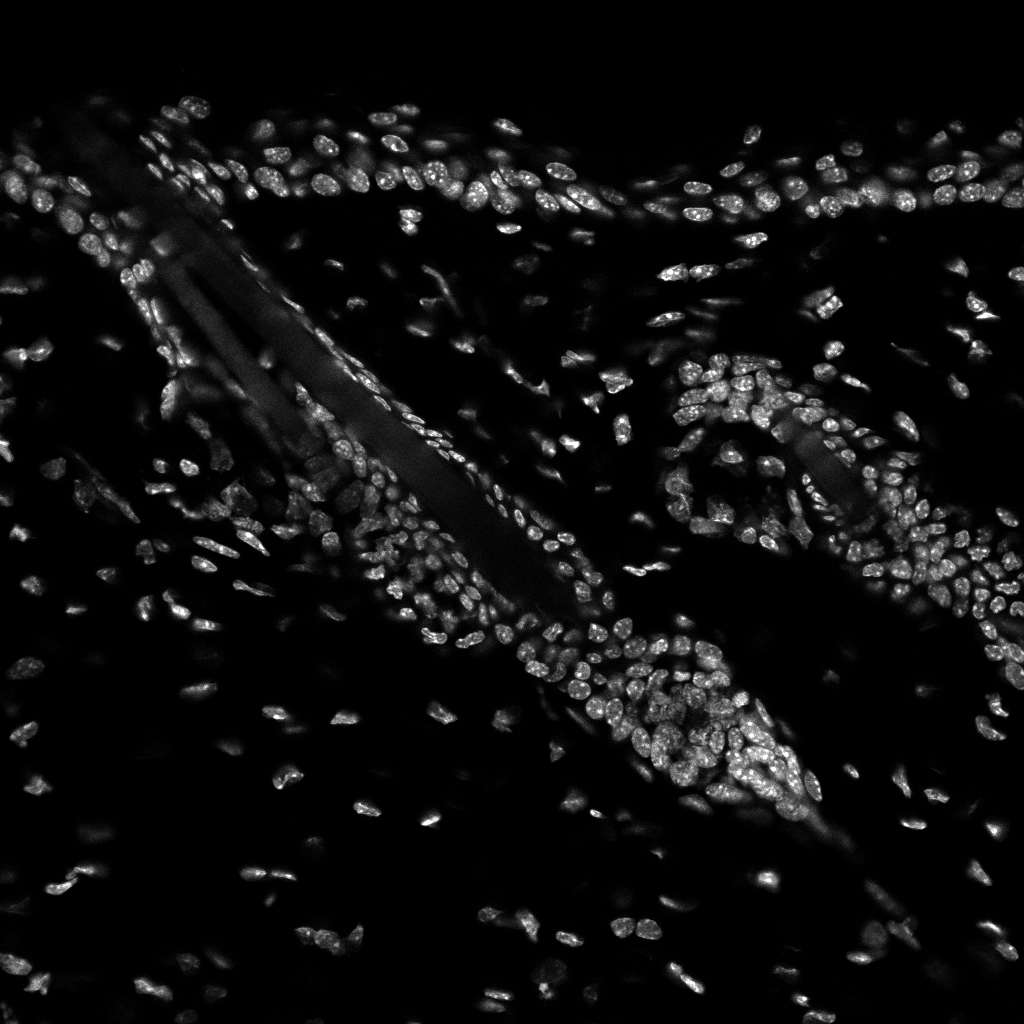

Supplement: Supplementary file 9 — Source Data Fig. 8 [file 44318_2024_39_MOESM9_ESM.zip › Figure 8/8A/MCT4 staining/2 month Vaseline MCT4red 40X.tif]

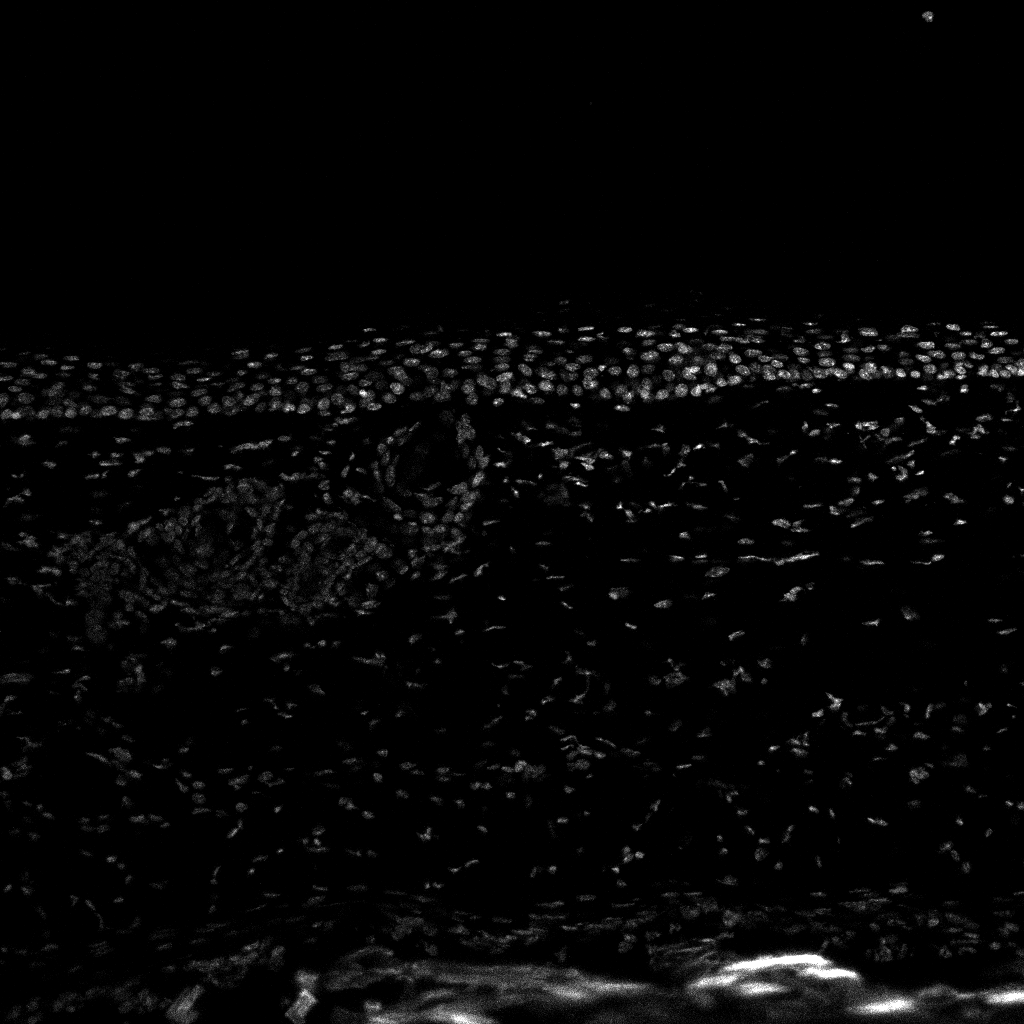

Supplement: Supplementary file 9 — Source Data Fig. 8 [file 44318_2024_39_MOESM9_ESM.zip › Figure 8/8E/MMP9 staining/2 month SYRO TREATED MMP9green 20X.tif]

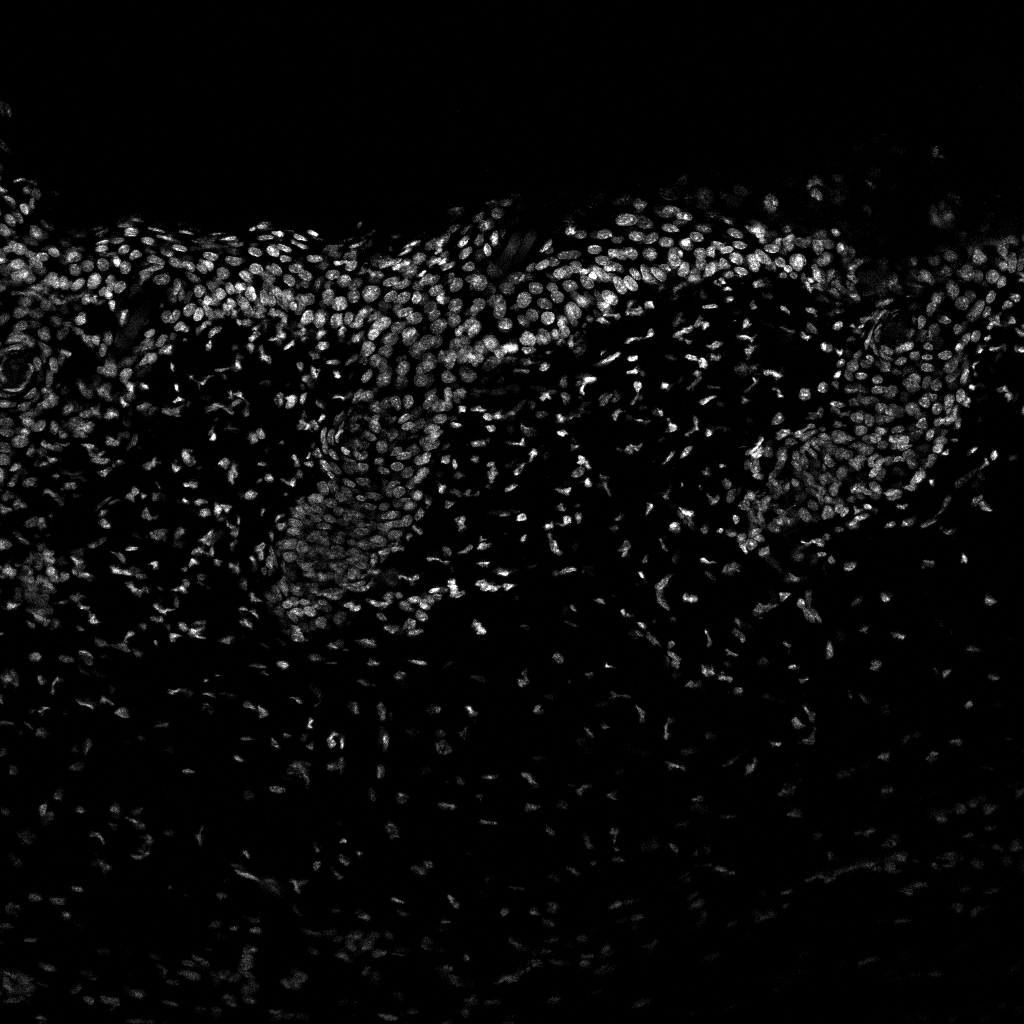

Supplement: Supplementary file 9 — Source Data Fig. 8 [file 44318_2024_39_MOESM9_ESM.zip › Figure 8/8E/MMP9 staining/2 month DMSO TREATED MMP9green 20X.tif]

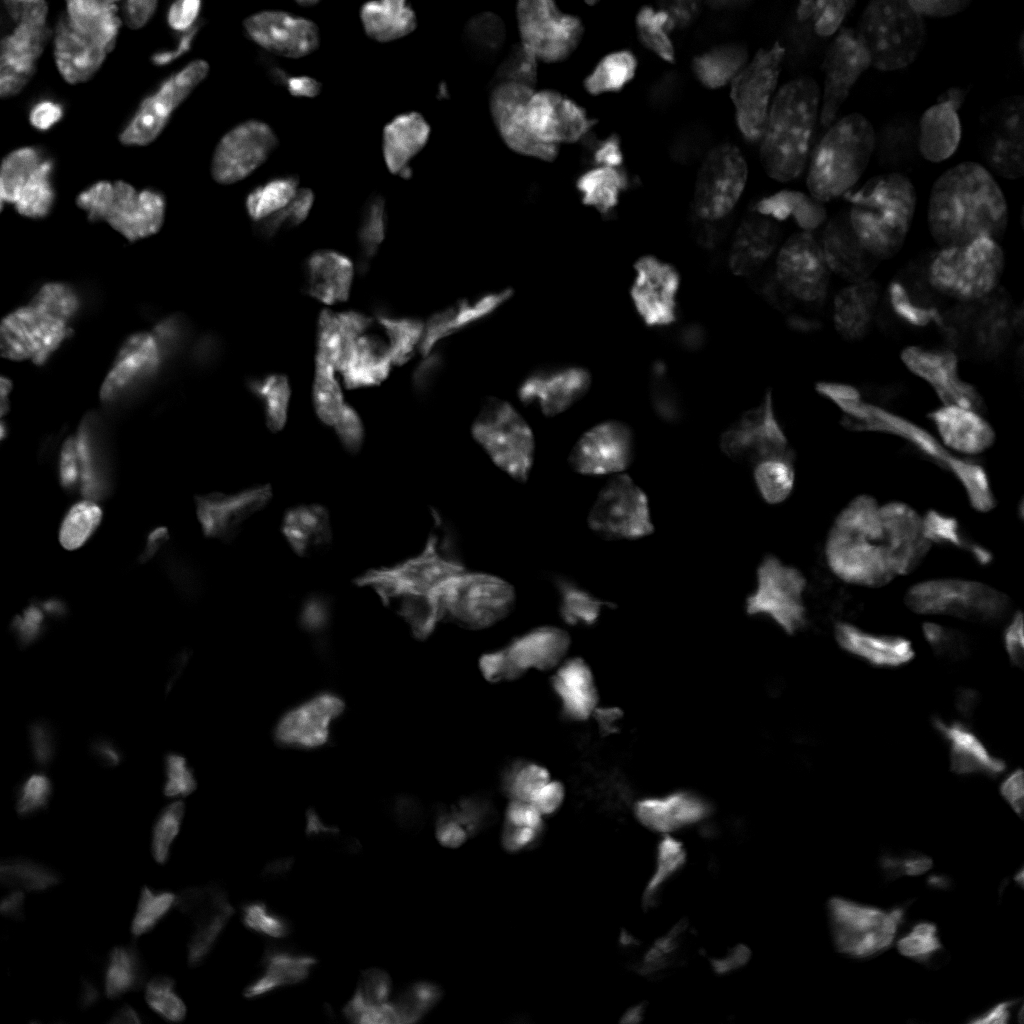

Supplement: Supplementary file 9 — Source Data Fig. 8 [file 44318_2024_39_MOESM9_ESM.zip › Figure 8/8E/F480 NFKB staining/2 month DMSO F480green NFKBred 40X 2.41X]

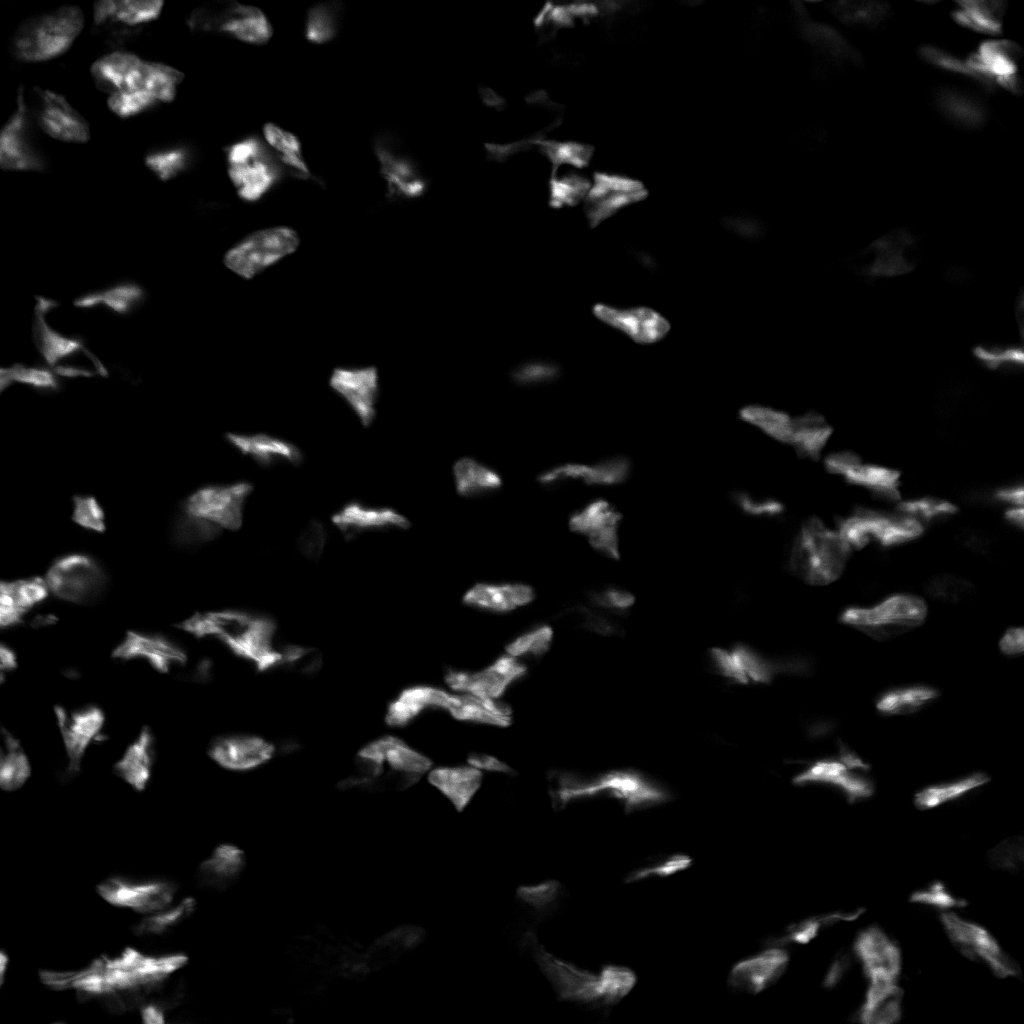

Supplement: Supplementary file 9 — Source Data Fig. 8 [file 44318_2024_39_MOESM9_ESM.zip › Figure 8/8E/F480 NFKB staining/2 month SYRO F480green NFKBred 40X 2.41X]

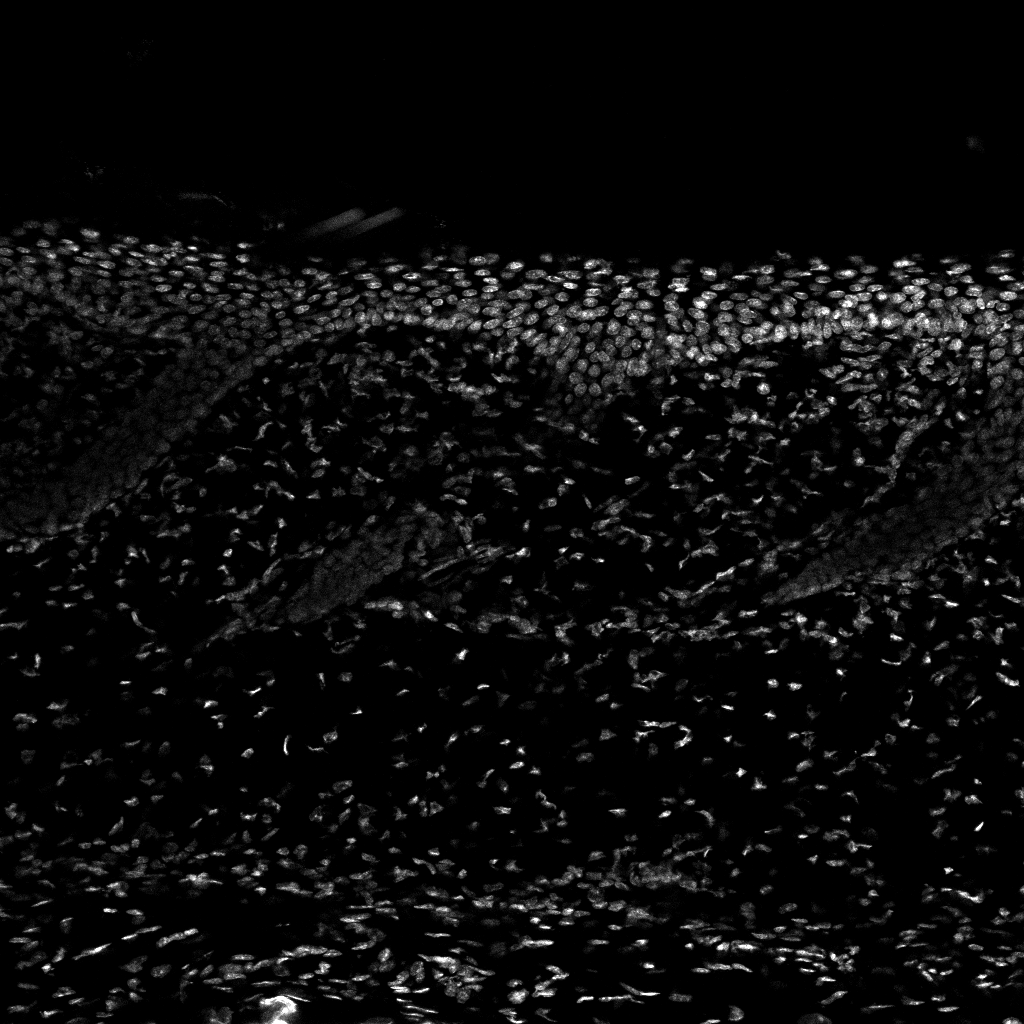

Supplement: Supplementary file 9 — Source Data Fig. 8 [file 44318_2024_39_MOESM9_ESM.zip › Figure 8/8E/ITGB4 KI67 staining/2 month DMSO TREATED ITGB4green KI67red 20X.tif]

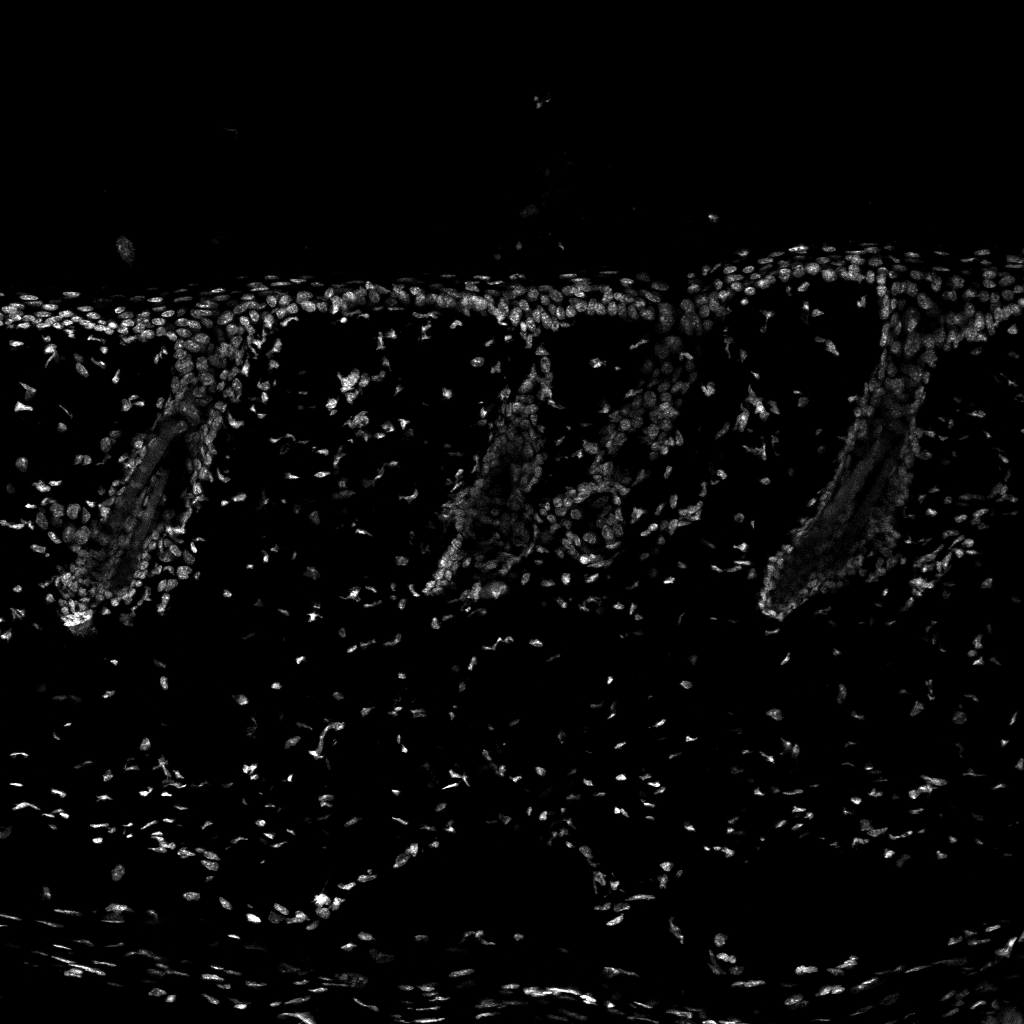

Supplement: Supplementary file 9 — Source Data Fig. 8 [file 44318_2024_39_MOESM9_ESM.zip › Figure 8/8E/ITGB4 KI67 staining/2 month SYRO TREATED ITGB4green KI67red 20X.tif]

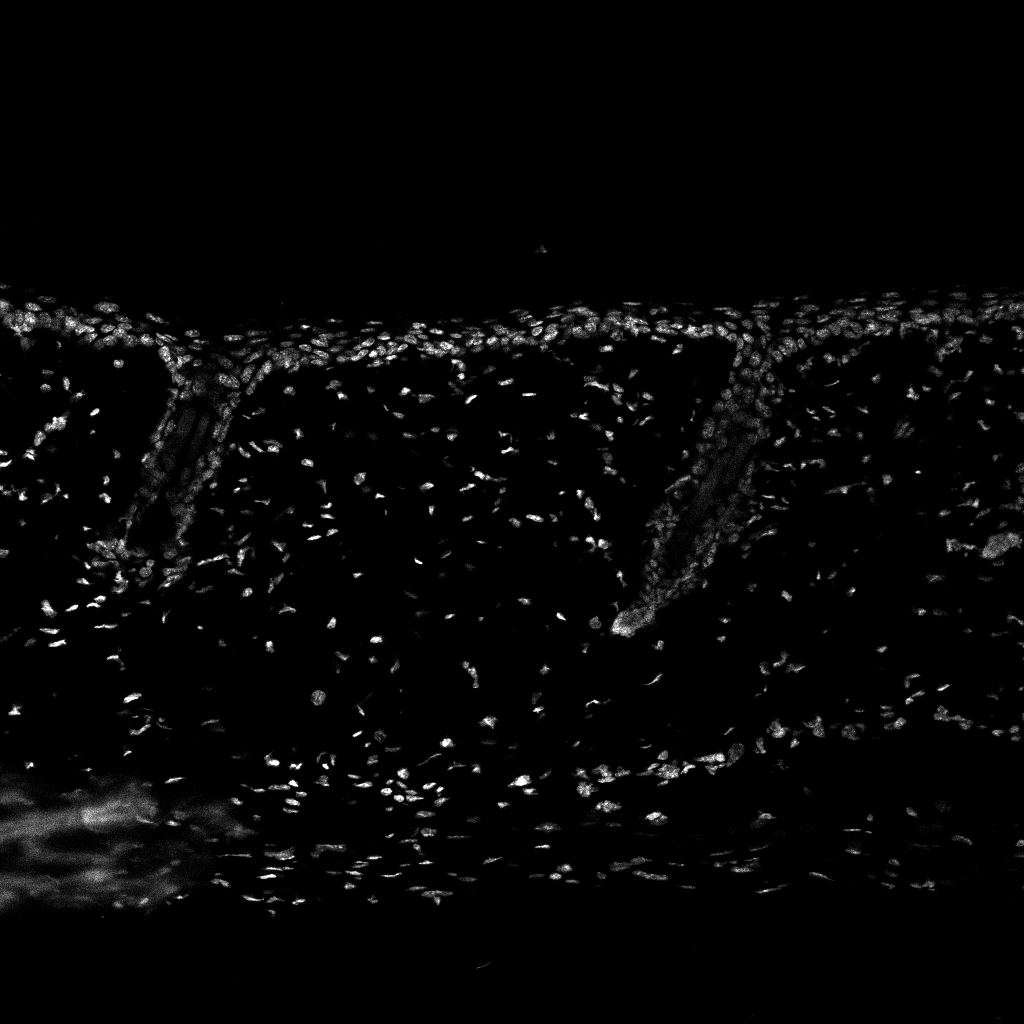

Supplement: Supplementary file 9 — Source Data Fig. 8 [file 44318_2024_39_MOESM9_ESM.zip › Figure 8/8E/F480 staining/2 month SYRO TREATED F480green 20X.tif]

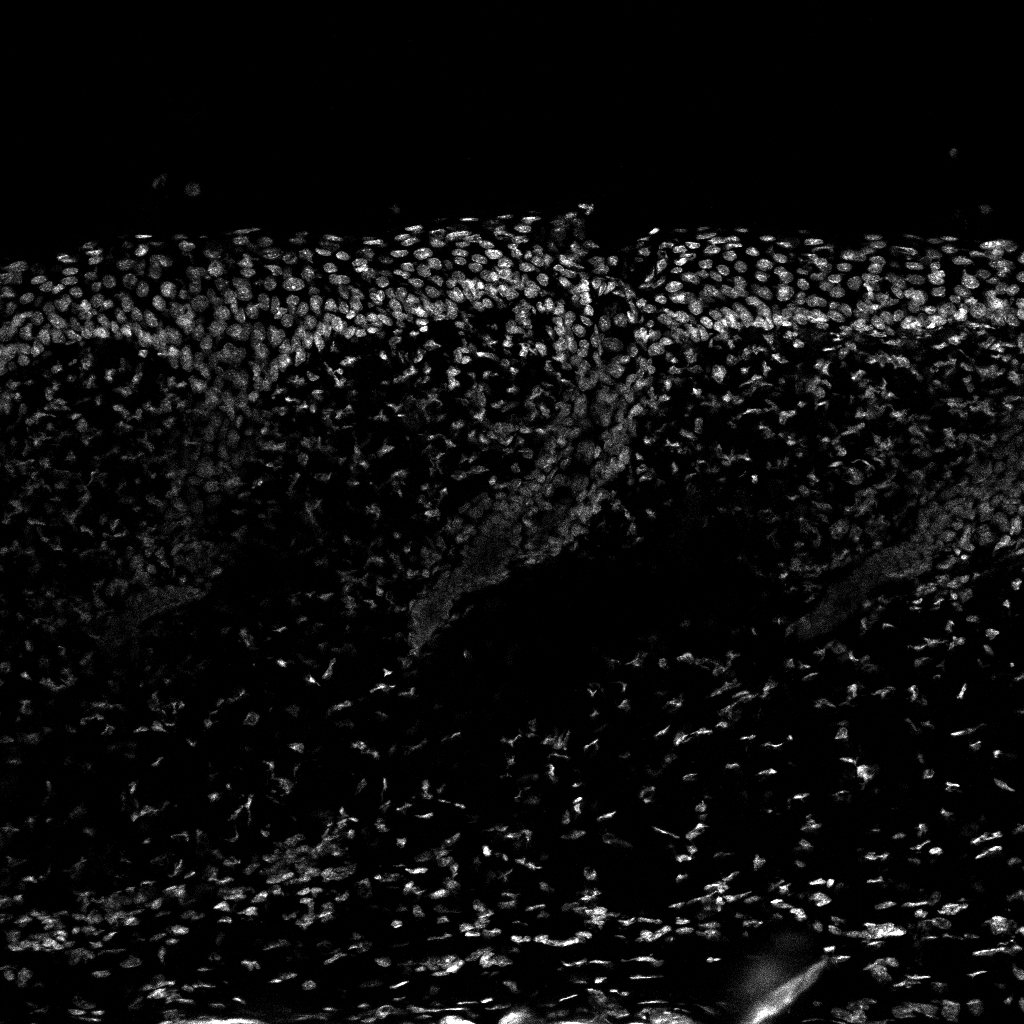

Supplement: Supplementary file 9 — Source Data Fig. 8 [file 44318_2024_39_MOESM9_ESM.zip › Figure 8/8E/F480 staining/2 month DMSO TREATED F480green 20X.tif]
